# T-REx: Transcriptome analysis webserver for RNA-seq Expression data

**DOI:** 10.1186/s12864-015-1834-4

**Published:** 2015-09-03

**Authors:** Anne de Jong, Sjoerd van der Meulen, Oscar P. Kuipers, Jan Kok

**Affiliations:** Molecular Genetics, University of Groningen, Nijenborgh 7, 9747AG Groningen, The Netherlands; Top Institute Food and Nutrition (TIFN), Nieuwe Kanaal 9A, 6709 PA Wageningen, The Netherlands; Molecular Genetics, Groningen Biomolecular Sciences and Biotechnology Institute, University of Groningen, Groningen, The Netherlands

## Abstract

**Background:**

Transcriptomics analyses of bacteria (and other organisms) provide global as well as detailed information on gene expression levels and, consequently, on other processes in the cell. RNA sequencing (RNA-seq) has over the past few years become the most accurate method for global transcriptome measurements and for the identification of novel RNAs. This development has been accompanied by advances in the bioinformatics methods, tools and software packages that deal with the analysis of the large data sets resulting from RNA-seq efforts.

**Results:**

Based on years of experience in analyzing transcriptome data, we developed a user-friendly webserver that performs the statistical analysis on the gene expression values generated by RNA-seq. It also provides the user with a whole range of data plots. We benchmarked our RNA-seq pipeline, T-REx, using a case study of CodY mutants of *Bacillus subtilis* and show that it could easily and automatically reproduce the statistical analysis of the cognate publication. Furthermore, by mining the correlation matrices, k-means clusters and heatmaps generated by T-REx we observed interesting gene-behavior and identified sub-groups in the CodY regulon.

**Conclusion:**

T-REx is a parameter-free statistical analysis pipeline for RNA-seq gene expression data that is dedicated for use by biologists and bioinformaticians alike. The tables and figures produced by T-REx are in most cases sufficient to accurately mine the statistical results. In addition to the stand-alone version, we offer a user-friendly webserver that only needs basic input (http://genome2d.molgenrug.nl).

**Electronic supplementary material:**

The online version of this article (doi:10.1186/s12864-015-1834-4) contains supplementary material, which is available to authorized users.

## Background

Measuring mRNA levels in cells or tissues is being performed ever since the introduction of Northern blot hybridization. Implementation of DNA-microarray technology has allowed to measure gene expression at a genome-wide scale. Although DNA-microarrays are still being used, the technique is now almost fully replaced by next-generation (RNA) sequencing (RNA-seq). This relatively new method can be used to determine absolute gene expression levels and is far more accurate than DNA-microarraying, which commonly generates ratio-based data. Analysis of RNA-seq data is in principle divided into two stages. The first step involves the quality control and mapping of the sequence reads to an annotated reference genome. Command line tools such as SAMtools [1] and BEDtools [2] are commonly used but user friendly software packages such as RockHopper [3] and NGS-Trex [4] have also been developed. This generates gene (RNA) expression values such as Reads Per Kilobase per Million reads (RPKM), Fragments Per Kilobase per Million (FPKMs), Counts Per Million (CPM) or other gene expression units. The second step entails statistical and biological analyses of the transcriptome data using tools such as EdgeR [5], DEseq [6] and others [7]. These investigations could involve the analysis of differential gene expression between two samples, but they can also be more complex such as in the analysis of data obtained from times series experiments or of multiple experiments from multiple time points. To blend the various approaches into one common analysis method, factorial design is the most favorable procedure used for the analysis of DNA-microarray data (LimmeR, [8]) as well as for RNA-seq data analysis (EdgeR and DEseq). Factorial design offers flexibility in controlling how to perform the statistical analyses. Once the factorial design has been made, six analysis steps are generally executed; i) normalization and scaling of the gene expression values, ii) global analysis of the experiments using e.g., Principal Component Analysis (PCA), iii) differential expression of genes between experiments, iv) clustering of genes expression levels and/or ratios between experiments, v) studying the behavior of groups of genes of interest (classes), vi) functional analysis or gene-set enrichment. A variety of software packages can be used to perform the steps mentioned above but, due to issues regarding user-friendliness, these are usually practical mainly for bioinformaticians. The main topics in examining the huge amount of transcriptomics data obtained by RNA-seq are the choice of proper data analysis methods, the setting of suitable parameters and the conversion and combining of data generated in the different stages of analysis. The development of the RNA-seq analysis pipeline T-Rex and the choices we made with respect to the methods and parameters employed were based on an iterative process between bioinformaticians and biologists. In this article we introduce and describe this pipeline, T-REx, a user-friendly webserver to analyse RNA-seq-derived gene expression data that has been optimized for prokaryotes. In addition we offer the R-script, which gives the user full control over the parameters used in the statistical analyses.

## Implementation

The first steps in the statistical analysis of gene expression data are data normalization and determination of the genes that are differentially expressed between samples. To do this, the factorial design statistical method of the RNA-seq analysis R-package EdgeR [5] was chosen. Routines for clustering and plotting of graphics were derived from the open source software repository Bioconductor [9].

The pipeline (Additional file 1 and 2) requires raw RNA expression level data as an input for RNA-seq data analysis. RPKM, FPKM, TPM [10] or any other count values can be combined in one table and used as an input for T-REx. Also, DNA-microarray data containing gene (RNA) expression levels can be used. For the calculation of the *p*-values for differential expression the dispersion model of EdgeR is employed, which is optimized for the use of CPM values. Comparison of the differentially expressed genes using either CPM or RPKM values showed only differences in the TopHits genes close to cutoff values (*p*-value 0.05 and fold-change of 2). The second input file defines the factors that are used to describe the experiments and the replicates (Table 1A). A third file is used to define which comparisons (contrasts) should be made between the various experimental conditions (Table 1B). The researcher is offered an easy and flexible way to produce the results by simply adjusting the contrast file. Although these three files are enough to perform a complete statistical analysis of the dataset under study, the added value of T-REx is a fourth file, which allows focusing the analysis on one group or multiple groups of genes or RNAs (e.g., the regulons of CodY and CcpA in the example given below). To do this, the researcher pre-defines a Class file of groups of genes of interest (Table 1C. More details, examples and tutorials for creating the 4 files can be obtained from the T-REx webserver.

**Table 1:** Input files for the RNA-seq analysis pipeline

A) Factors			B) Contrasts	C) Classes		
Experiment	Strain	Time	A_F71Y-WT	BSU00490	green	CodY
WT1	WT	T1	B_R61K-WT	BSU01650	green	CodY
WT2	WT	T1	C_R61H-WT	BSU01660	green	CodY
F71Y1	A_F71Y	T1	Null-WT	BSU01670	green	CodY
F71Y2	A_F71Y	T1		BSU01680	green	CodY
R61K1	B_R61K	T2		etc…	…	…
R61K2	B_R61K	T2		BSU03981	red	CcpA
R61H1	C_R61K	T2		BSU03982	red	CcpA
R61H2	C_R61K	T2		BSU03990	red	CcpA
null1	Null	T2		BSU04160	red	CcpA
null2	Null	T2		BSU04470	red	CcpA
				etc…	…	…

A) File describing the experiments and containing information of experiment replicates, B) File with the comparisons (contrasts) to be made, C) File with groups of genes/RNAs of interest

Once T-REx is fed with the four input files, normalization and global analysis of the data will be performed and visualized in several graphs. These graphs include library sizes, box plots of normalized signals, a correlation matrix of experiments and a two-dimensional Multidimensional Scaling (MDS) plot of the samples to be studied. Although various normalization methods have been developed, we found the trimmed-median mean method (TMM) of EdgeR to be the most accurate for RNA-seq data derived from prokaryotes. Subsequently, statistical analysis of differential expression of genes of all contrasts (derived from Table 1B) is performed and the outcome is visualized in MA and volcano plots. In the MA plots traditionally used in DNA-microarray analysis gene expression is plotted against ratio values while volcano plots compute gene expression ratios against their *p*-values. In both of these dot-plots (in which dots represent genes) each dot gets a color as defined by the user in the Class file (Table 1C) while non-defined dots are colored black, allowing to easily identify the behavior of Class-defined genes. Genes can have an expression value of zero, e.g., when comparing two bacterial species in which one of the genes is absent. To prevent errors as a consequence of having to divide by zero, zero values are scaled to noise level. Thus, these genes will be flagged by a cross-sign in the MA and volcano plots.

K-means clustering [2, 11] is considered to be one of the most powerful methods to analyze behavior of gene expressions between dependent experiments, such as samples taken over a course of time from the same culture. However, defining the number of clusters in which the data can be divided is arbitrary. To tackle this issue, the analysis pipeline will estimate the number of clusters for a certain data set automatically. The number of genes is furthermore limited to include only those that have a fold change ≥ 2 and a *p*-value ≤ 0.05 (TopHits) in at least one of the contrasts. Subsequently, two additional groups of genes are defined, one group containing genes that show highly differential expression in at least one of the contrasts (HighFold: fold change ≥ 5 and *p*-value ≤ 0.01) and a group from which non changed genes are removed (no_background: fold change ≤ 1.4 and *p*-value ≥ 0.25). K-means and hierarchical clustering are executed on both the signal and the ratio data for all genes and classes. Plots of expression profiles and a correlation matrix are made for each Class group, which will show the relation of the genes within each Class group. Finally, a whole set of tab-delimited tables are produced for further downstream analyses or for drawing graphs in other programs, as required.

### Gene network

Venn diagrams are traditionally used to show the overlap between experiments, but this way of presenting limits the number of experiments that can be included. T-REx circumvents this problem and shows overlap between an unlimited number of experiments in a gene network using the Reingold-Tilford layout [12]. Furthermore, this gene network is exported as a table of nodes and edges that can be examined in a gene network analysis program such as Cytoscape [13].

### Gene set enrichment analysis

The results of the RNA-seq statistical pipeline describe and analyze the transcriptional behavior of genes/RNAs independently of the organism under study. To add organism-specific information to the analyzed data, a Gene Set Enrichment Analysis (GSEA) [14] is commonly used to unravel the bigger biological picture. The main issue in such an analysis is the availability of classification data for the specific organism such as Gene Ontology (GO) and metabolic pathways (KEGG). To allow for easy GSEA we (re-)annotated all proteins of all publically available bacterial complete genomes for GO, InterPro, KEGG, MetaCyc, PFAM domains, Superfamily and Gene3D. We implemented a webserver allowing to use this classification data for GSEA (http://genome2d.molgenrug.nl/index.php/functional-analysis-go-ipr).

**Figure 1:**
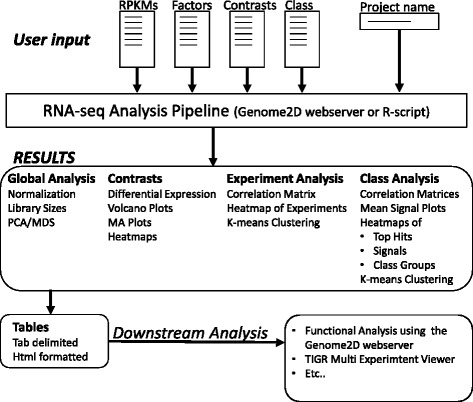
Flow chart of the RNA-seq analysis pipeline. User input consists of the four data files defined in Table 1 and a project name. Parameters such as thresholds, *p*-value cutoffs and k-means settings are predefined or will be estimated by the analysis pipeline

The workflow of T-REx is presented in Fig. 1. The analysis pipeline has been written in R [15] and is freely available on request. For reasons of user-friendliness, we offer a web server for uploading of the data files. The results of the analyses can be mined on a web-browser or downloaded as a zip-file (containing all html files, images and data files) for later use. Depending on server demand, a full analysis takes around 1 min.

## Results and discussion

The performance of the RNA-seq data analysis pipeline was assessed using the RNA-seq expression dataset (DatasetS1) of Brinsmade and coworkers [16]. These authors performed RNA-seq to study the effect of three separate single amino acid changes in the global transcription factor for nitrogen metabolism CodY of *Bacillus subtilis* 168. The format of DatasetS1 could be directly used as an input for our RNA-seq analysis pipeline. A Factors file was created to define strains and replicates, as explained in Table 1a. To find differential gene expression, all three CodY mutants were compared to the wild-type, as defined by the Contrasts file (Table 1B). Apart from the Data table, a Factors file and a Contrasts file, a Class file was made containing information on the CodY regulon and two other interesting regulons: those of CcpA [17, 18] and ArgR [19] (Table 1C). The four files were used as inputs for the webserver and over 40 html pages were retrieved that refer to 104 graphs, 45 tables and one html overview table (see Additional file 3).

### Global analysis

**Figure 2:**
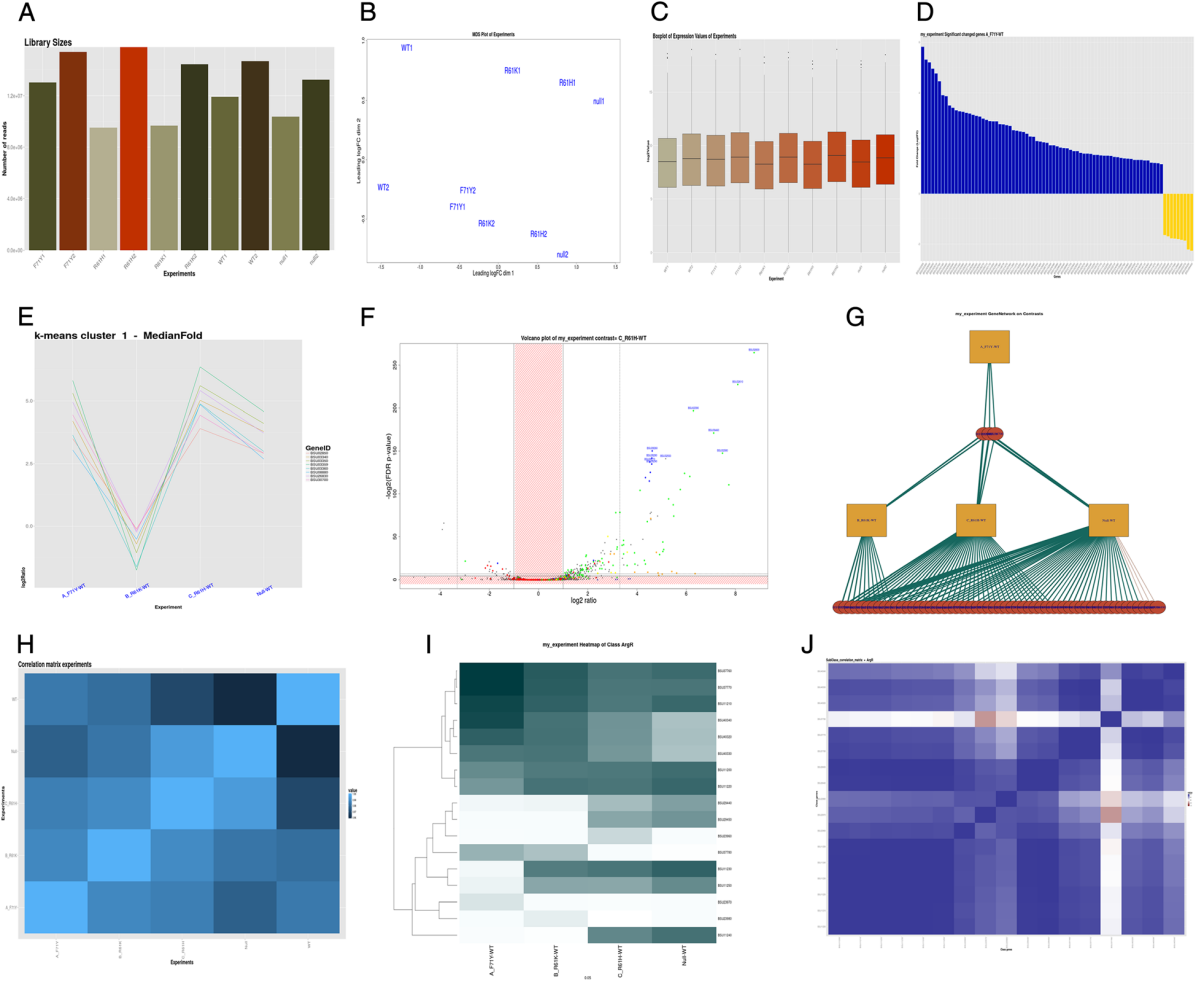
An illustration of images obtained by T-REx after analysis of the CodY dataset of Brinsmade et al.. **a**) Library sizes, **b**) Box plots of signals in each sample, **c**) MDS plot, **d**) Bar graph of up- and down-regulated genes, **e**) One of the k-means clusters, **f**) One of the Volcano plots, **g**) Network of genes and experiments, **h**) Correlation matrix of experiments, **i**) Heatmaps of Class genes to experiments, **j**) Correlation matrix of Class genes to Class genes. For the same images in high-resolution, see Additional file 4: Figure S2A – S2J. A tutorial for interpretation of T-REx results is given on the T-REx webserver

The results of the global analysis (Fig. 2) showed that the library sizes and signal distributions were comparable for all samples. The biological replicates of the CodY mutant F71Y showed a higher correlation than those of the other 2 mutants, R61K and R61H, but all samples were well distributed upon examination of the Multi-Dimensional Scaling (MDS) plot (Fig. 2).

### Contrasts analysis

Similar to what was observed by Brinsmade et al., more genes were up- than down-regulated in the CodY knock-out (Null mutant) versus wild-type comparison at a *p*-value <0.05 and a fold-change >3. The absolute number of differentially expressed genes was higher in our analysis as we used a Fold Change cutoff of 2 instead of 3 (Table 2D, *p*-value < 0.05 and fold-change >2). Genes of the known CodY regulon were colored green (see the Class file of the example data) allowing to easily spot them in the MA- and volcano plots. Thus, it is immediately clear that more genes than only those of the CodY regulon are differentially expressed, as was also pinpointed by Brinsmade et al.. The blue dots in the upper-right quadrant of the volcano plots indicate that several genes of the CodY regulon are also part of the CcpA regulon, an observation that was also reported in Brinsmade et al.. The heatmap (Additional file 4: Figure S2I) of differentially expressed genes in all CodY mutants showed interesting groupings of genes, which might be of importance for biological interpretation and further analysis.

**Table 2:** Overview table of the analysis of differential gene expression

Contrast	Total number of genes	Up-regulated	Down-regulated
A_F71Y-WT	4176	72	9
B_R61K-WT	4176	126	15
C_R61H-WT	4176	219	25
Null-WT	4176	282 (196/212)	47 (27/29)

The numbers of up- and down-regulated genes were determined using default cutoffs *p*-value ≤ 0.05 and fold-change ≥ 2. Within brackets *p*-value ≤ 0.05 and fold change ≥ 3 as was mentioned in Brinsmade et al. and our pipeline, respectively

### Experiment analysis

When analyzing all CodY mutants (targets) to the wild type (control), the number of up and down regulated genes identified by T-REx was comparable to that reported in Brinsmade et al. (Table 2). The ‘Correlation Matrix of Experiments’ figure (Fig. 2 and Additional file 4: Figure S2H) is in agreement with a gradual increase in the number of differentially expressed genes in the mutants, in the order F71Y, R61K, R61H and Null, as was also concluded in Brinsmade et al.. Automated k-means clustering of differentially expressed genes in the various mutants (see Additional file 5: Figure S3) also shows a gradient of gene expression, suggesting that not only the number of differentially expressed genes differs between the mutants, but also their gene expression levels. By a gradual increase in expression of certain genes in the various mutants, the ratios (mutant versus WT) pass a preset threshold value, which might explain why the number of differentially expressed genes increases.

### Class analysis

The T-REx pipeline performs an in-depth analysis on classes pre-defined by the user. Here we defined five classes; the regulons CodY, CcpA, ArgR, MalR and a Class of genes that are under control of both CodY and CcpA. The heatmap (Class genes to experiments) and the correlation matrix of Class genes showed that some members of the known CodY regulon do not have a good correlation over the experiments. Brinsmade et al. excluded several genes from their analyses because of their complex gene expression patterns. To study this phenomenon we added these genes in a separate Class ‘Complex’ and colored them orange. The volcano plots showed that a subset of 7 of these genes appears as differentially regulated compared to the wild type strain in three of the four contrasts. The probability of their differential expression is close to the *p*-value threshold (in this case 0.05) in the mutant R61K. The heatmap of signals of Class ‘Complex’ (see Additional file 6: Figure S4A) showed that these 7 genes have a gene expression pattern that is different from the genes of the CodY regulon (see Additional file 6: Figure S4B).

## Conclusion

The parameter-free RNA-seq analysis pipeline T-REx is a fast, easy to use and comprehensive way to perform statistical analysis of gene expression data derived from RNA-seq data. Typical graphics and tables are automatically generated, which enables a direct overview of the biological relevance of the data, obviating laborious combining and complex filtering operations of data. Furthermore, T-REx produces data tables for (optional) downstream processing. The case study presented in this article compared the analyses performed by Brinsmade et al. and T-REx. The outcome shows that T-REx can quickly and fully automatically perform statistical analyses on gene expression data derived from RNA-seq. It reproduced the results of the original study without requiring additional statistical analyses. The T-REx pipeline is continuously updated and expanded to fully utilize the potential of RNA-seq gene expression data sets.

## Availability and requirements

- Project name: T-REx.
- Project home page: http://genome2d.molgenrug.nl.
- Operating system(s): Platform independent.
- Programming language: Perl, R.
- License: This website is free and open to all users and there is no login requirement.

## Additional files

Additional file 1:
**Archive file containing all T-REx output files.** (R 38 kb) Additional file 2:
**Figures S2A - S2J; high resolution images of Figure** **2** **.** (R 13 kb) Additional file 3:
**Figure S3; k-means clustering of differentially expressed genes in the mutants.** (ZIP 31925 kb) Additional file 4:
**Figures S4A and S4B; signal heatmaps of Class ‘Complex’ and Class ‘CodY’, respectively.** (ZIP 1103 kb) Additional file 5:
**T-REx main R-script.** (PNG 173 kb) Additional file 6:
**T-REx R functions.** (ZIP 179 kb)
