# Supplementary figures and images for: T-REx: Transcriptome analysis webserver for RNA-seq Expression data (part 2 of 2)
Source: BMC Genomics. 2015 Sep 3;16(1):663. doi: 10.1186/s12864-015-1834-4 (PMC4558784; doi:10.1186/s12864-015-1834-4)

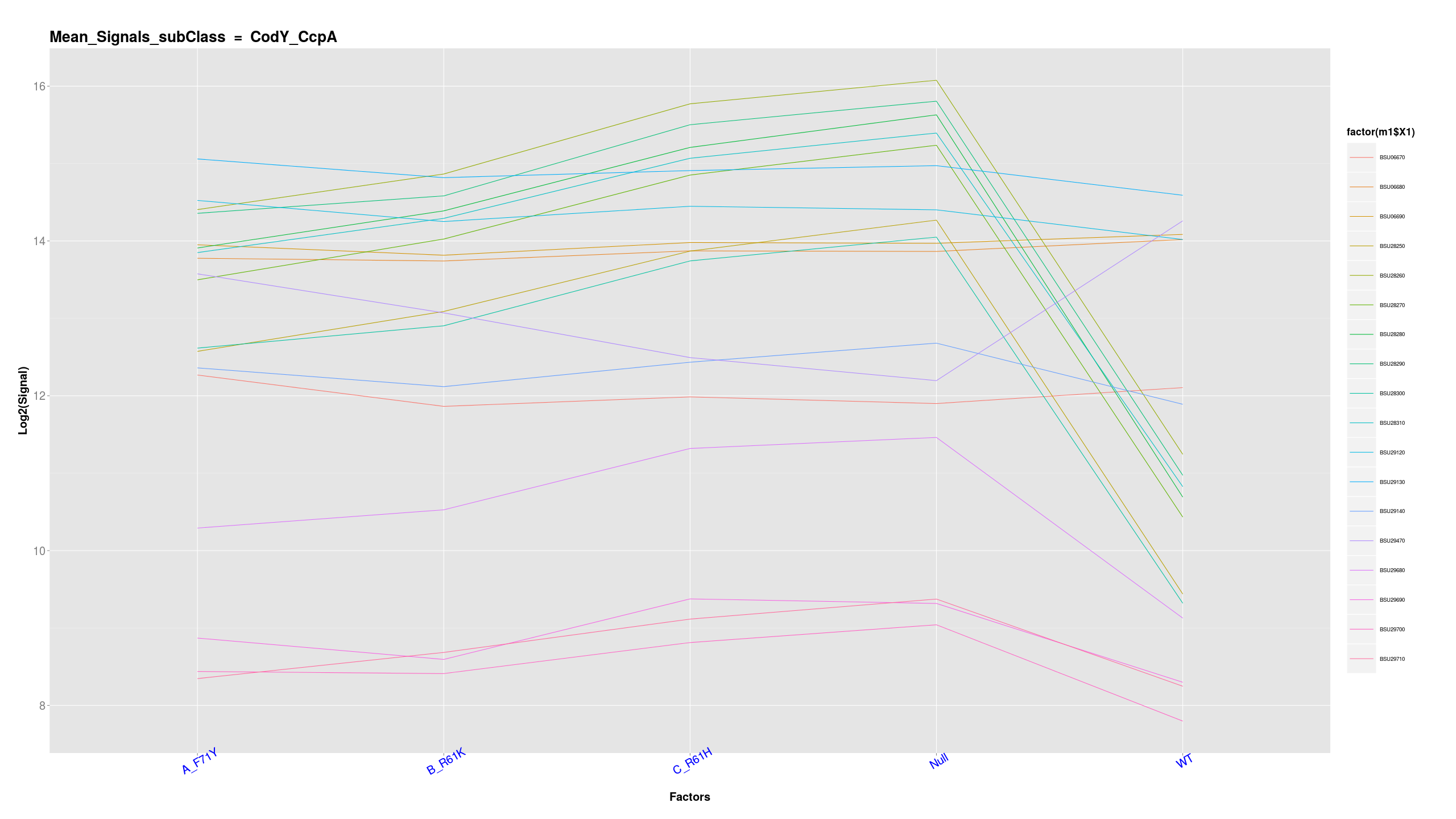

Supplement: Additional file 3: — Figure S3; k-means clustering of differentially expressed genes in the mutants. (ZIP 31925 kb) [file 12864_2015_1834_MOESM3_ESM.zip › Brinsmade.Mean_Signals_subClass.CodY_CcpA.png]

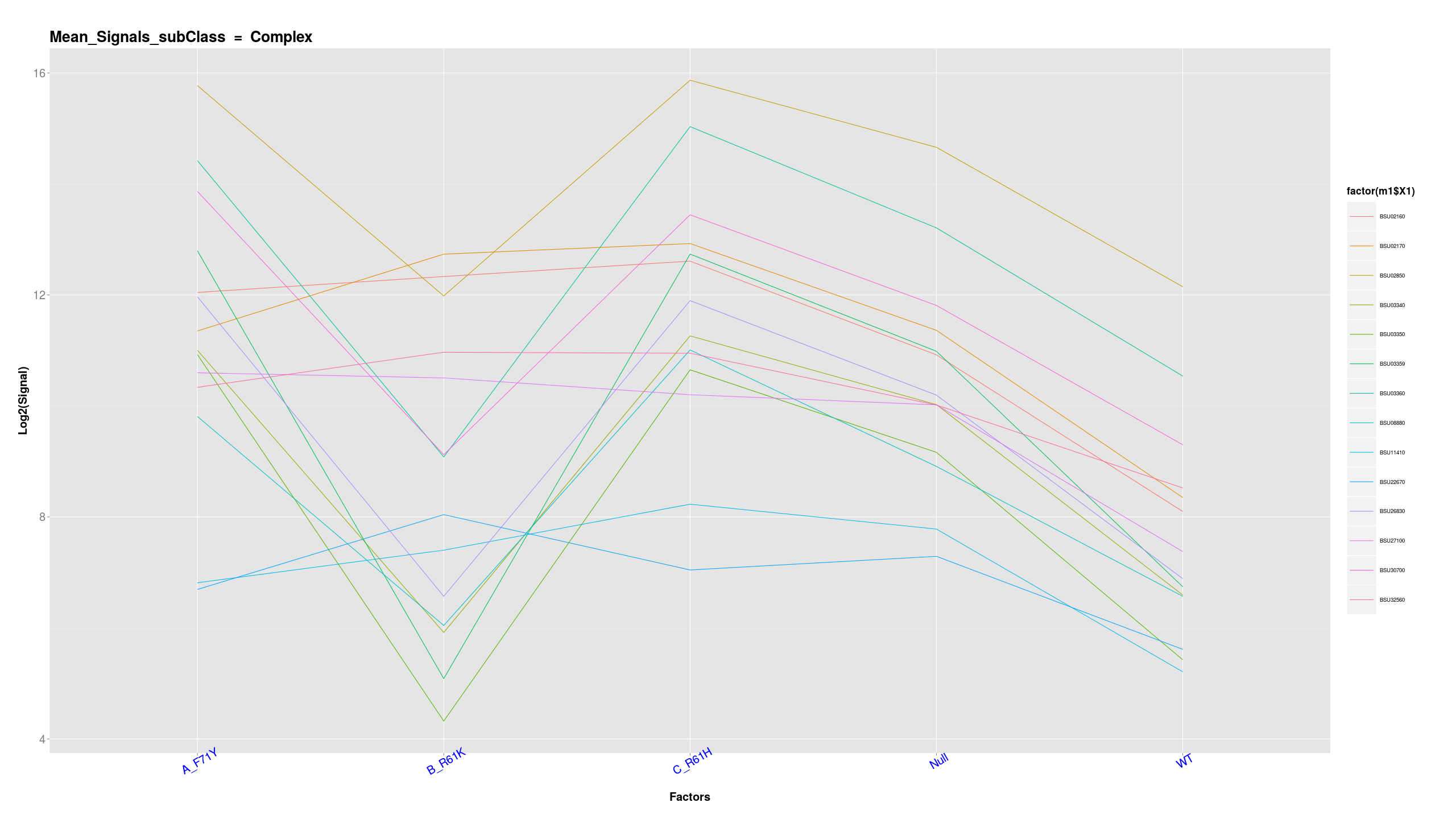

Supplement: Additional file 3: — Figure S3; k-means clustering of differentially expressed genes in the mutants. (ZIP 31925 kb) [file 12864_2015_1834_MOESM3_ESM.zip › Brinsmade.Mean_Signals_subClass.Complex.png]

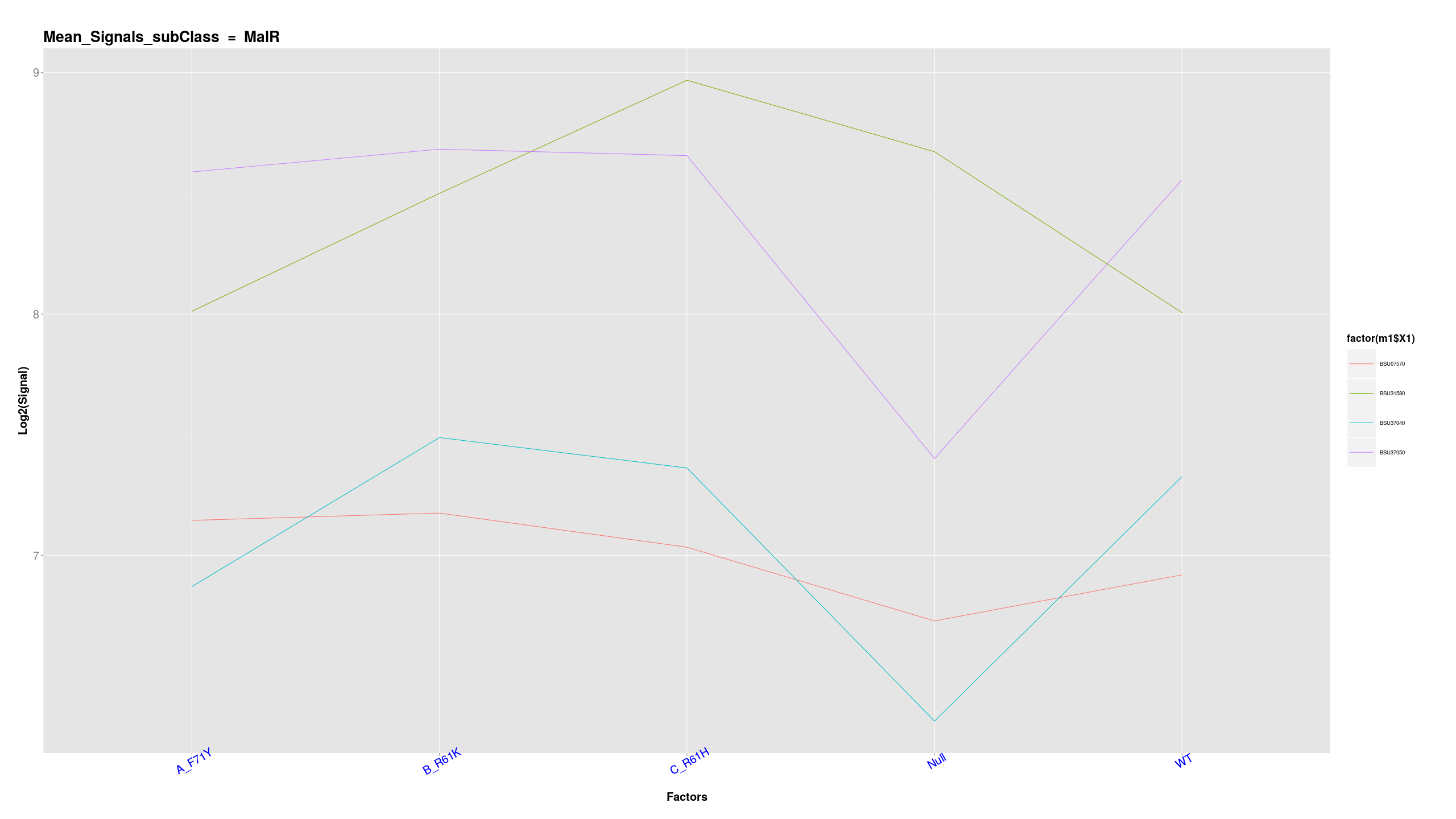

Supplement: Additional file 3: — Figure S3; k-means clustering of differentially expressed genes in the mutants. (ZIP 31925 kb) [file 12864_2015_1834_MOESM3_ESM.zip › Brinsmade.Mean_Signals_subClass.MalR.png]

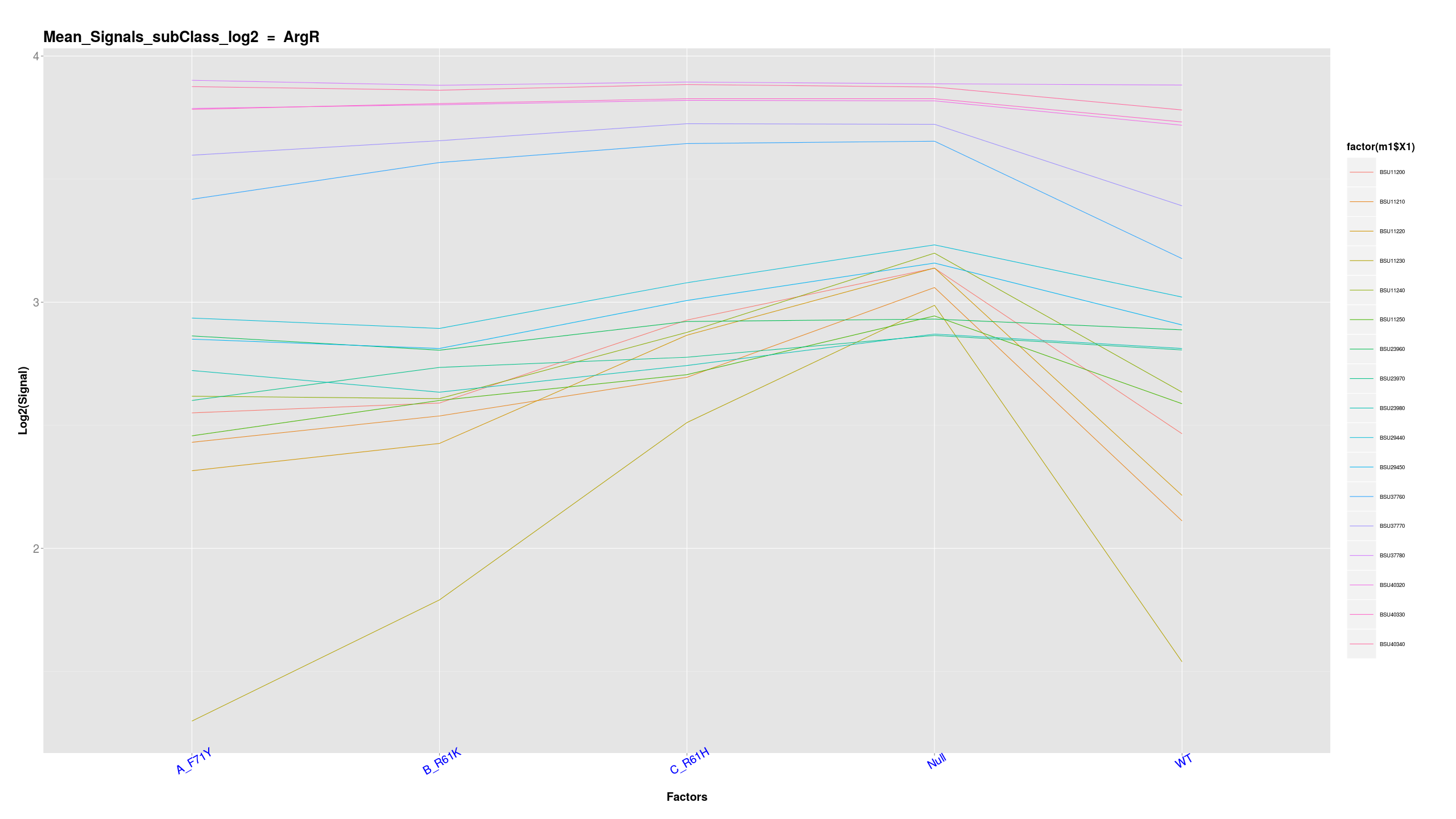

Supplement: Additional file 3: — Figure S3; k-means clustering of differentially expressed genes in the mutants. (ZIP 31925 kb) [file 12864_2015_1834_MOESM3_ESM.zip › Brinsmade.Mean_Signals_subClass_log2.ArgR.png]

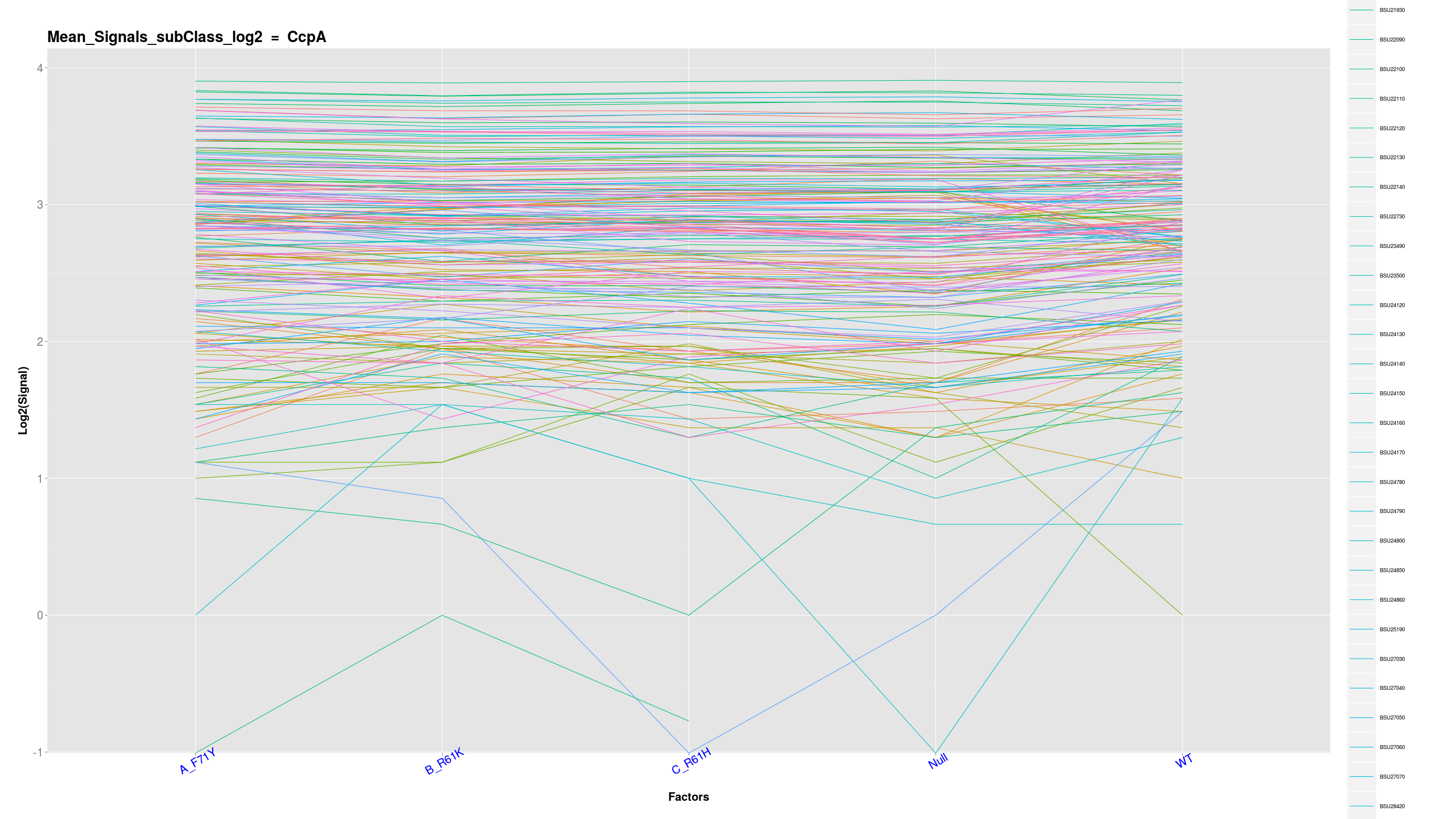

Supplement: Additional file 3: — Figure S3; k-means clustering of differentially expressed genes in the mutants. (ZIP 31925 kb) [file 12864_2015_1834_MOESM3_ESM.zip › Brinsmade.Mean_Signals_subClass_log2.CcpA.png]

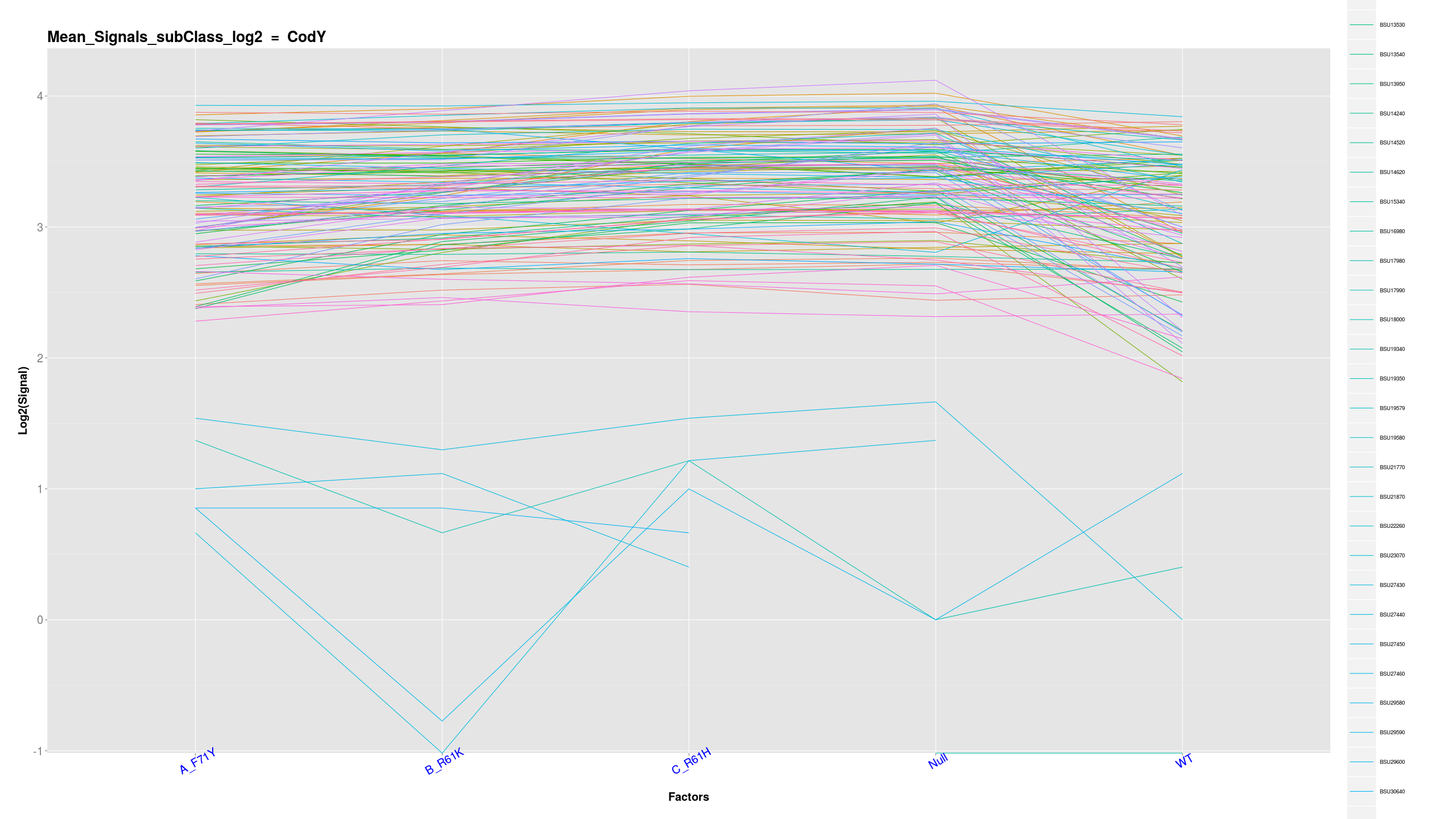

Supplement: Additional file 3: — Figure S3; k-means clustering of differentially expressed genes in the mutants. (ZIP 31925 kb) [file 12864_2015_1834_MOESM3_ESM.zip › Brinsmade.Mean_Signals_subClass_log2.CodY.png]

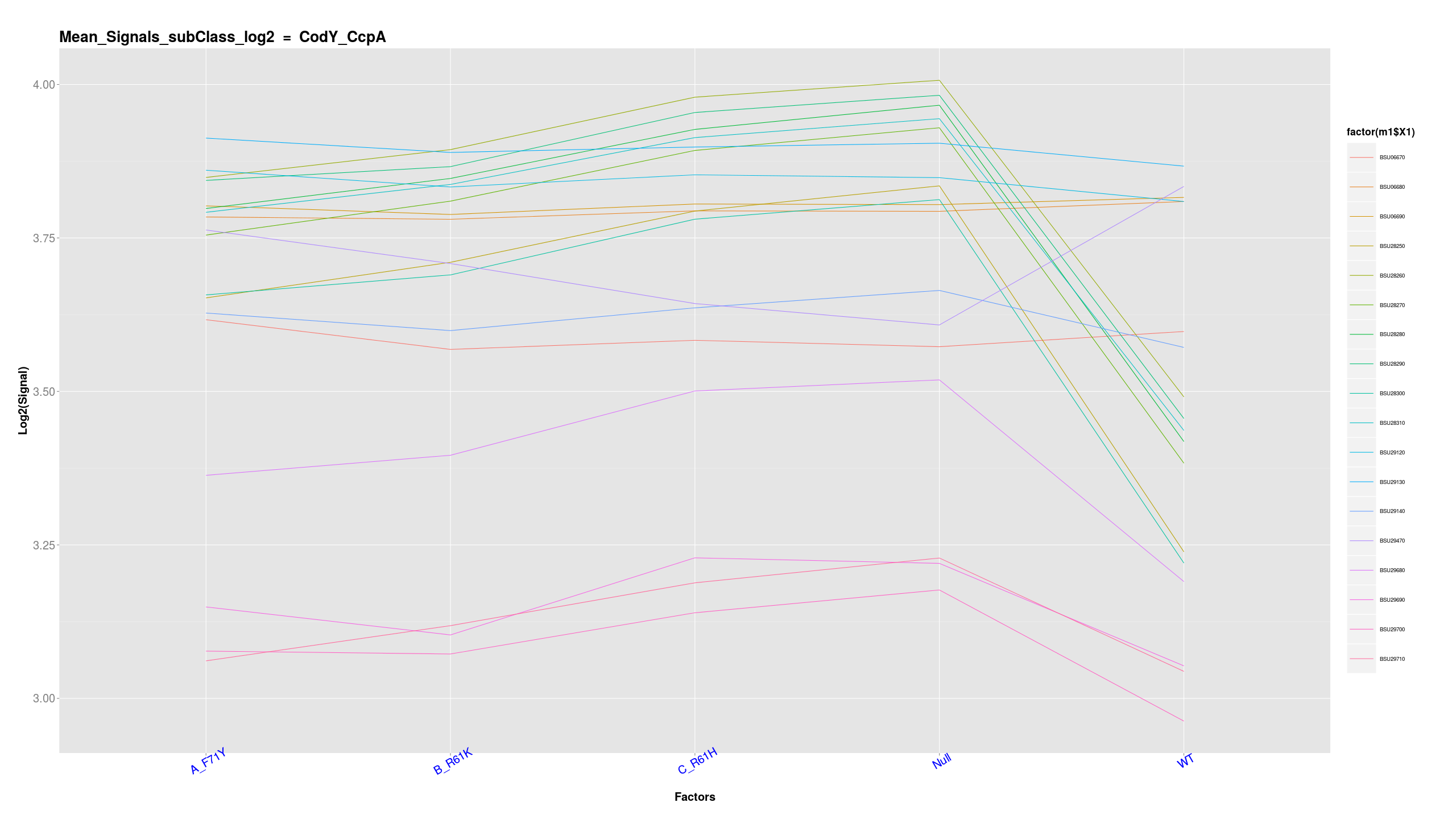

Supplement: Additional file 3: — Figure S3; k-means clustering of differentially expressed genes in the mutants. (ZIP 31925 kb) [file 12864_2015_1834_MOESM3_ESM.zip › Brinsmade.Mean_Signals_subClass_log2.CodY_CcpA.png]

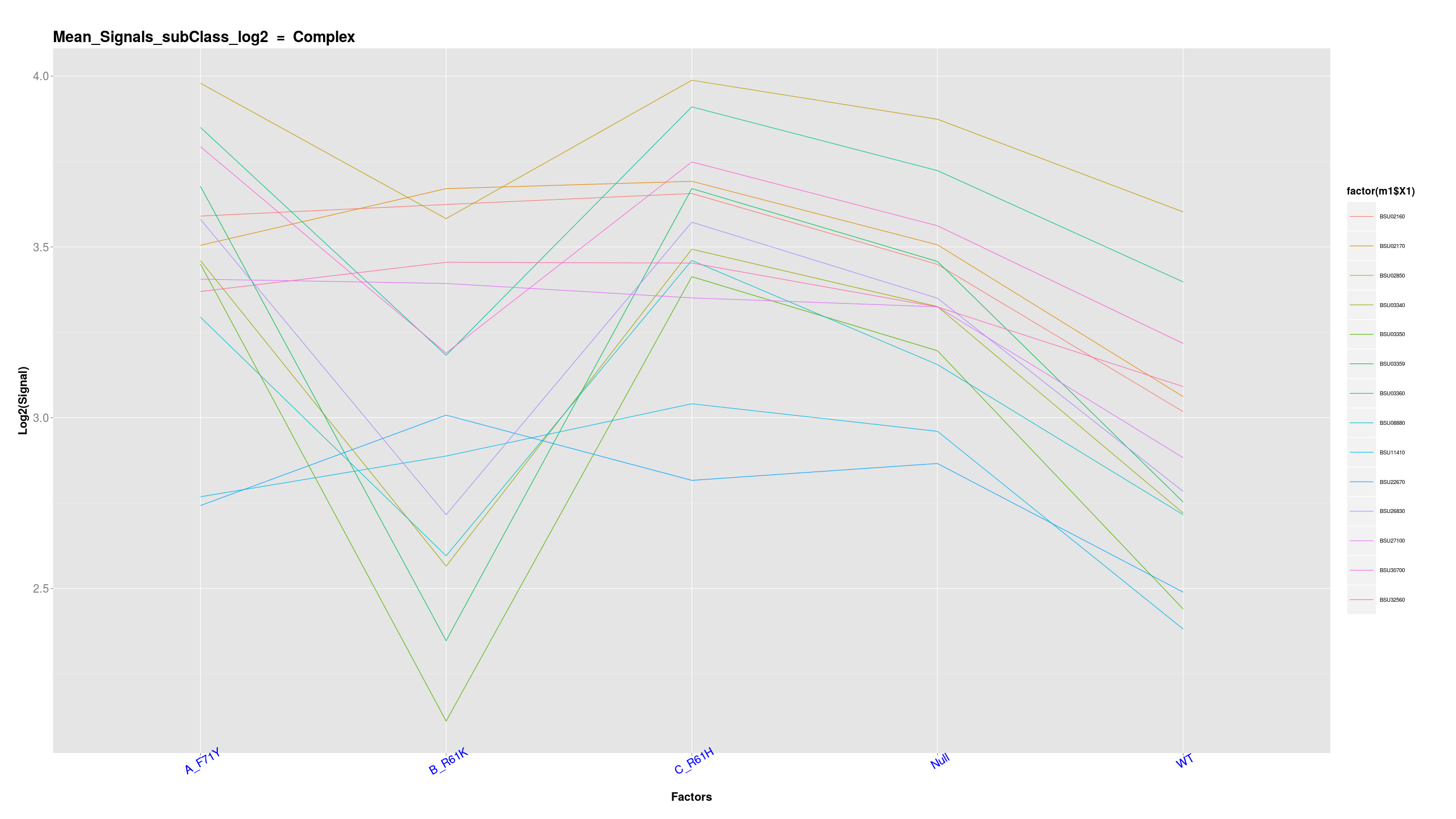

Supplement: Additional file 3: — Figure S3; k-means clustering of differentially expressed genes in the mutants. (ZIP 31925 kb) [file 12864_2015_1834_MOESM3_ESM.zip › Brinsmade.Mean_Signals_subClass_log2.Complex.png]

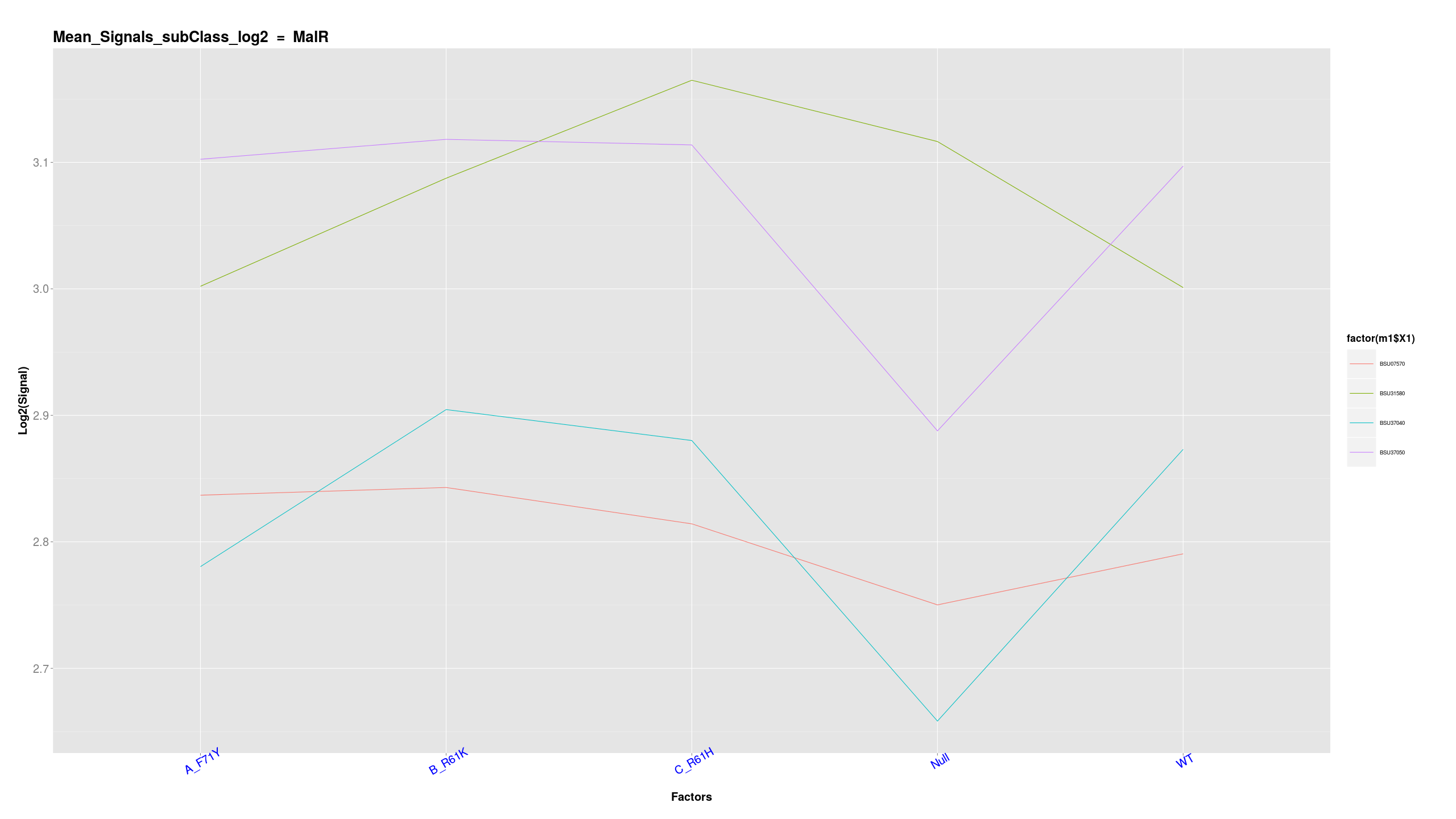

Supplement: Additional file 3: — Figure S3; k-means clustering of differentially expressed genes in the mutants. (ZIP 31925 kb) [file 12864_2015_1834_MOESM3_ESM.zip › Brinsmade.Mean_Signals_subClass_log2.MalR.png]

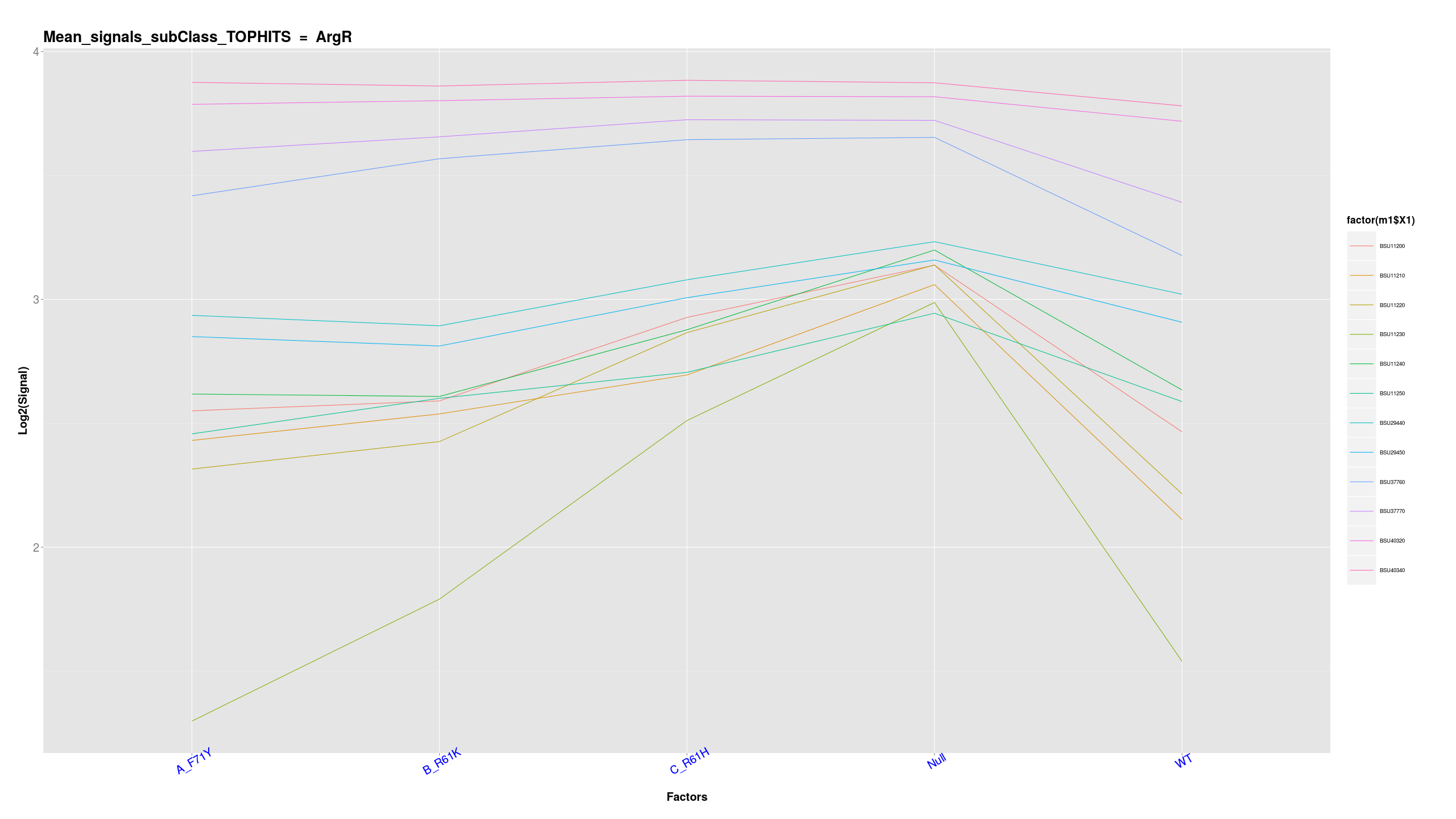

Supplement: Additional file 3: — Figure S3; k-means clustering of differentially expressed genes in the mutants. (ZIP 31925 kb) [file 12864_2015_1834_MOESM3_ESM.zip › Brinsmade.Mean_signals_subClass_TOPHITS.ArgR.png]

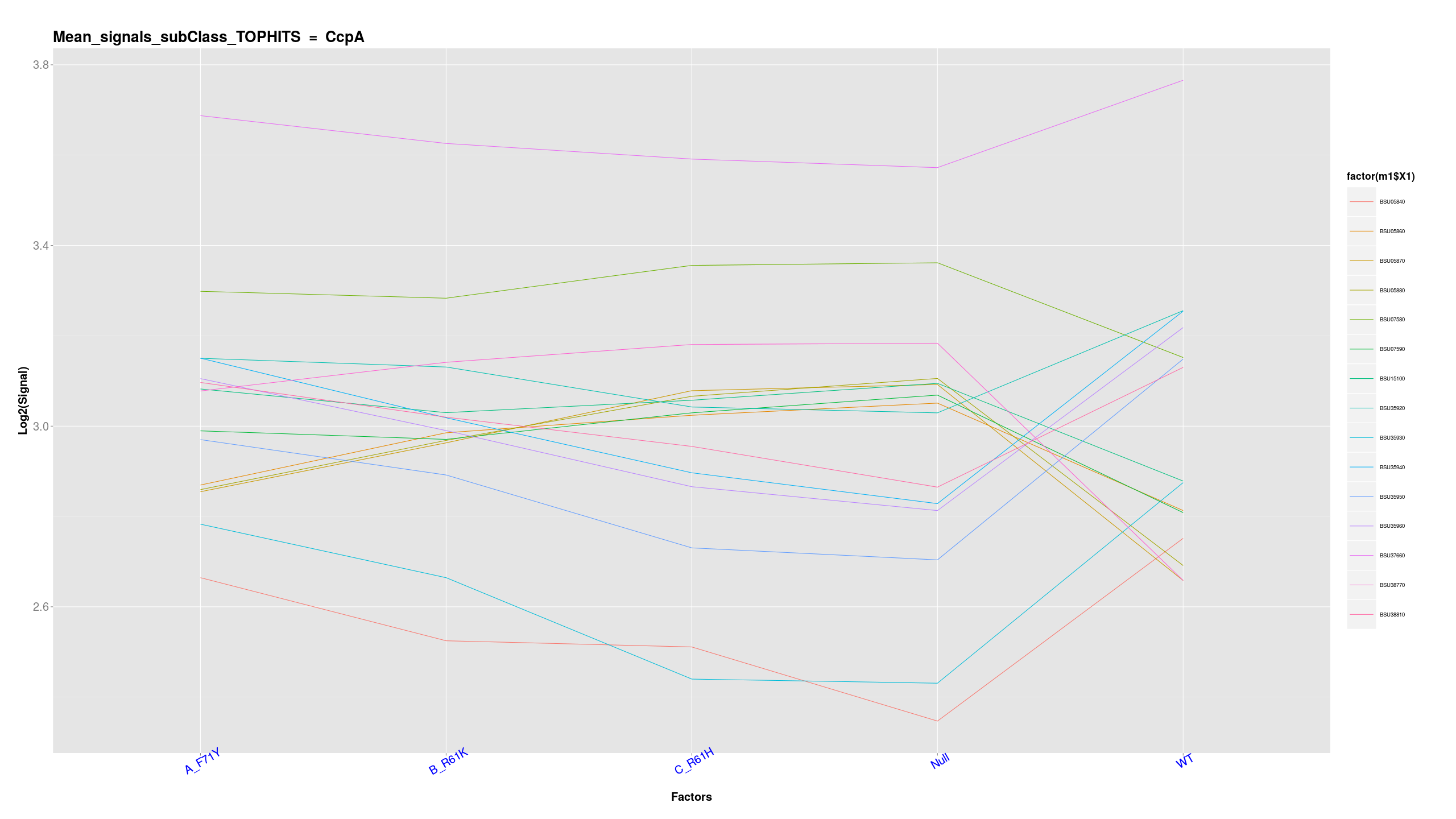

Supplement: Additional file 3: — Figure S3; k-means clustering of differentially expressed genes in the mutants. (ZIP 31925 kb) [file 12864_2015_1834_MOESM3_ESM.zip › Brinsmade.Mean_signals_subClass_TOPHITS.CcpA.png]

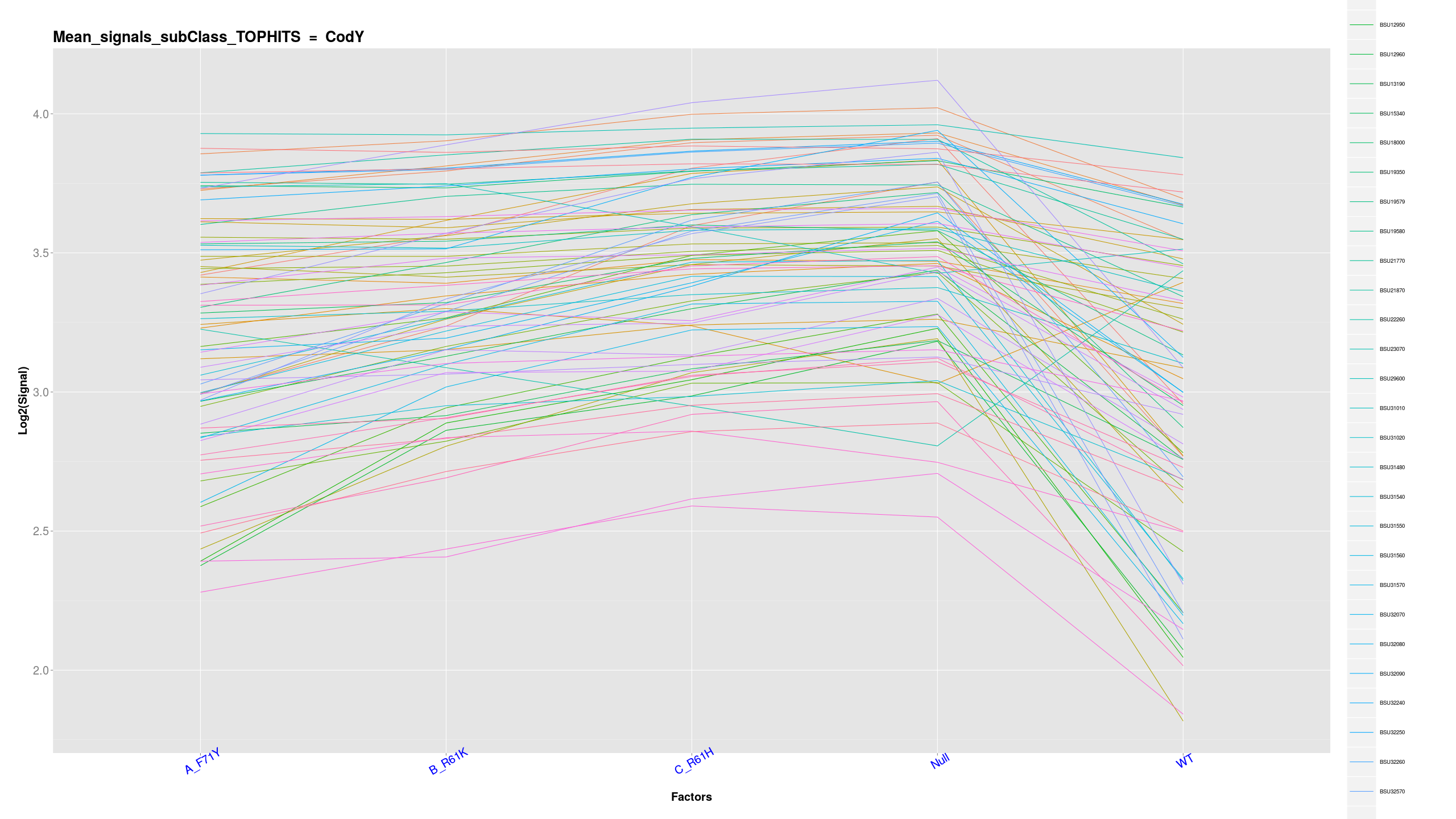

Supplement: Additional file 3: — Figure S3; k-means clustering of differentially expressed genes in the mutants. (ZIP 31925 kb) [file 12864_2015_1834_MOESM3_ESM.zip › Brinsmade.Mean_signals_subClass_TOPHITS.CodY.png]

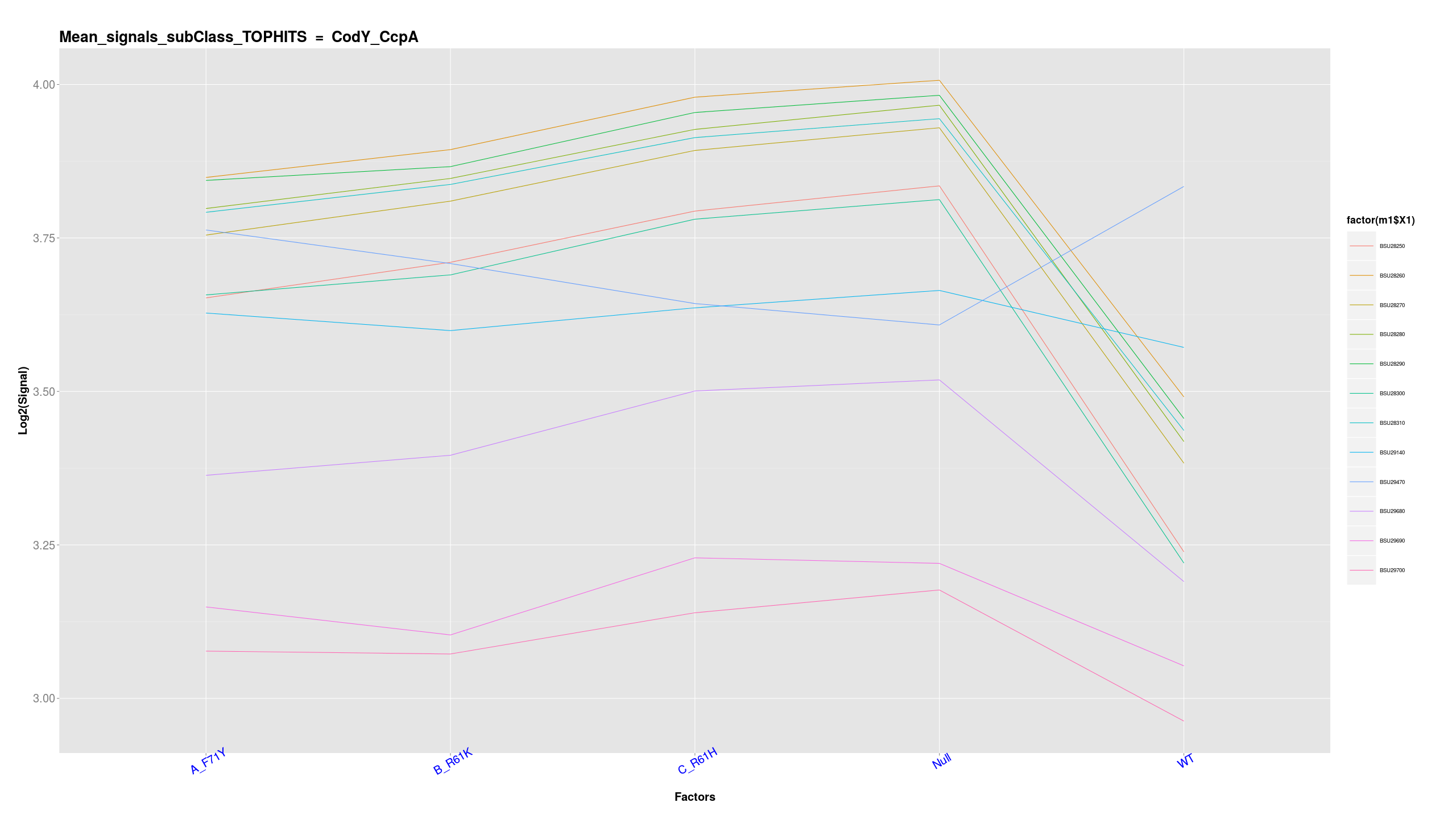

Supplement: Additional file 3: — Figure S3; k-means clustering of differentially expressed genes in the mutants. (ZIP 31925 kb) [file 12864_2015_1834_MOESM3_ESM.zip › Brinsmade.Mean_signals_subClass_TOPHITS.CodY_CcpA.png]

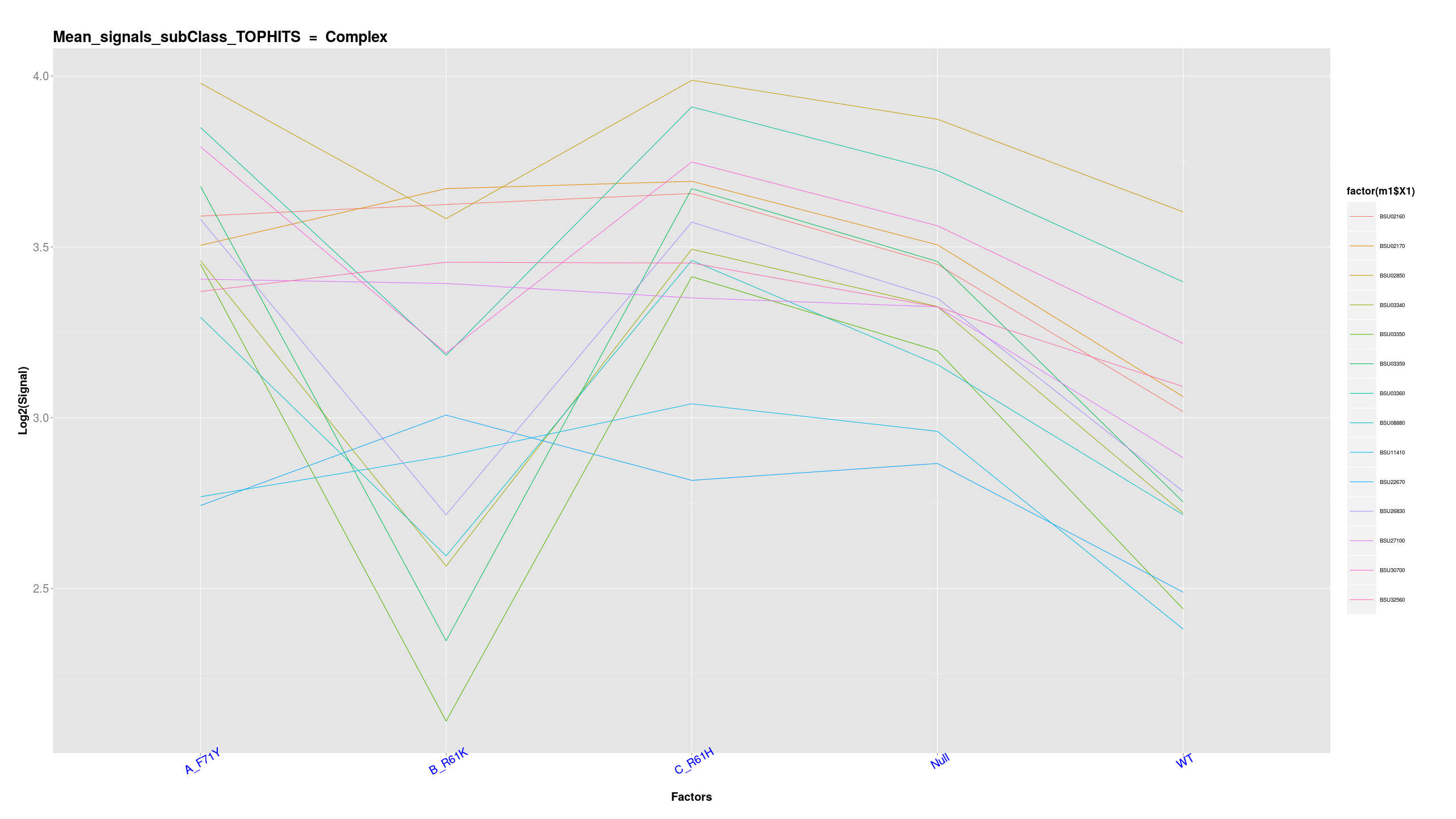

Supplement: Additional file 3: — Figure S3; k-means clustering of differentially expressed genes in the mutants. (ZIP 31925 kb) [file 12864_2015_1834_MOESM3_ESM.zip › Brinsmade.Mean_signals_subClass_TOPHITS.Complex.png]

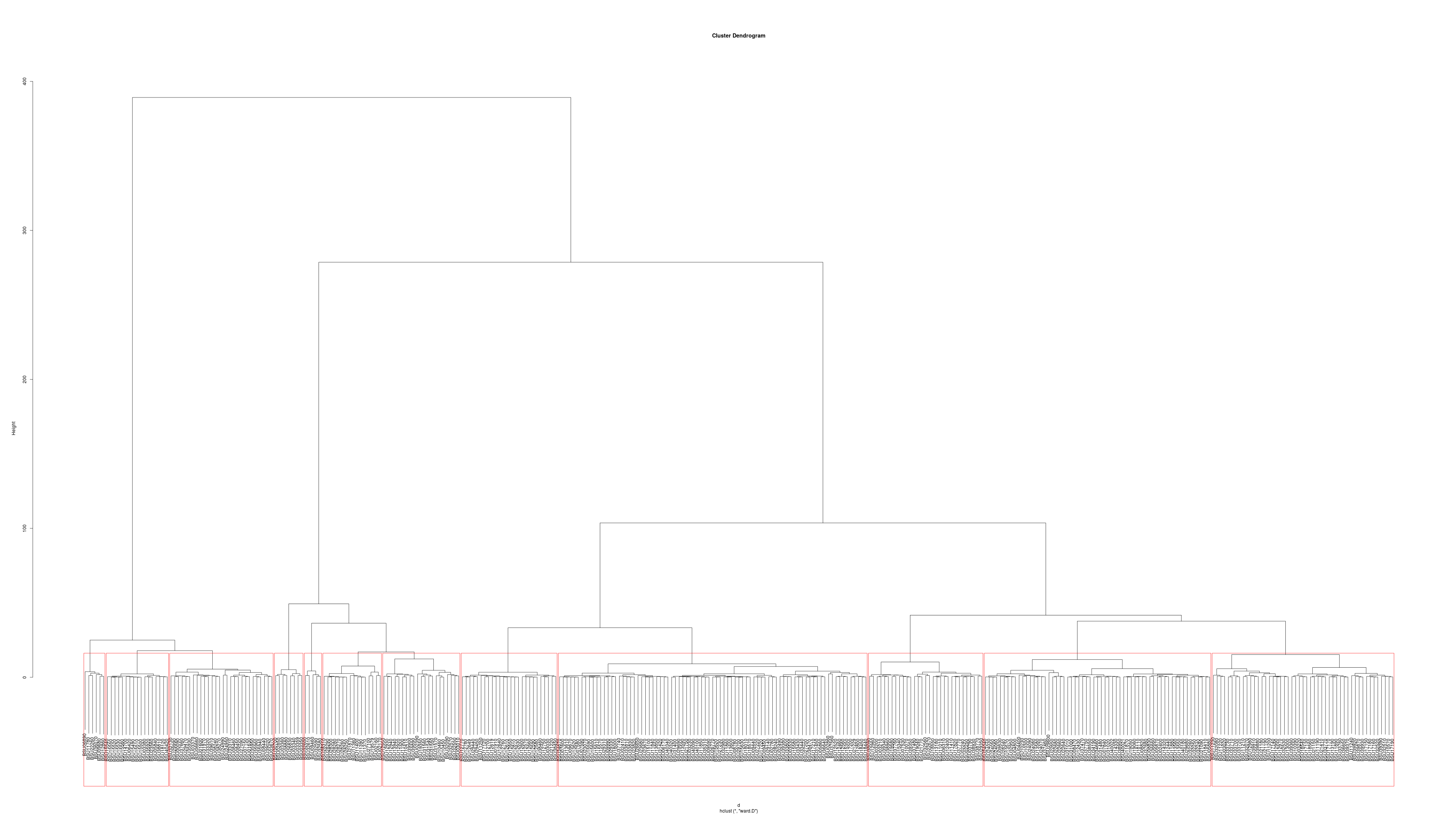

Supplement: Additional file 3: — Figure S3; k-means clustering of differentially expressed genes in the mutants. (ZIP 31925 kb) [file 12864_2015_1834_MOESM3_ESM.zip › Brinsmade.MedianFold.kmeans_Dendrogram.png]

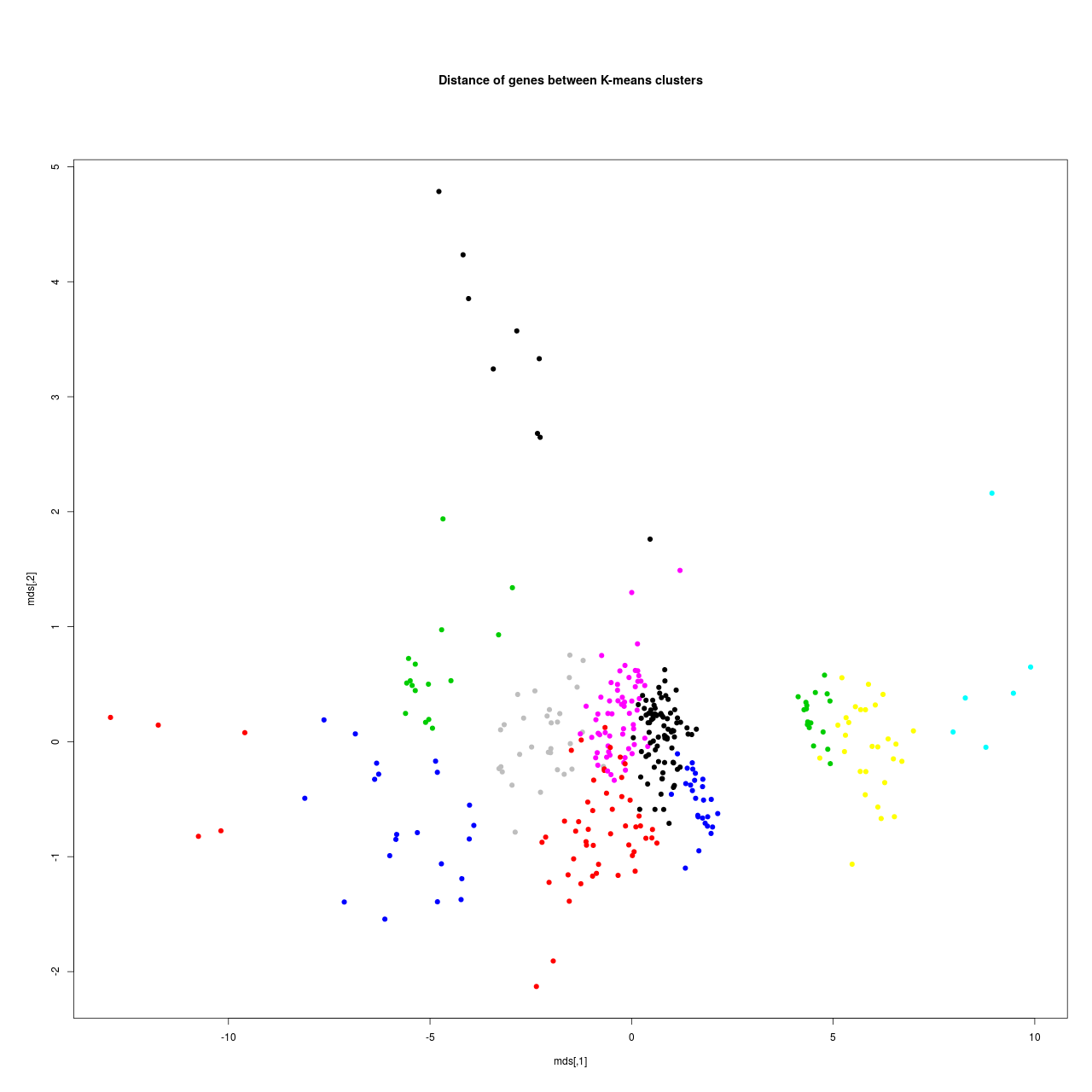

Supplement: Additional file 3: — Figure S3; k-means clustering of differentially expressed genes in the mutants. (ZIP 31925 kb) [file 12864_2015_1834_MOESM3_ESM.zip › Brinsmade.MedianFold.kmeans_MDS.png]

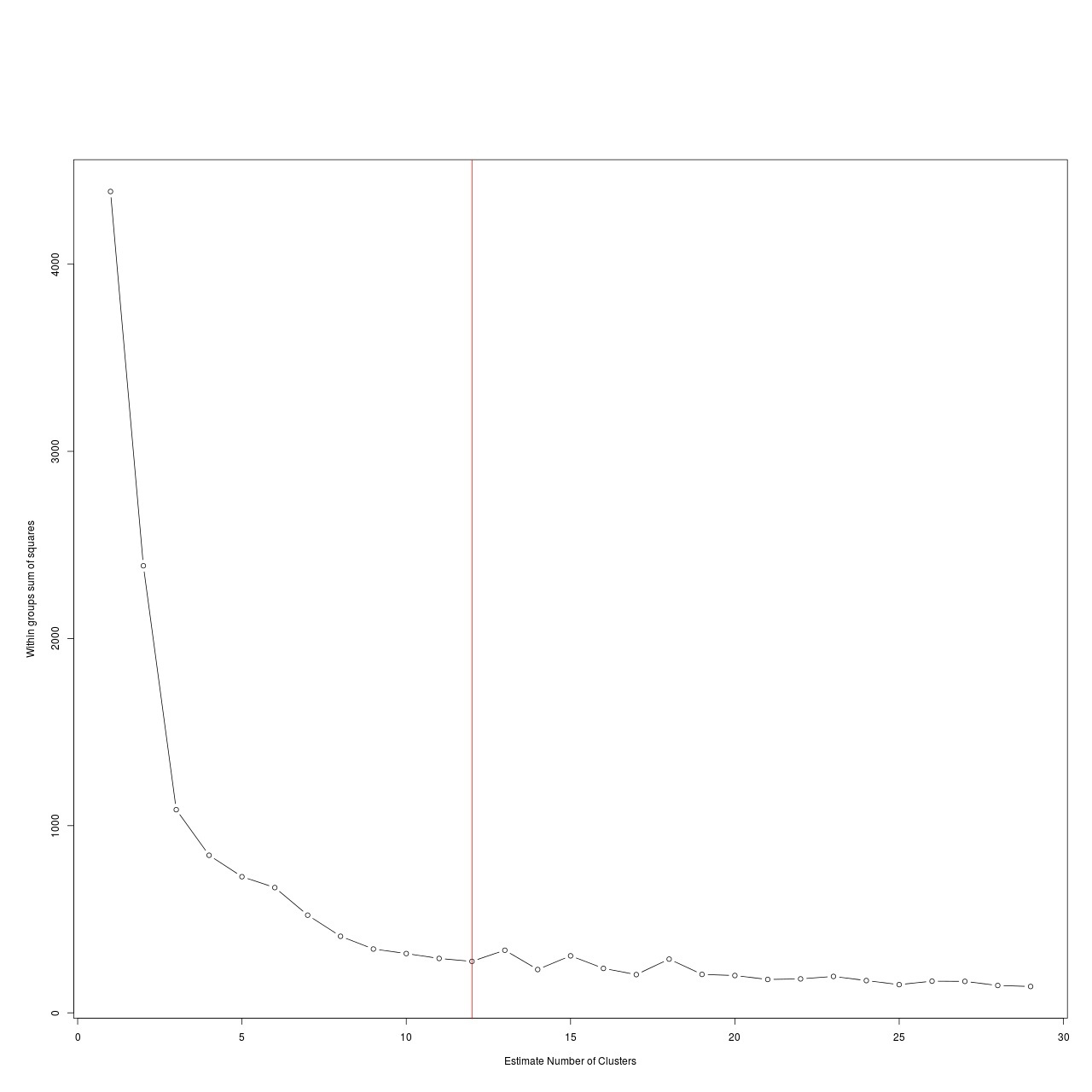

Supplement: Additional file 3: — Figure S3; k-means clustering of differentially expressed genes in the mutants. (ZIP 31925 kb) [file 12864_2015_1834_MOESM3_ESM.zip › Brinsmade.MedianFold.kmeans_estimates.png]

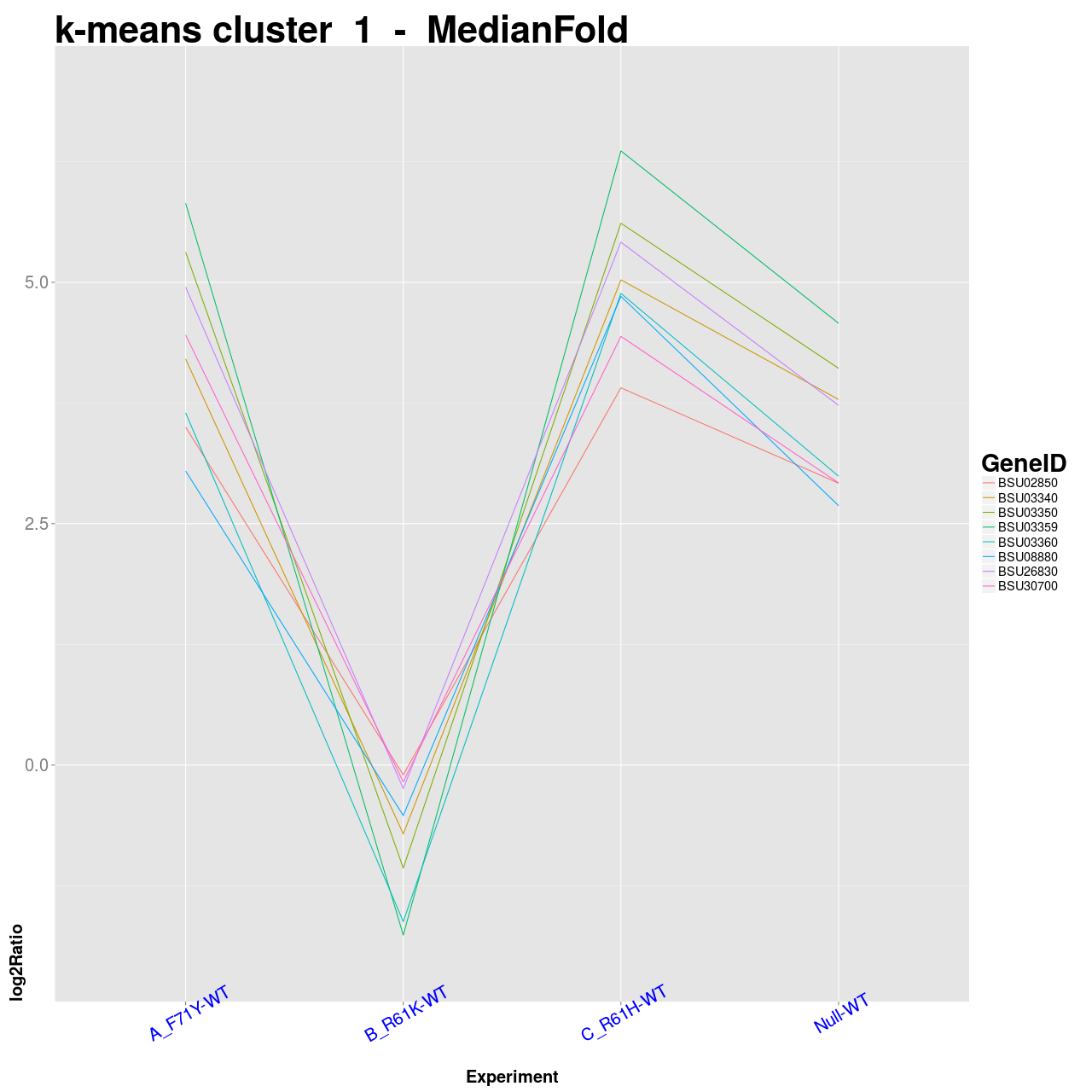

Supplement: Additional file 3: — Figure S3; k-means clustering of differentially expressed genes in the mutants. (ZIP 31925 kb) [file 12864_2015_1834_MOESM3_ESM.zip › Brinsmade.MedianFold.kmeans_plot_cluster.1.png]

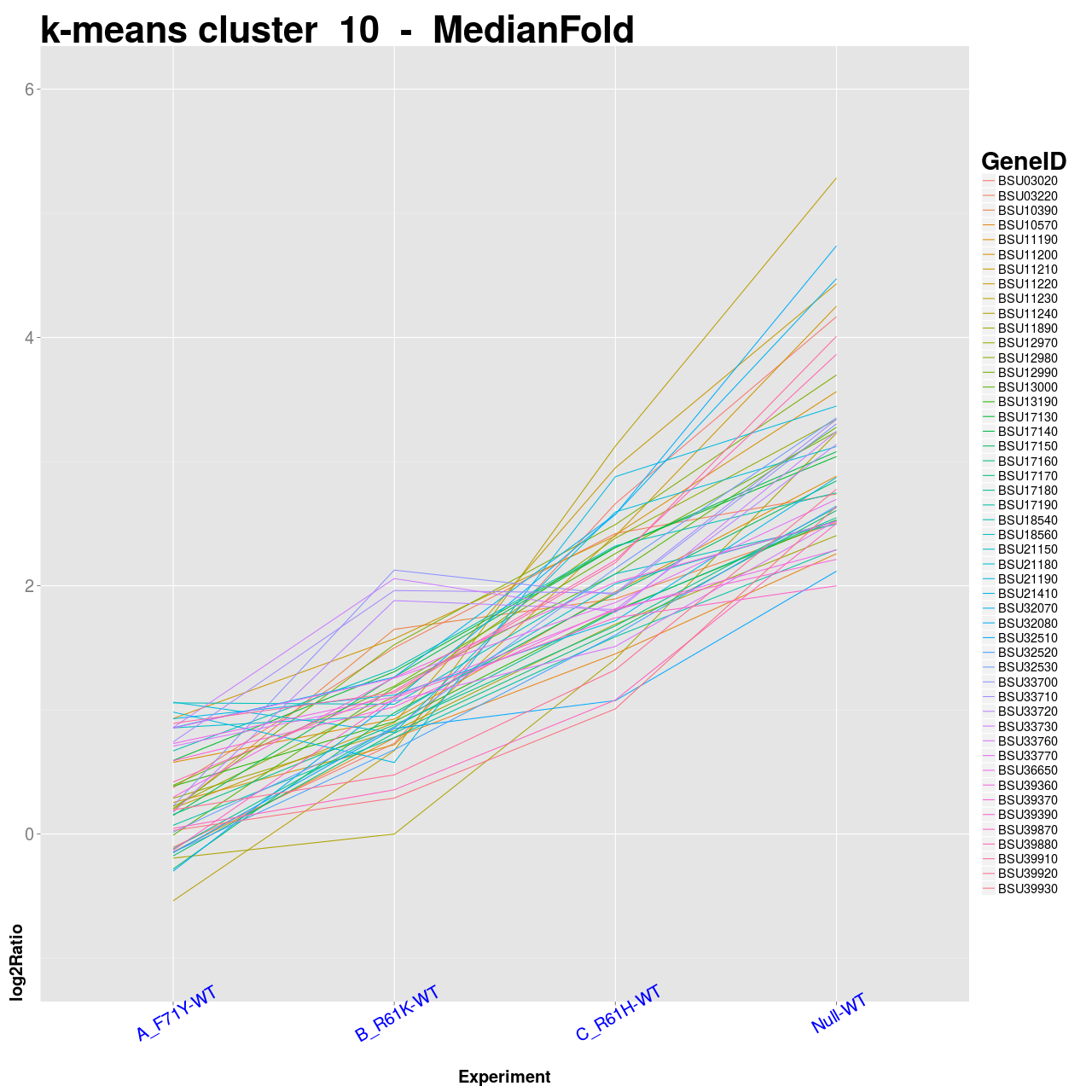

Supplement: Additional file 3: — Figure S3; k-means clustering of differentially expressed genes in the mutants. (ZIP 31925 kb) [file 12864_2015_1834_MOESM3_ESM.zip › Brinsmade.MedianFold.kmeans_plot_cluster.10.png]

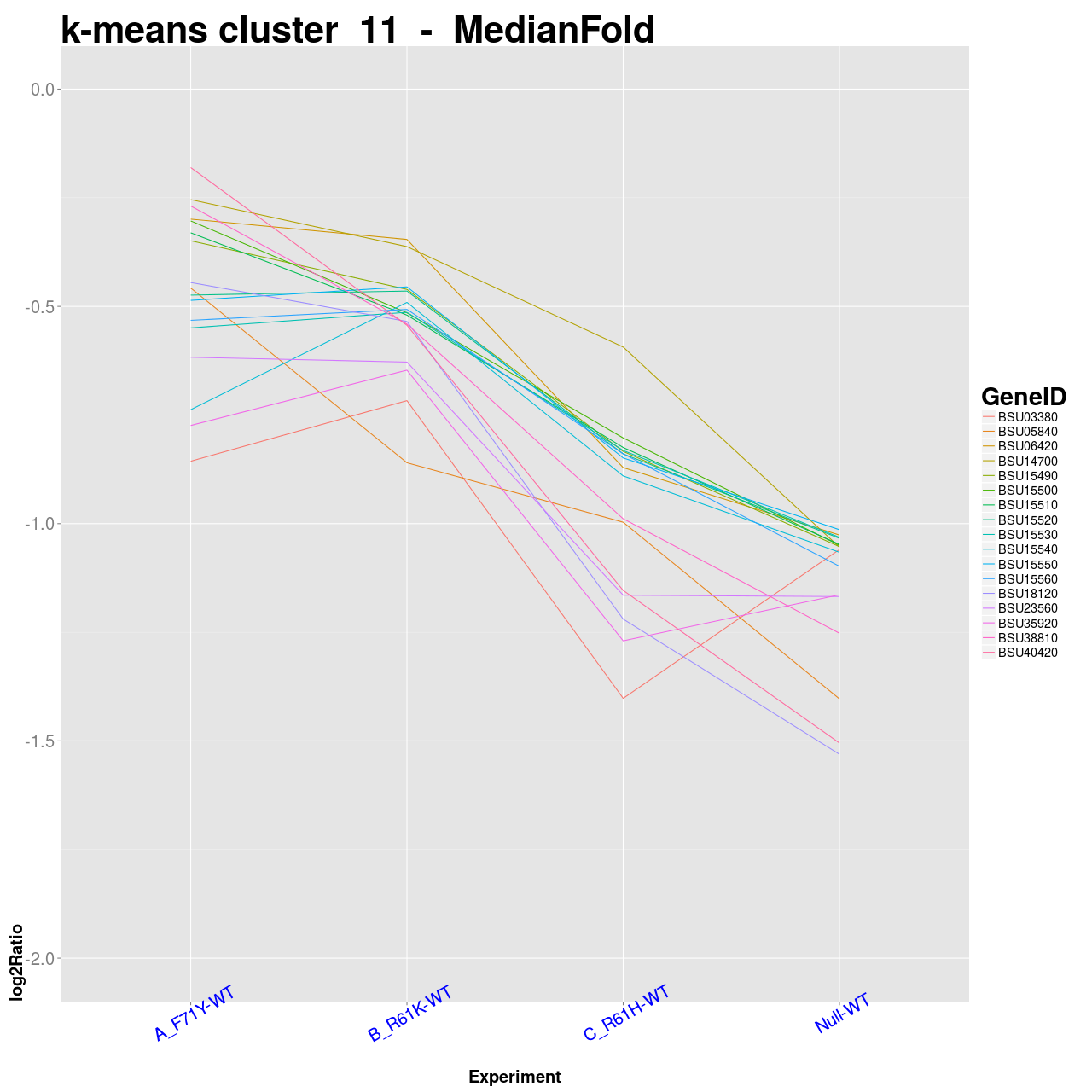

Supplement: Additional file 3: — Figure S3; k-means clustering of differentially expressed genes in the mutants. (ZIP 31925 kb) [file 12864_2015_1834_MOESM3_ESM.zip › Brinsmade.MedianFold.kmeans_plot_cluster.11.png]

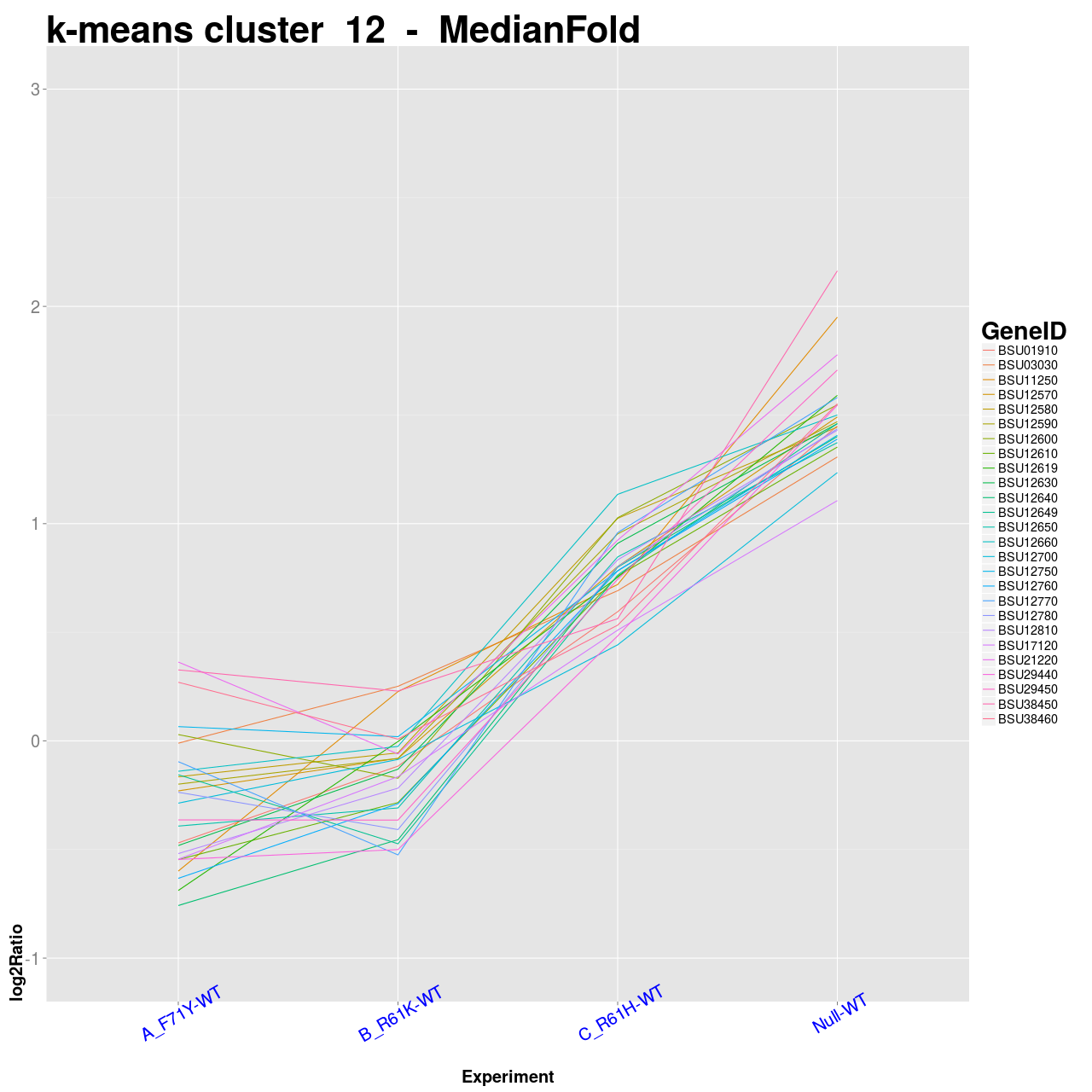

Supplement: Additional file 3: — Figure S3; k-means clustering of differentially expressed genes in the mutants. (ZIP 31925 kb) [file 12864_2015_1834_MOESM3_ESM.zip › Brinsmade.MedianFold.kmeans_plot_cluster.12.png]

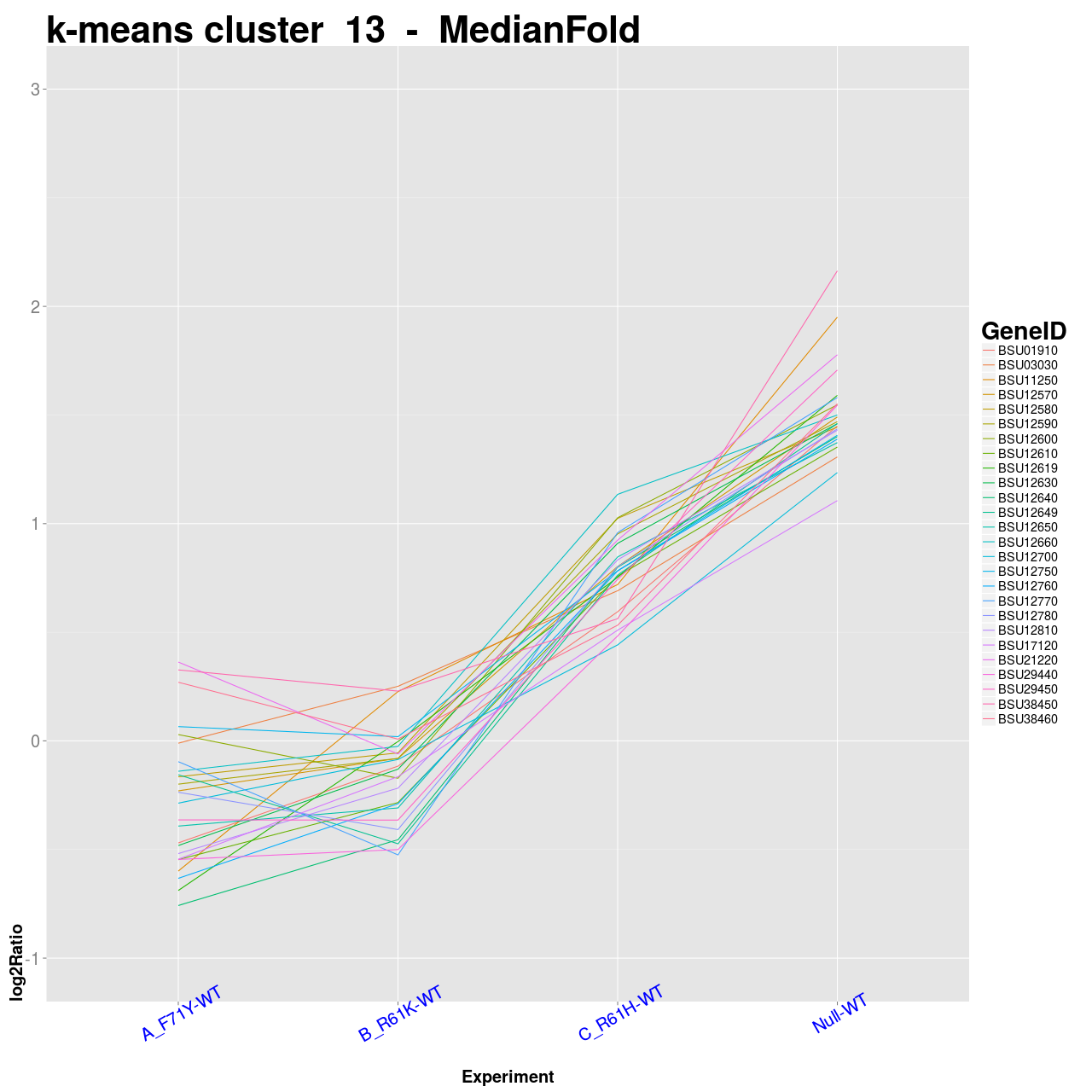

Supplement: Additional file 3: — Figure S3; k-means clustering of differentially expressed genes in the mutants. (ZIP 31925 kb) [file 12864_2015_1834_MOESM3_ESM.zip › Brinsmade.MedianFold.kmeans_plot_cluster.13.png]

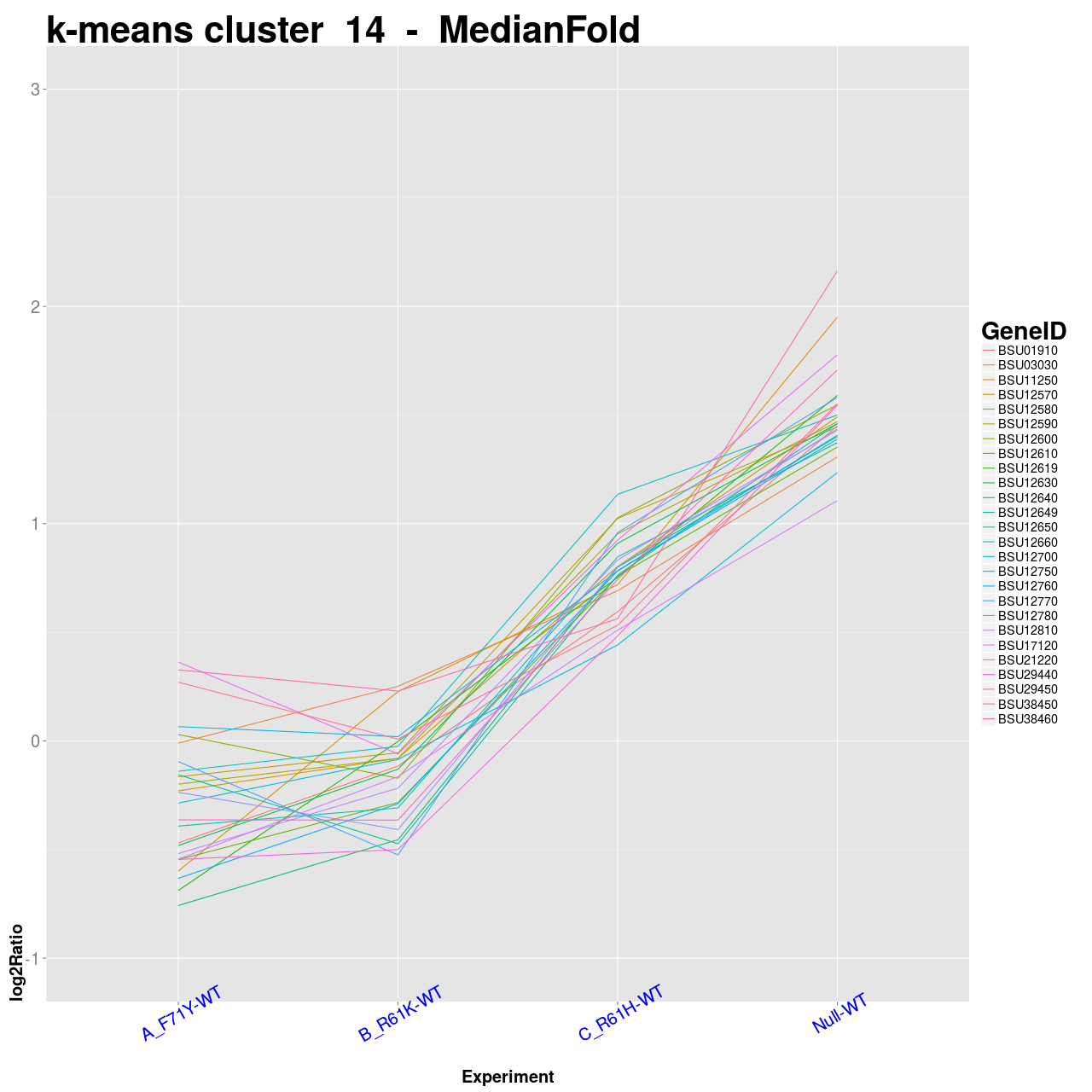

Supplement: Additional file 3: — Figure S3; k-means clustering of differentially expressed genes in the mutants. (ZIP 31925 kb) [file 12864_2015_1834_MOESM3_ESM.zip › Brinsmade.MedianFold.kmeans_plot_cluster.14.png]

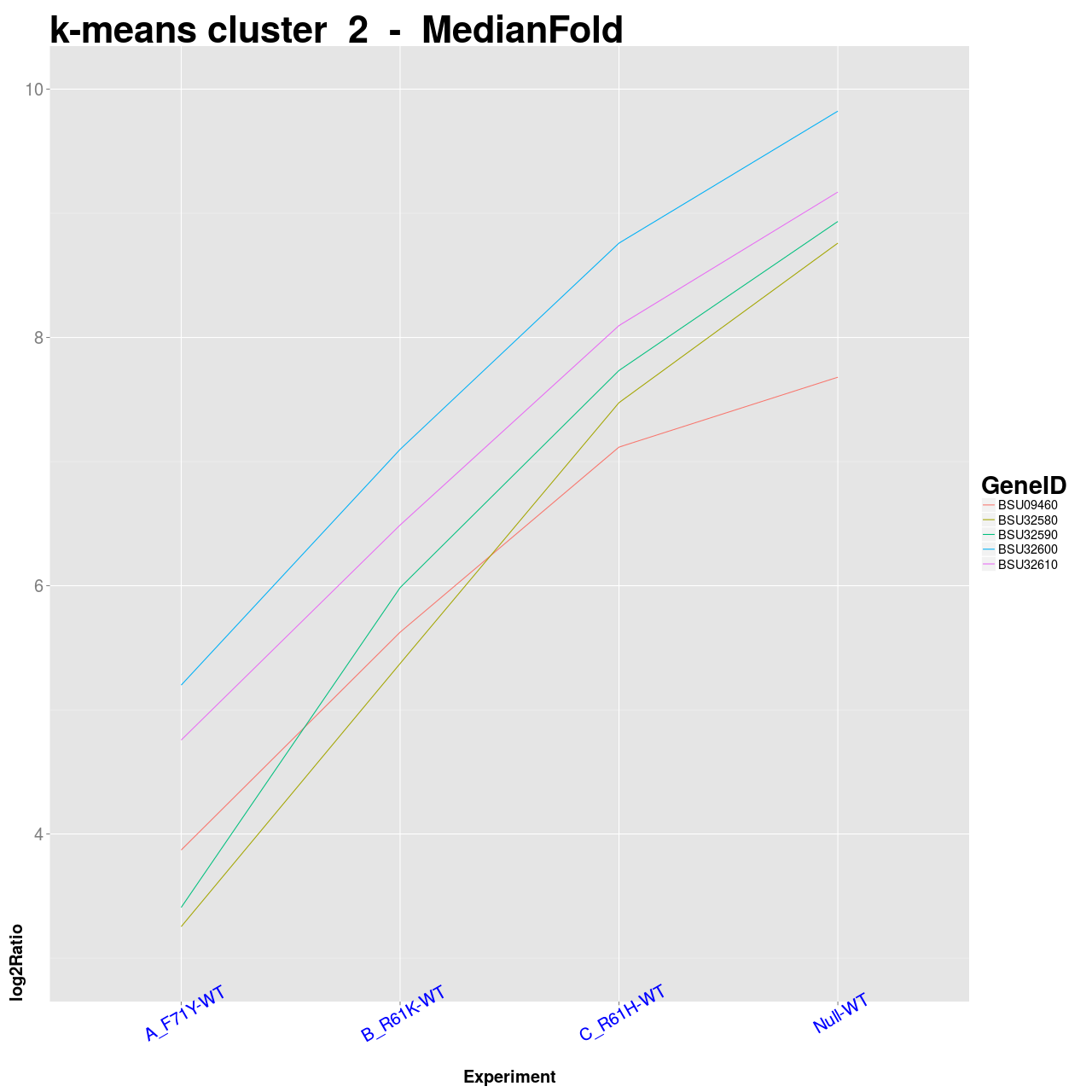

Supplement: Additional file 3: — Figure S3; k-means clustering of differentially expressed genes in the mutants. (ZIP 31925 kb) [file 12864_2015_1834_MOESM3_ESM.zip › Brinsmade.MedianFold.kmeans_plot_cluster.2.png]

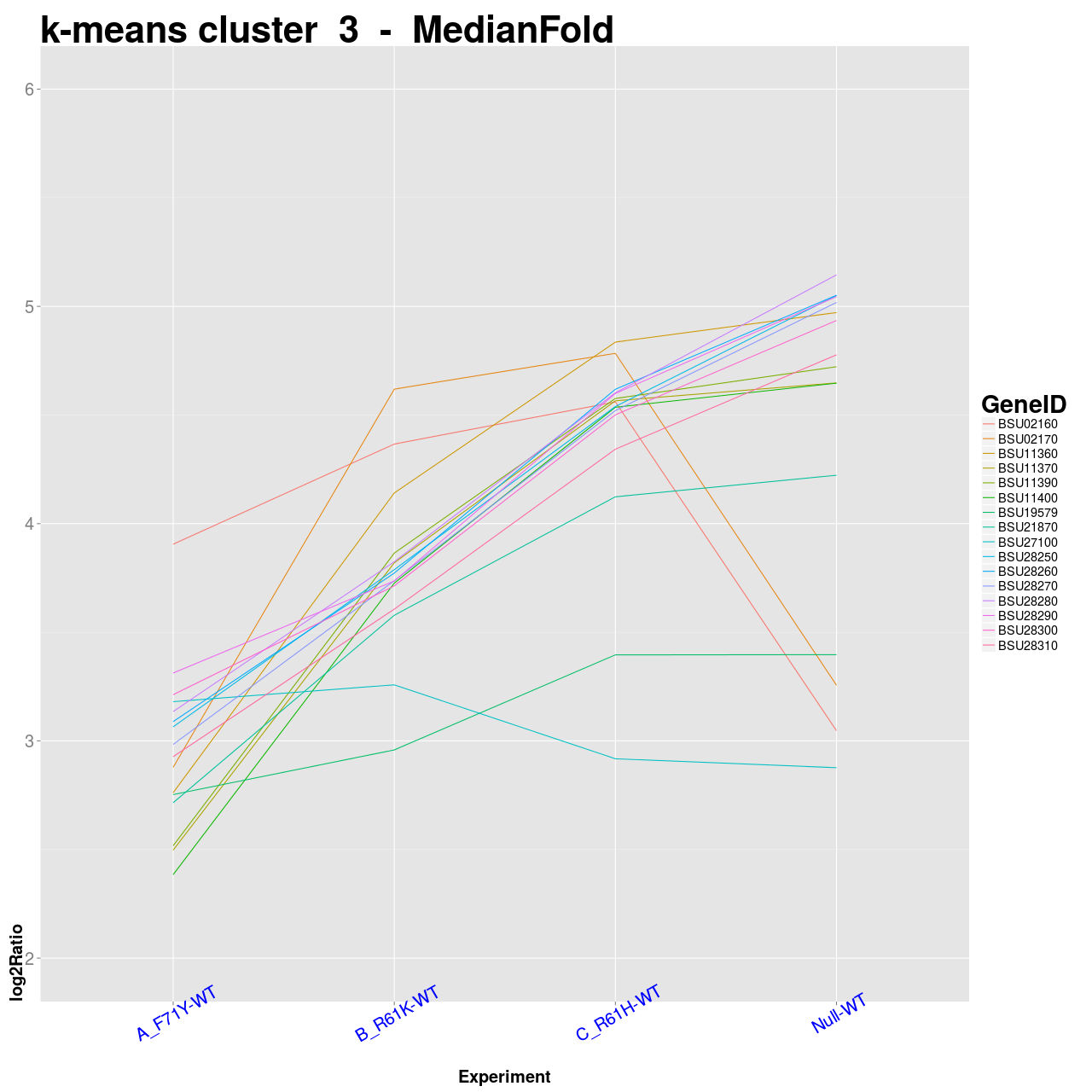

Supplement: Additional file 3: — Figure S3; k-means clustering of differentially expressed genes in the mutants. (ZIP 31925 kb) [file 12864_2015_1834_MOESM3_ESM.zip › Brinsmade.MedianFold.kmeans_plot_cluster.3.png]

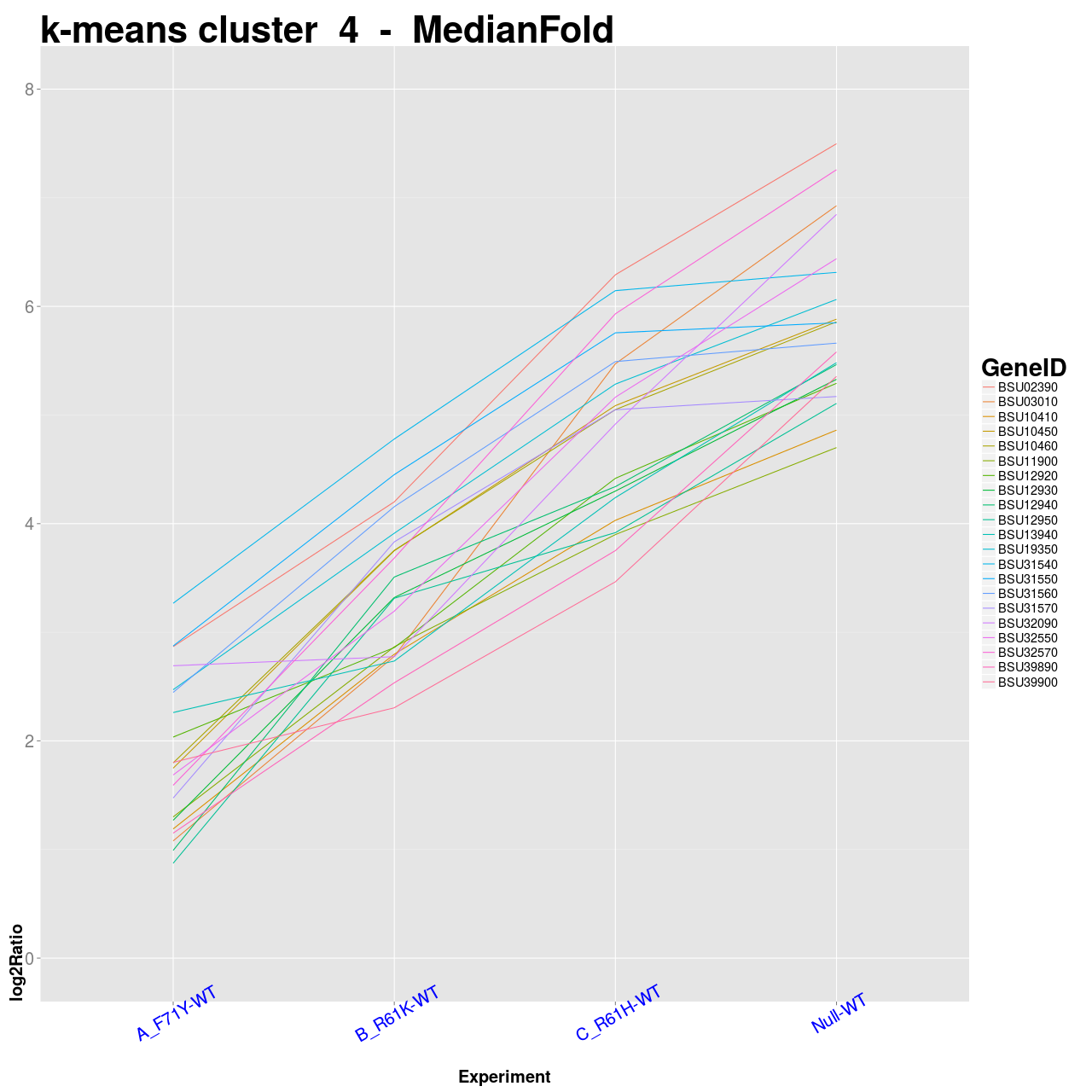

Supplement: Additional file 3: — Figure S3; k-means clustering of differentially expressed genes in the mutants. (ZIP 31925 kb) [file 12864_2015_1834_MOESM3_ESM.zip › Brinsmade.MedianFold.kmeans_plot_cluster.4.png]

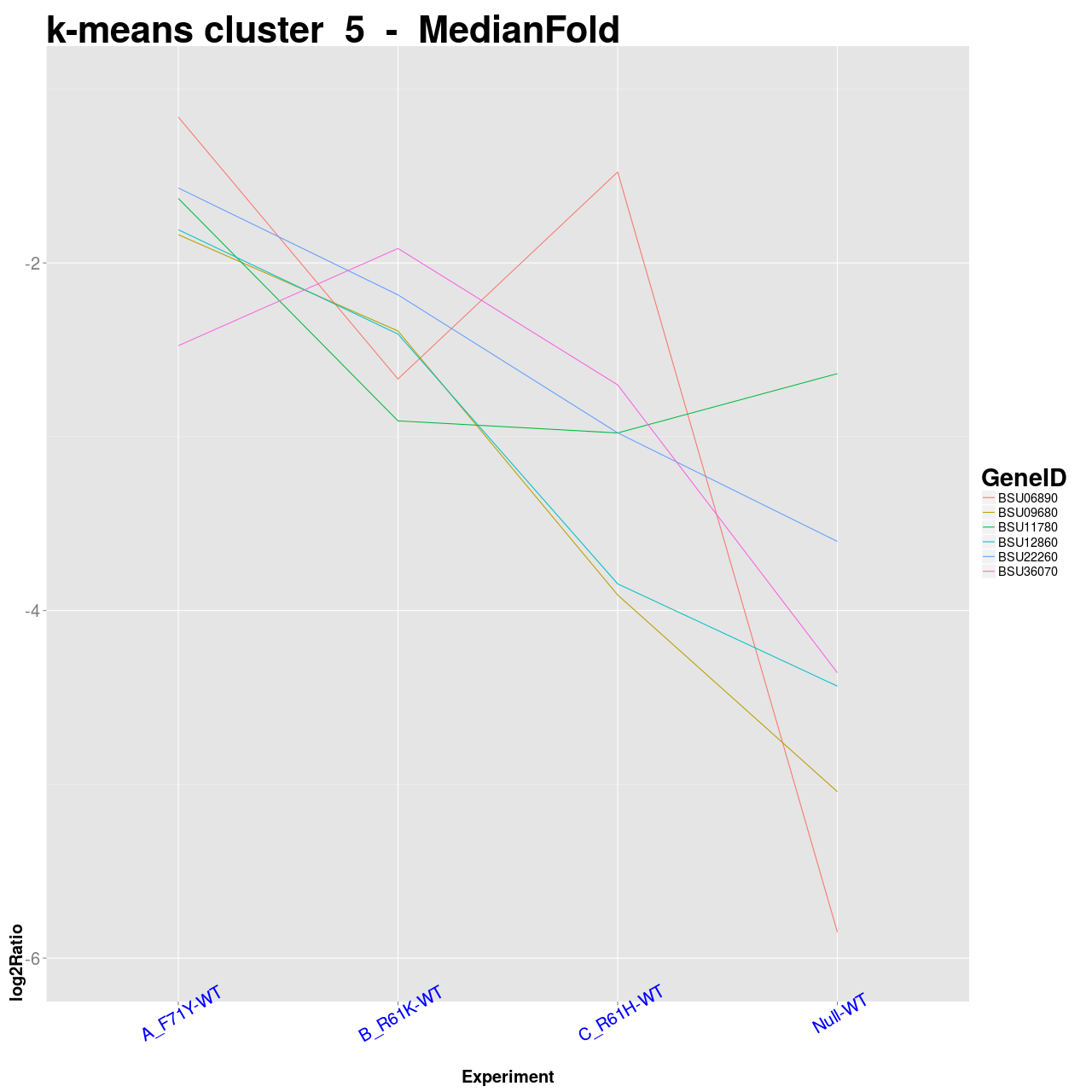

Supplement: Additional file 3: — Figure S3; k-means clustering of differentially expressed genes in the mutants. (ZIP 31925 kb) [file 12864_2015_1834_MOESM3_ESM.zip › Brinsmade.MedianFold.kmeans_plot_cluster.5.png]

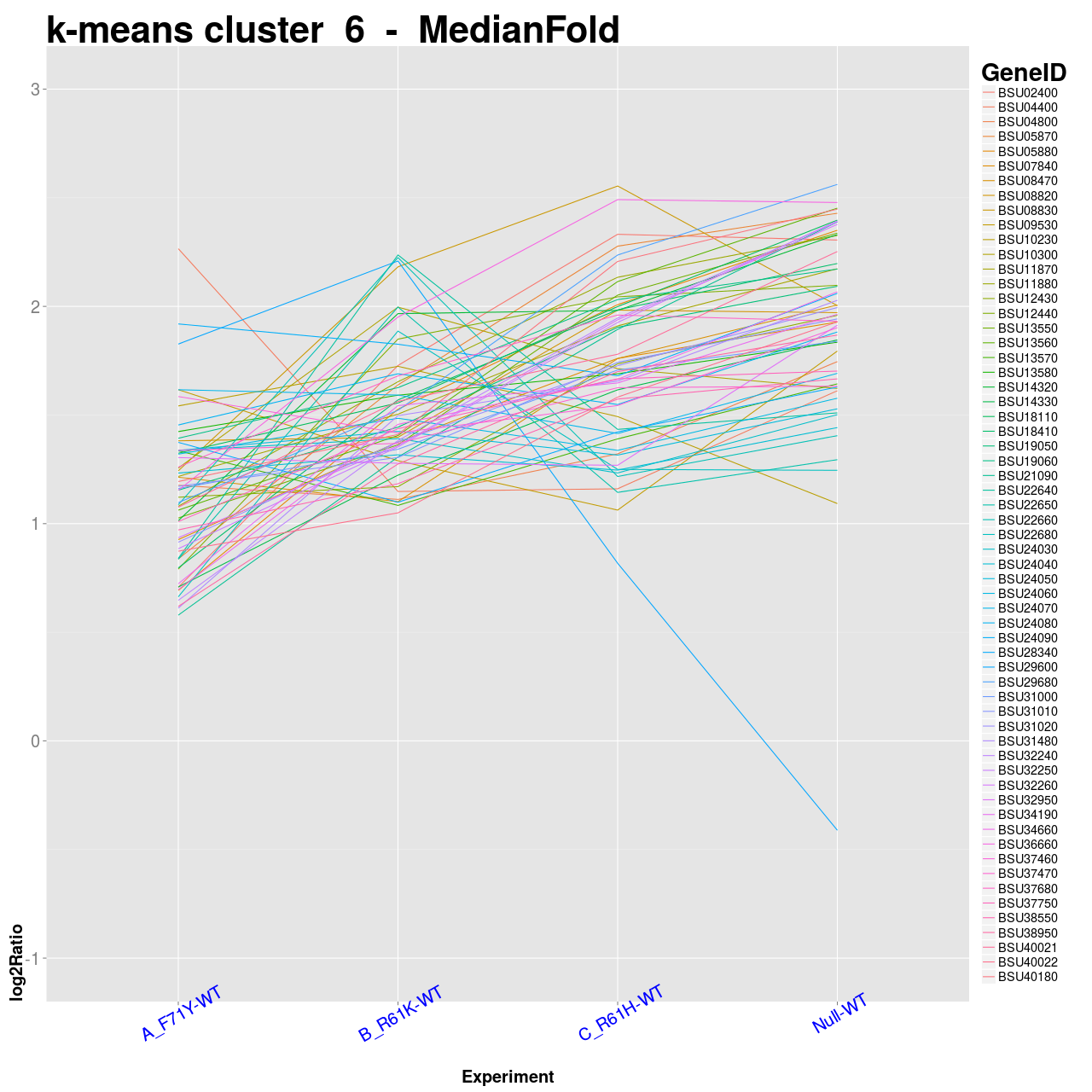

Supplement: Additional file 3: — Figure S3; k-means clustering of differentially expressed genes in the mutants. (ZIP 31925 kb) [file 12864_2015_1834_MOESM3_ESM.zip › Brinsmade.MedianFold.kmeans_plot_cluster.6.png]

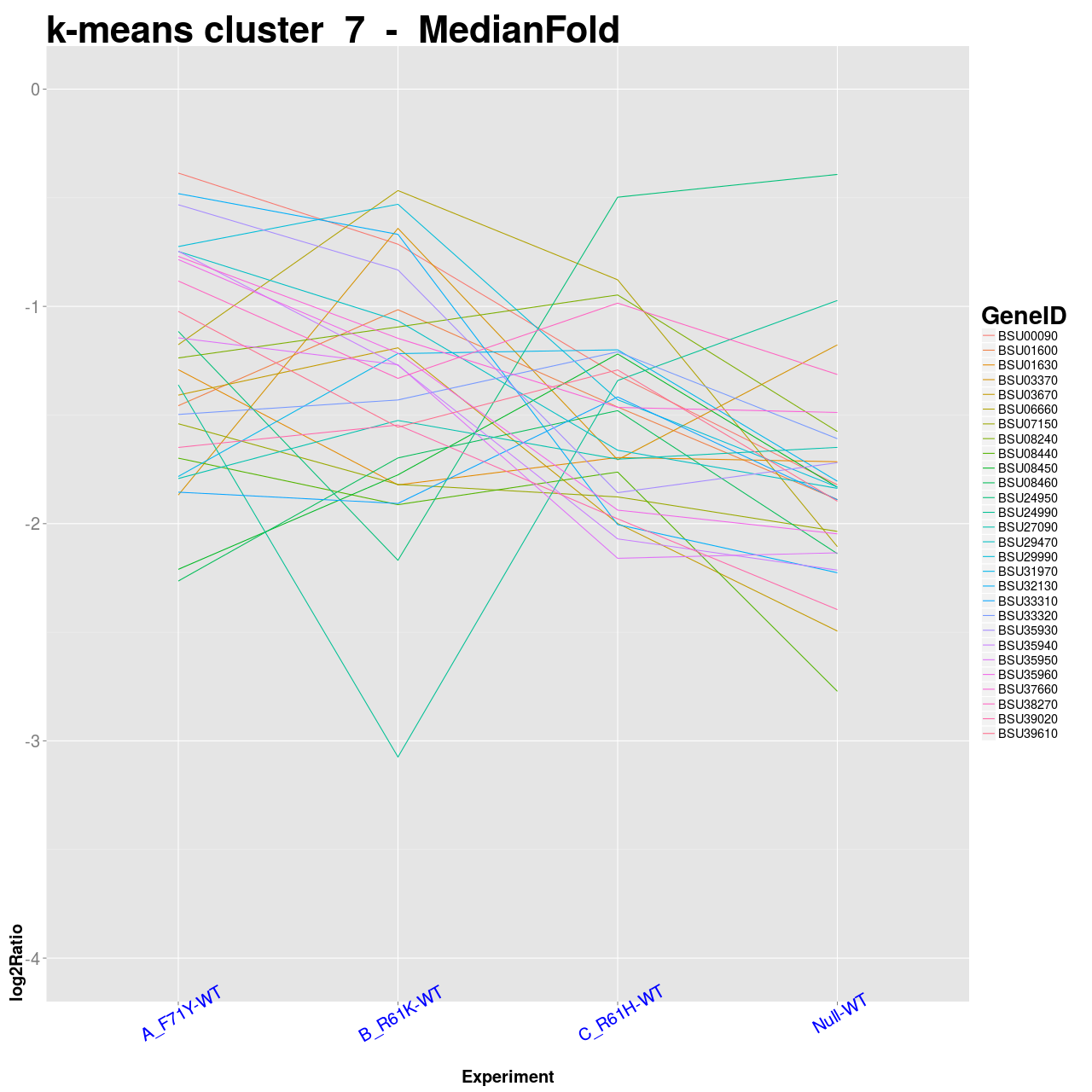

Supplement: Additional file 3: — Figure S3; k-means clustering of differentially expressed genes in the mutants. (ZIP 31925 kb) [file 12864_2015_1834_MOESM3_ESM.zip › Brinsmade.MedianFold.kmeans_plot_cluster.7.png]

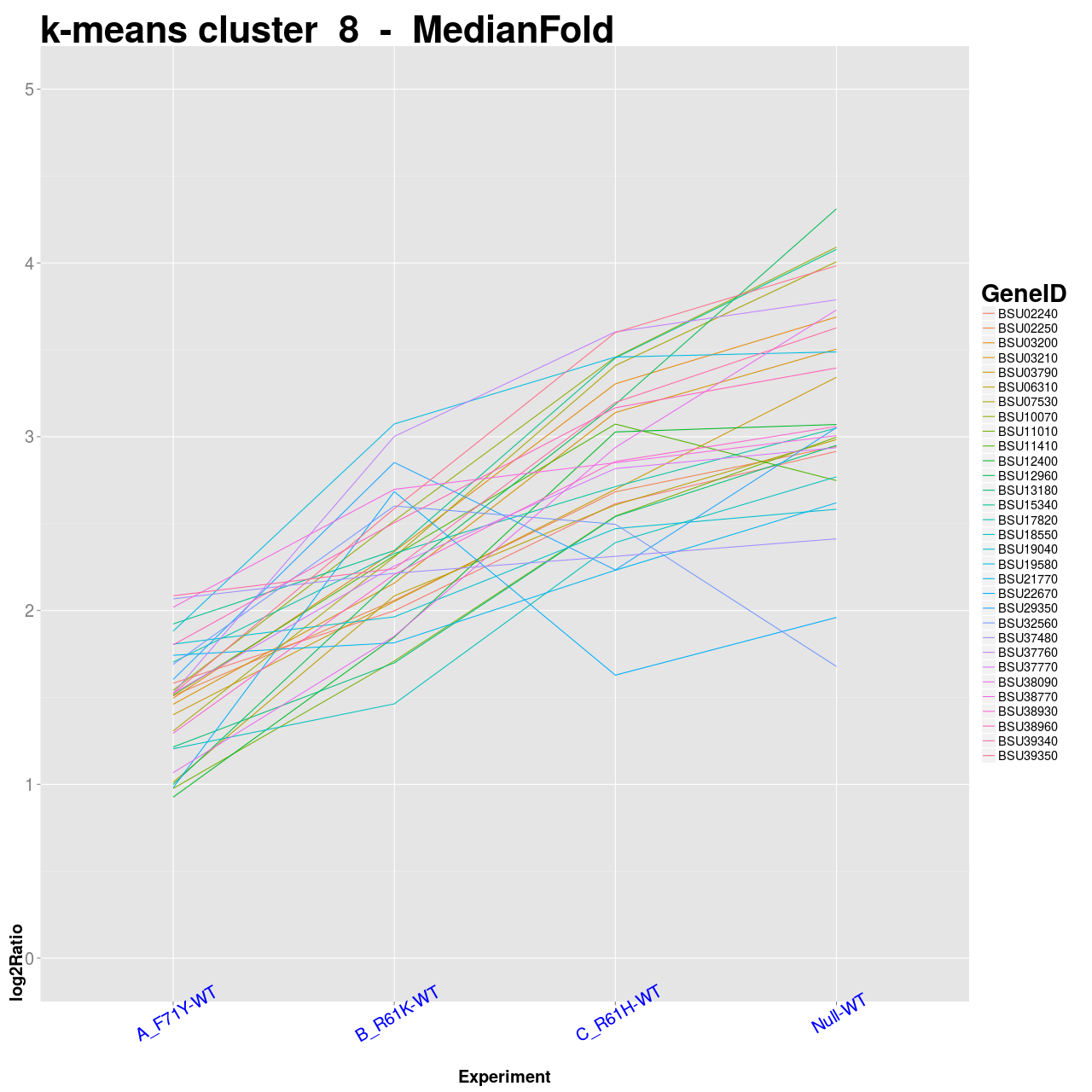

Supplement: Additional file 3: — Figure S3; k-means clustering of differentially expressed genes in the mutants. (ZIP 31925 kb) [file 12864_2015_1834_MOESM3_ESM.zip › Brinsmade.MedianFold.kmeans_plot_cluster.8.png]

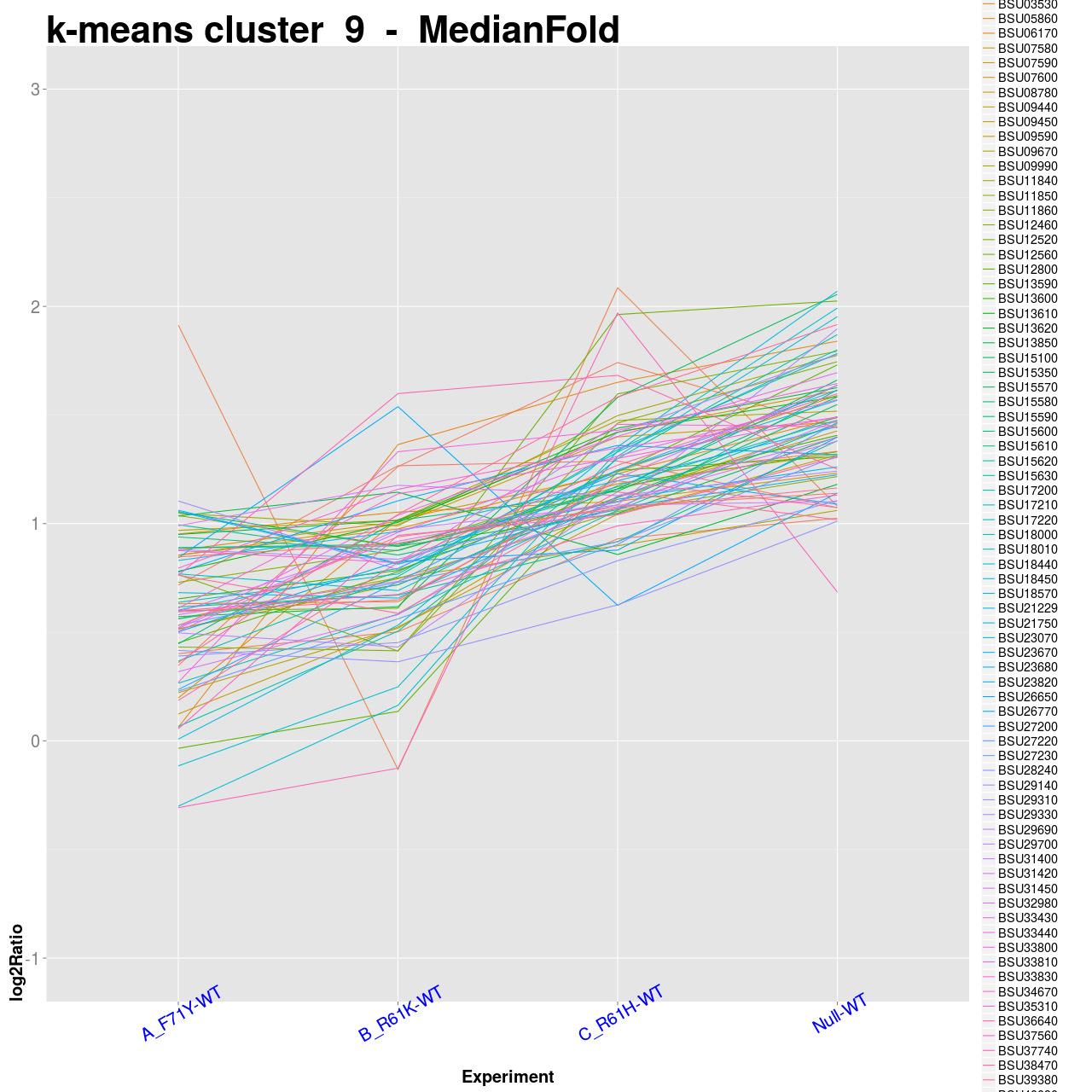

Supplement: Additional file 3: — Figure S3; k-means clustering of differentially expressed genes in the mutants. (ZIP 31925 kb) [file 12864_2015_1834_MOESM3_ESM.zip › Brinsmade.MedianFold.kmeans_plot_cluster.9.png]

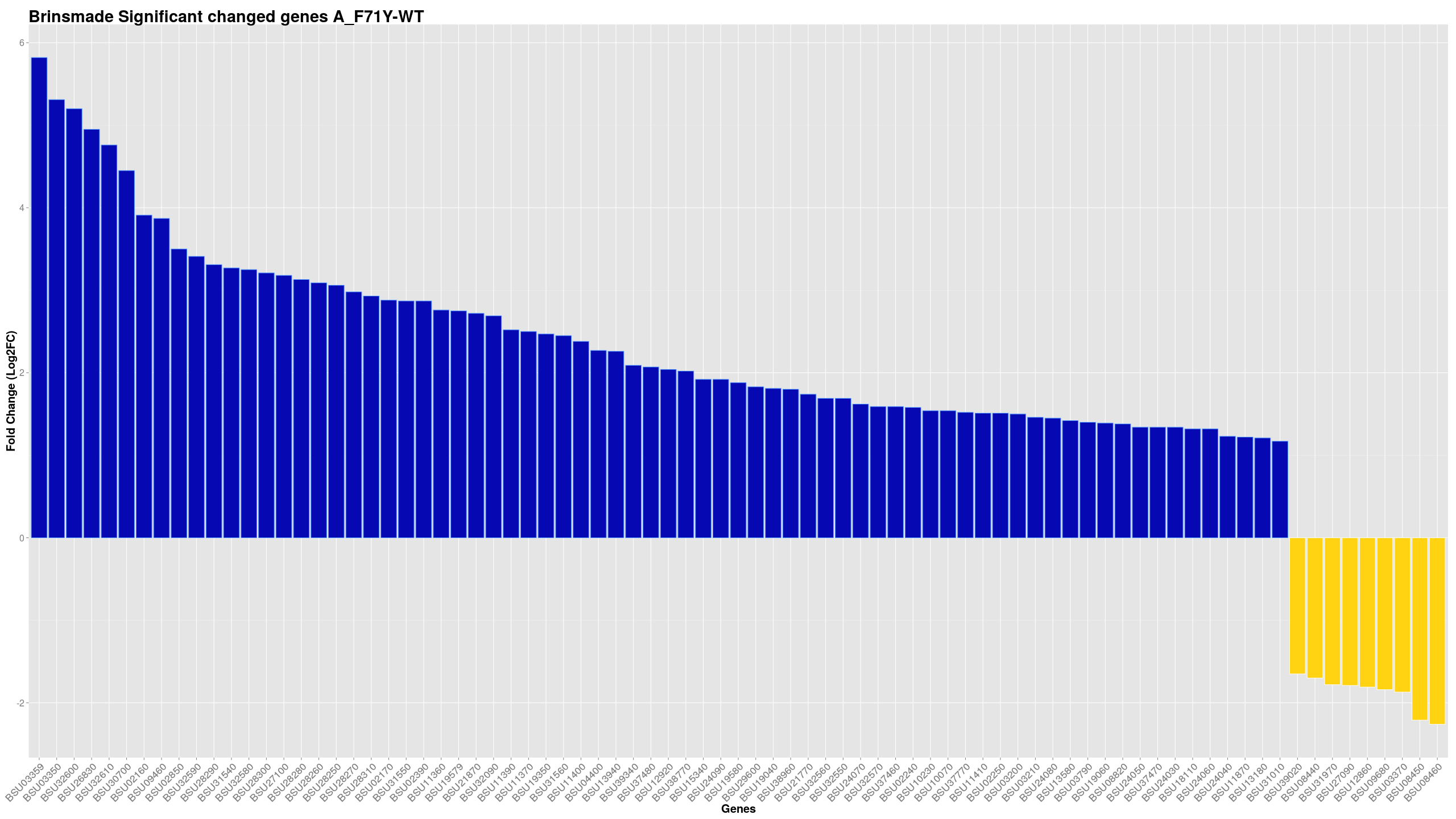

Supplement: Additional file 3: — Figure S3; k-means clustering of differentially expressed genes in the mutants. (ZIP 31925 kb) [file 12864_2015_1834_MOESM3_ESM.zip › Brinsmade.Significant_Changed_Genes.1.A_F71Y-WT.png]

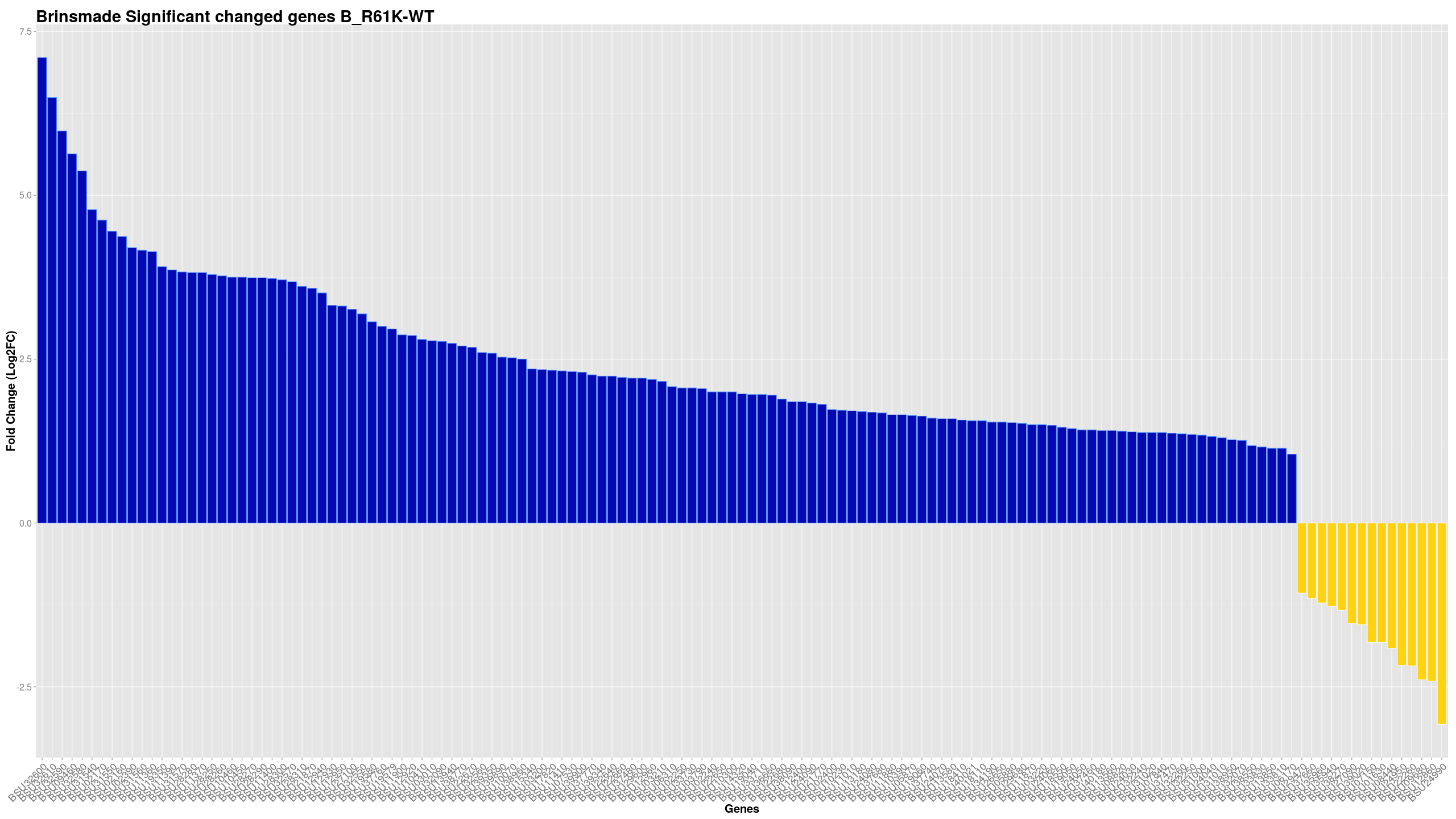

Supplement: Additional file 3: — Figure S3; k-means clustering of differentially expressed genes in the mutants. (ZIP 31925 kb) [file 12864_2015_1834_MOESM3_ESM.zip › Brinsmade.Significant_Changed_Genes.2.B_R61K-WT.png]

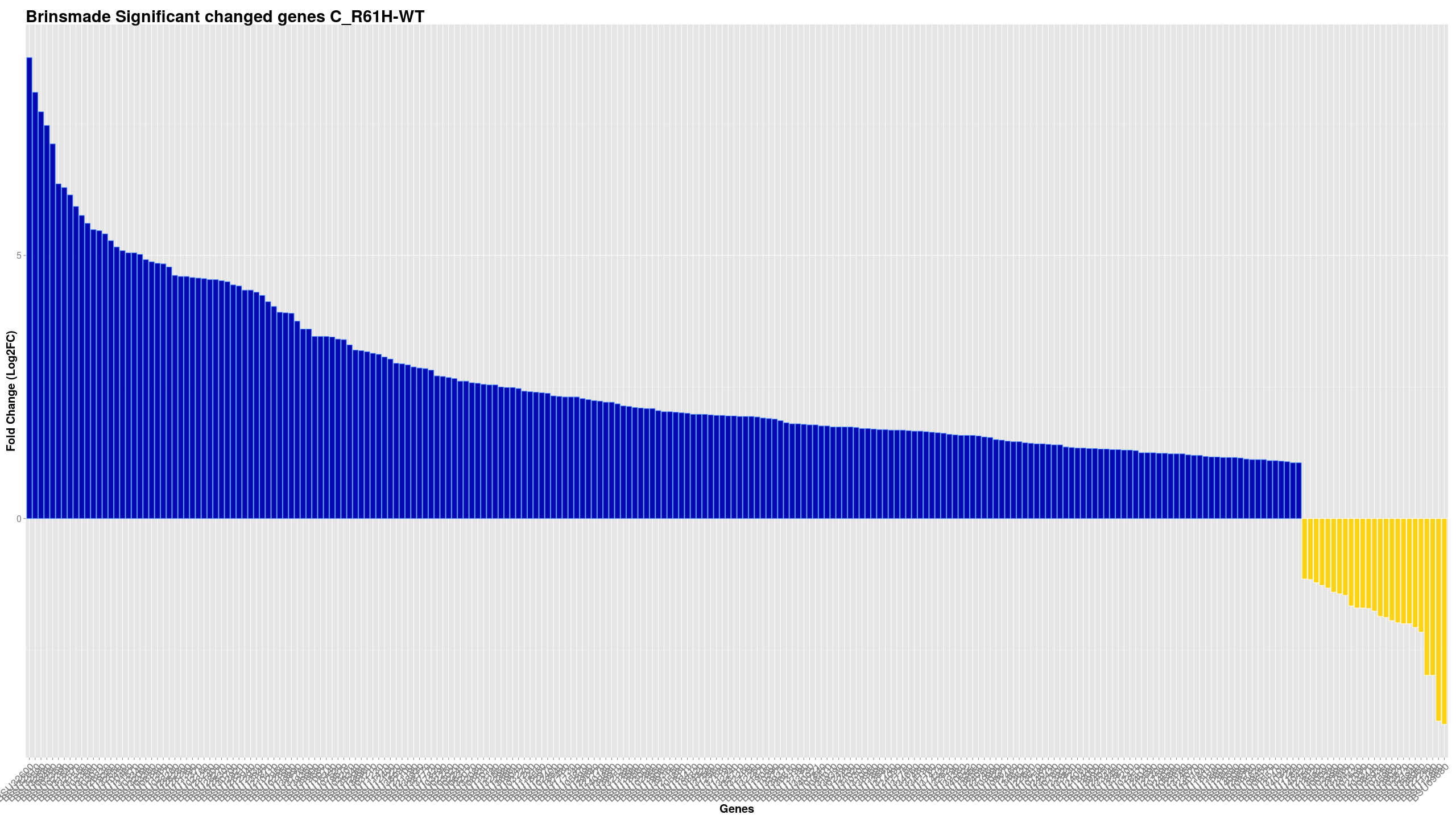

Supplement: Additional file 3: — Figure S3; k-means clustering of differentially expressed genes in the mutants. (ZIP 31925 kb) [file 12864_2015_1834_MOESM3_ESM.zip › Brinsmade.Significant_Changed_Genes.3.C_R61H-WT.png]

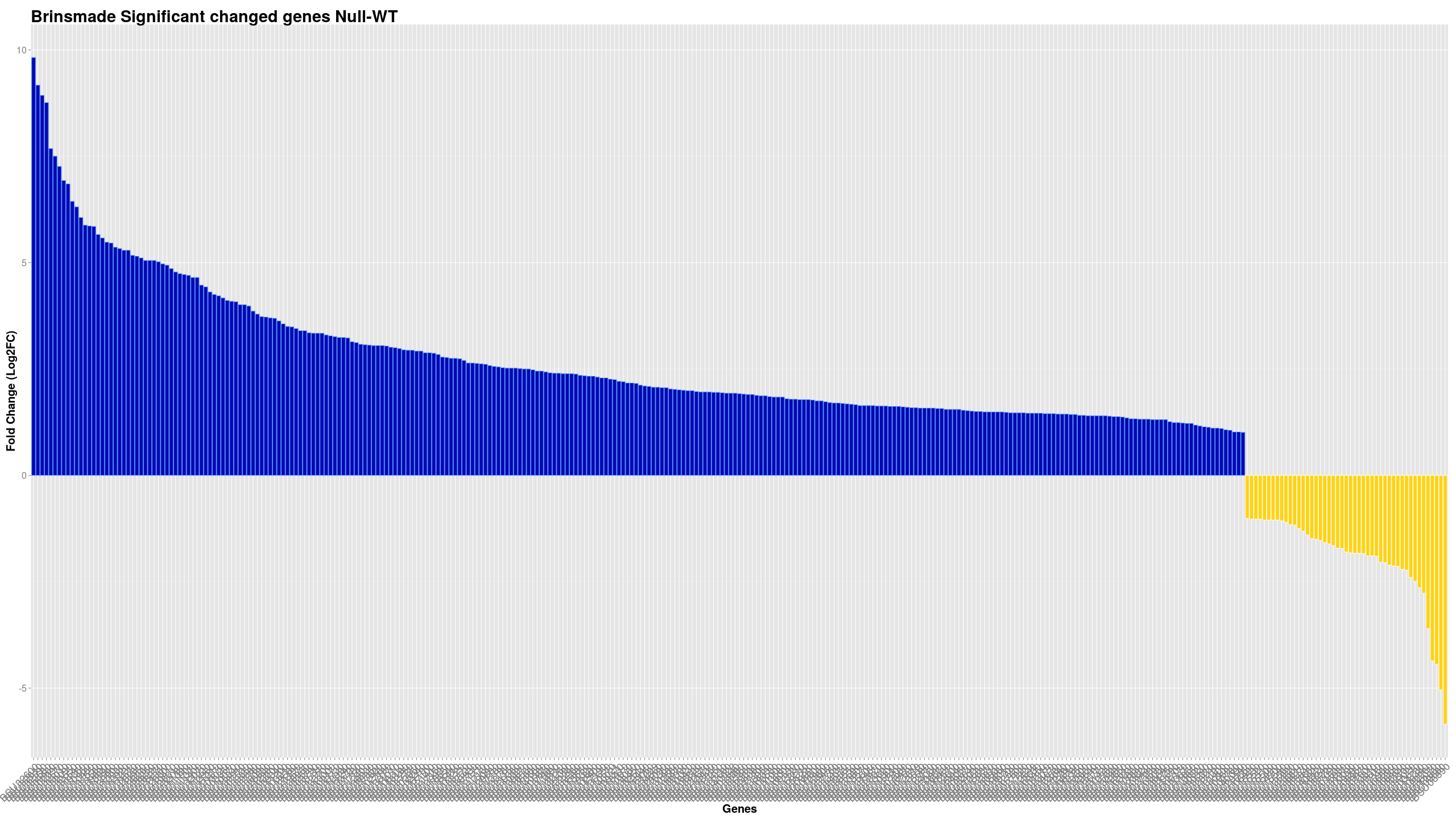

Supplement: Additional file 3: — Figure S3; k-means clustering of differentially expressed genes in the mutants. (ZIP 31925 kb) [file 12864_2015_1834_MOESM3_ESM.zip › Brinsmade.Significant_Changed_Genes.4.Null-WT.png]

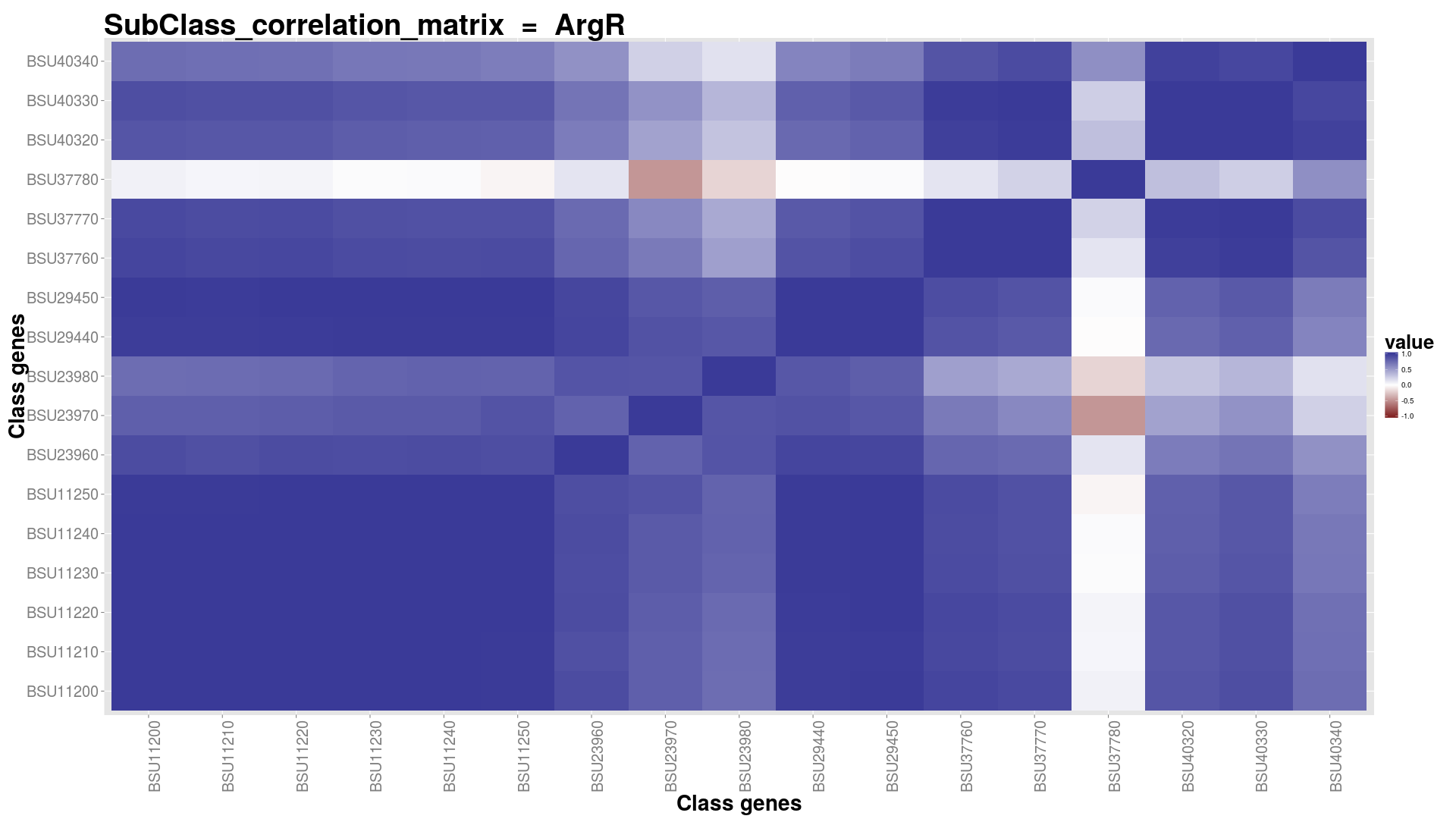

Supplement: Additional file 3: — Figure S3; k-means clustering of differentially expressed genes in the mutants. (ZIP 31925 kb) [file 12864_2015_1834_MOESM3_ESM.zip › Brinsmade.SubClass_correlation_matrix.ArgR.png]

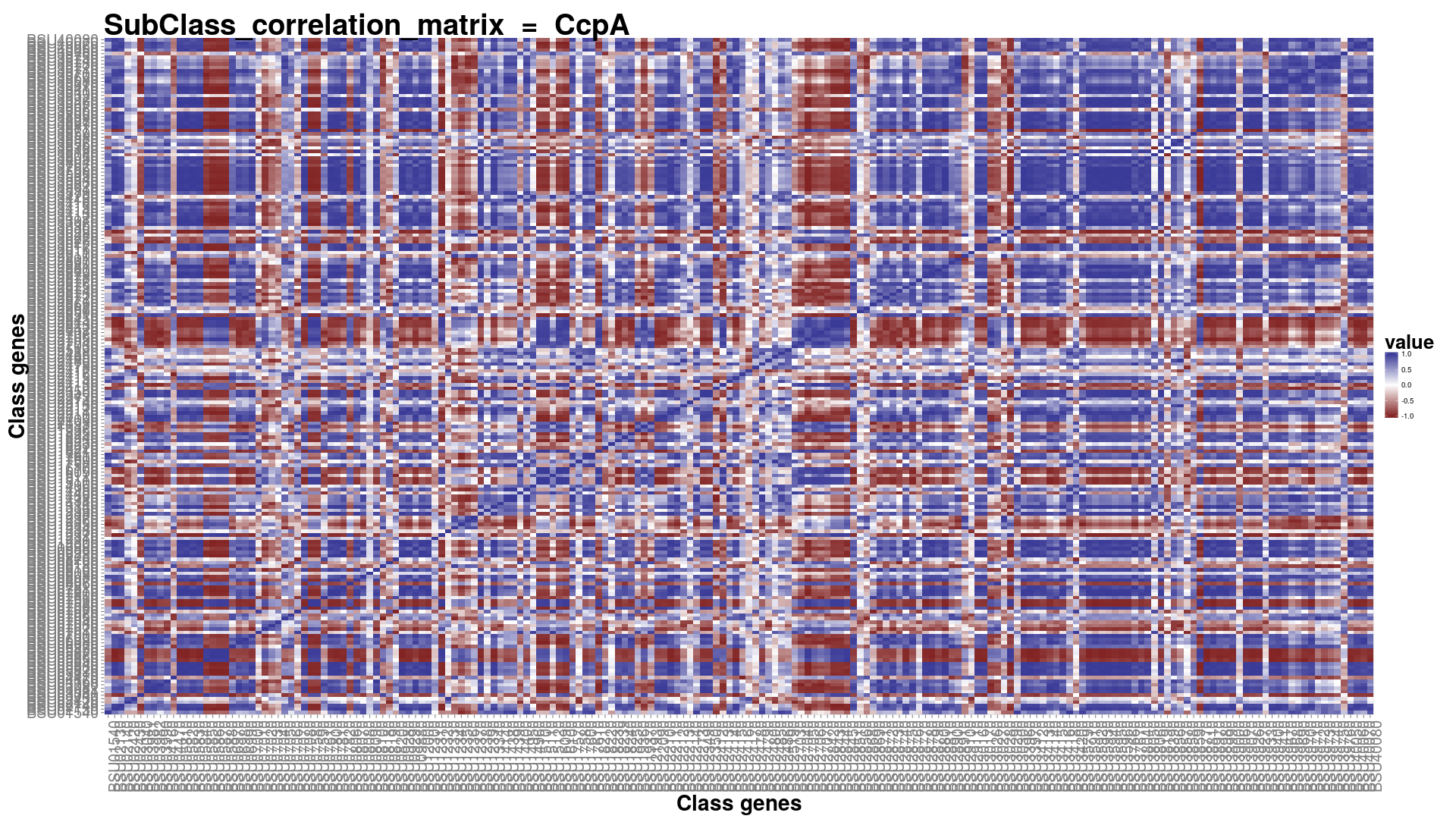

Supplement: Additional file 3: — Figure S3; k-means clustering of differentially expressed genes in the mutants. (ZIP 31925 kb) [file 12864_2015_1834_MOESM3_ESM.zip › Brinsmade.SubClass_correlation_matrix.CcpA.png]

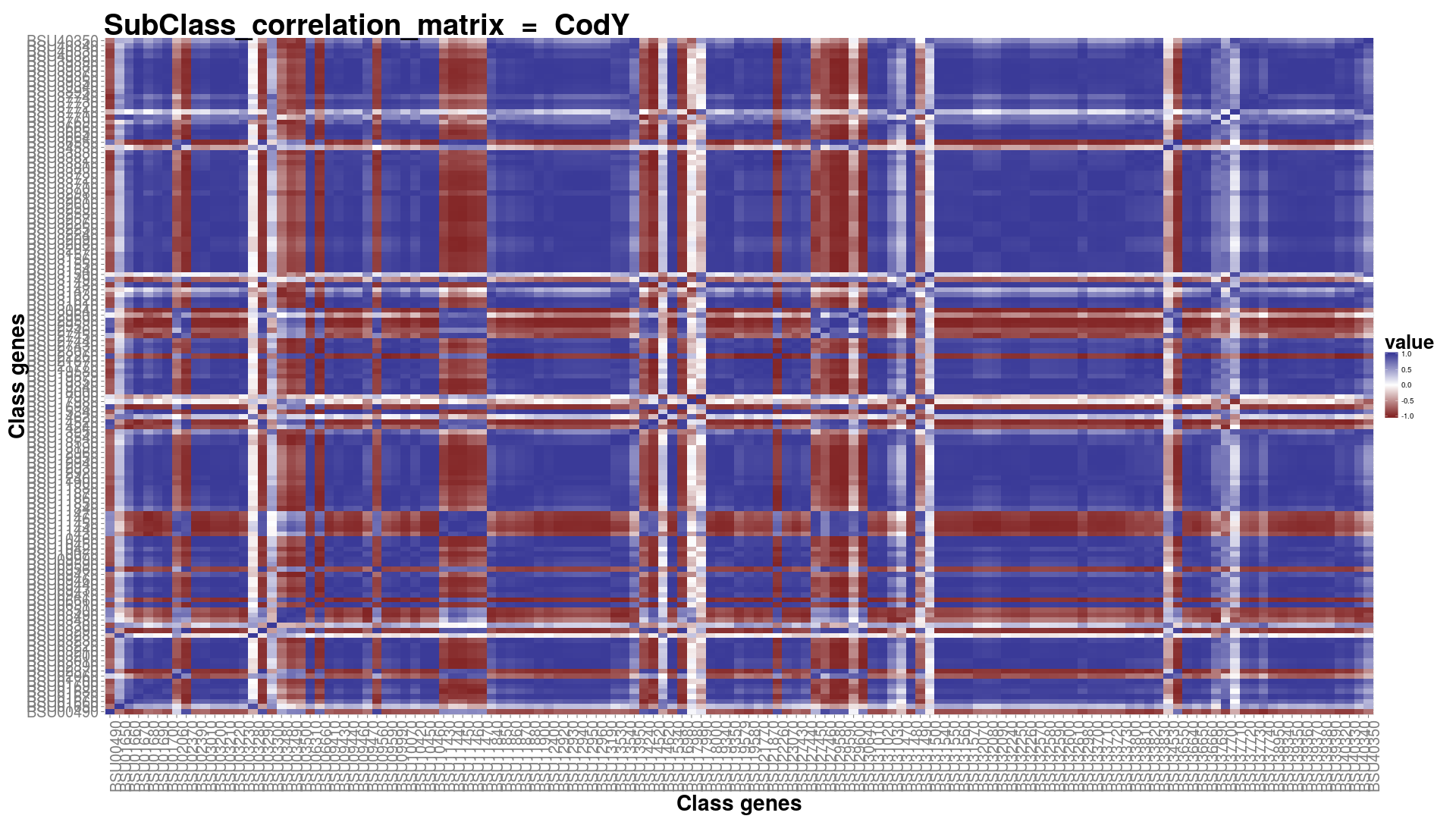

Supplement: Additional file 3: — Figure S3; k-means clustering of differentially expressed genes in the mutants. (ZIP 31925 kb) [file 12864_2015_1834_MOESM3_ESM.zip › Brinsmade.SubClass_correlation_matrix.CodY.png]

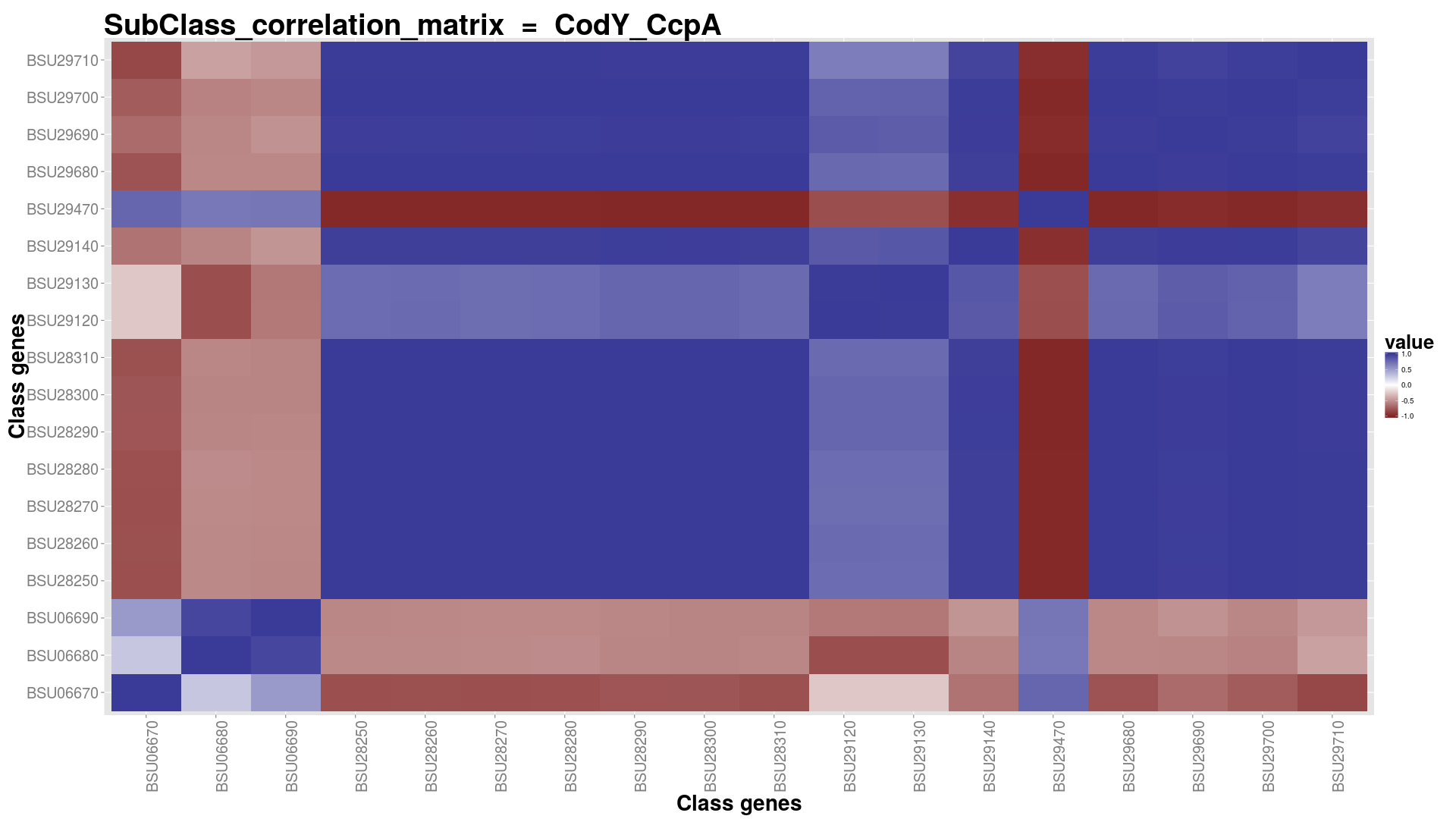

Supplement: Additional file 3: — Figure S3; k-means clustering of differentially expressed genes in the mutants. (ZIP 31925 kb) [file 12864_2015_1834_MOESM3_ESM.zip › Brinsmade.SubClass_correlation_matrix.CodY_CcpA.png]

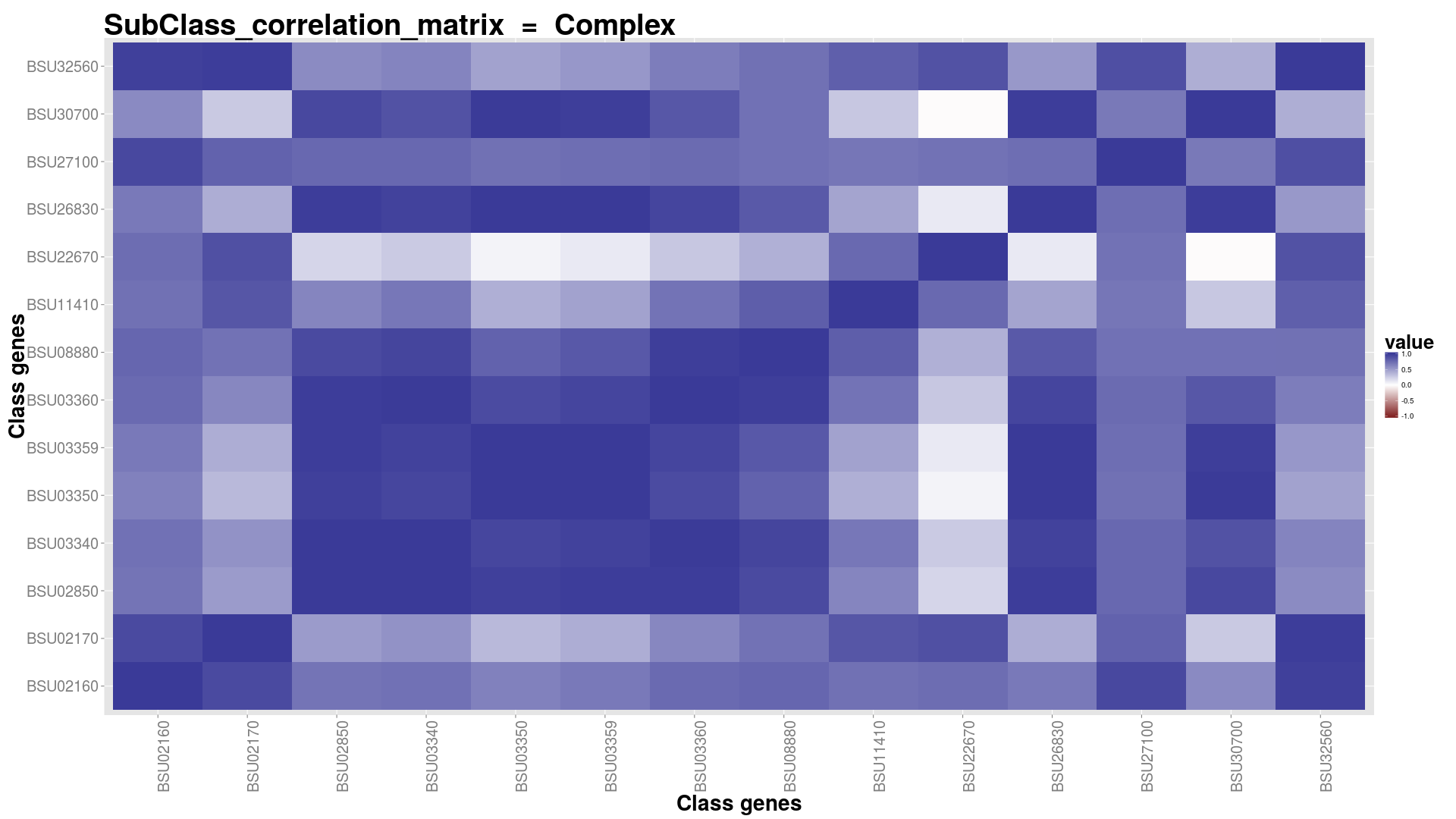

Supplement: Additional file 3: — Figure S3; k-means clustering of differentially expressed genes in the mutants. (ZIP 31925 kb) [file 12864_2015_1834_MOESM3_ESM.zip › Brinsmade.SubClass_correlation_matrix.Complex.png]

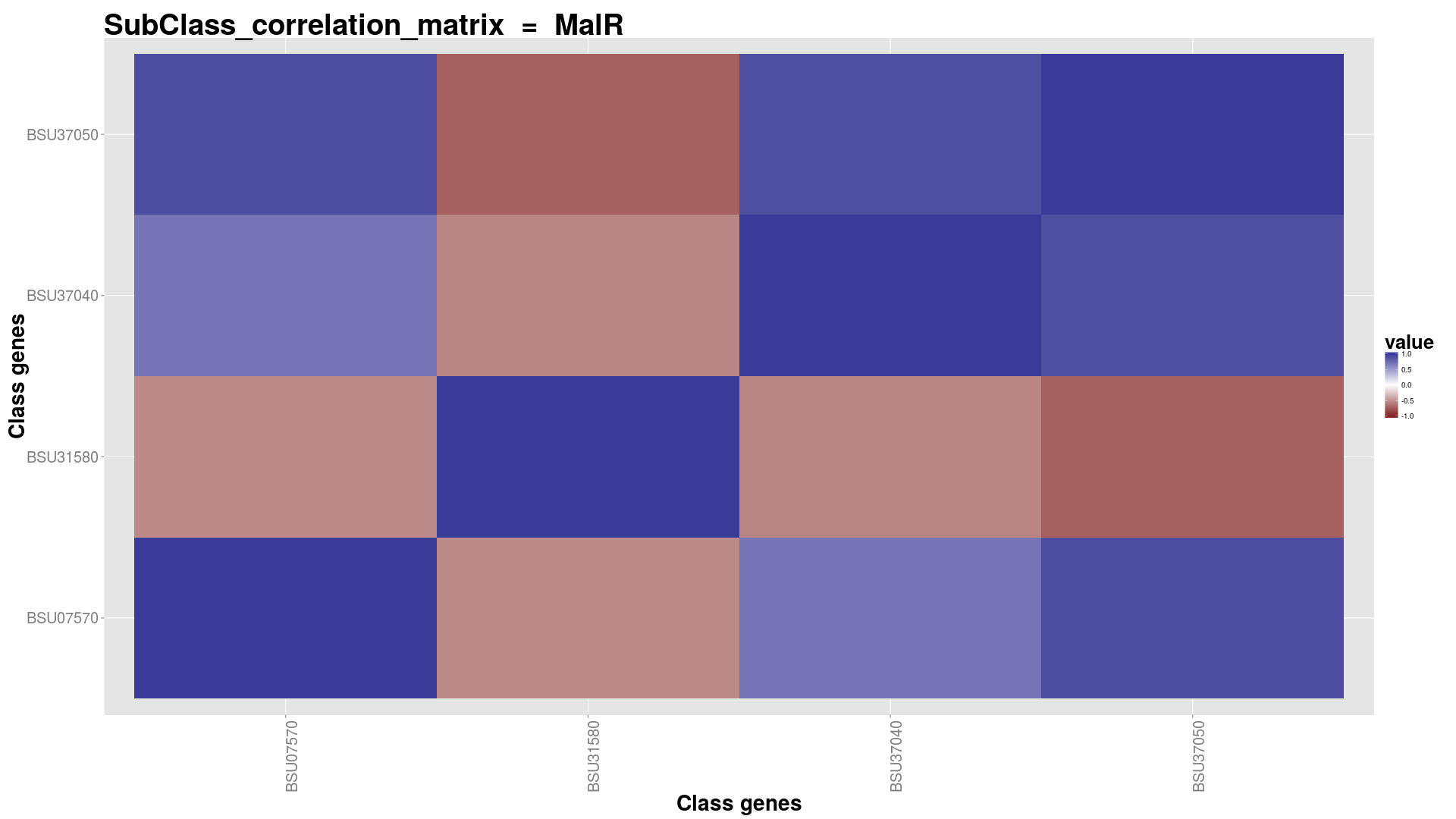

Supplement: Additional file 3: — Figure S3; k-means clustering of differentially expressed genes in the mutants. (ZIP 31925 kb) [file 12864_2015_1834_MOESM3_ESM.zip › Brinsmade.SubClass_correlation_matrix.MalR.png]

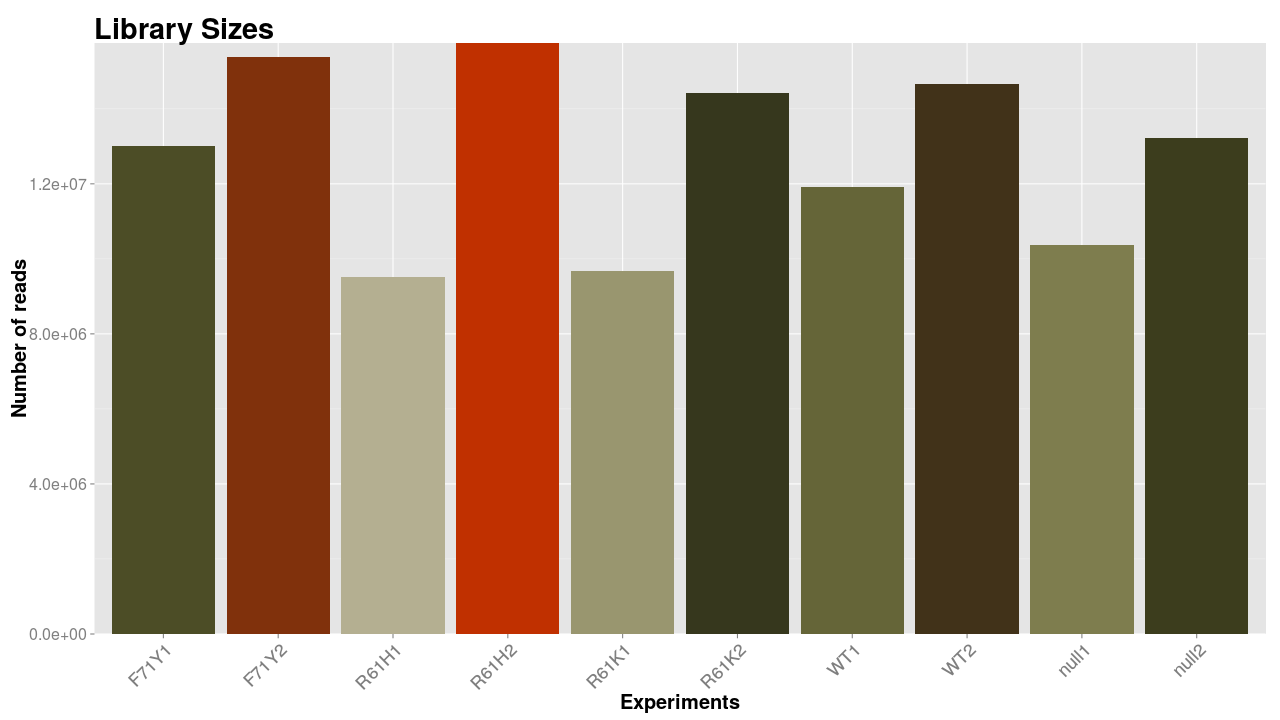

Supplement: Additional file 4: — Figures S4A and S4B; signal heatmaps of Class ‘Complex’ and Class ‘CodY’, respectively. (ZIP 1103 kb) [file 12864_2015_1834_MOESM4_ESM.zip › Figure_S2A.png]

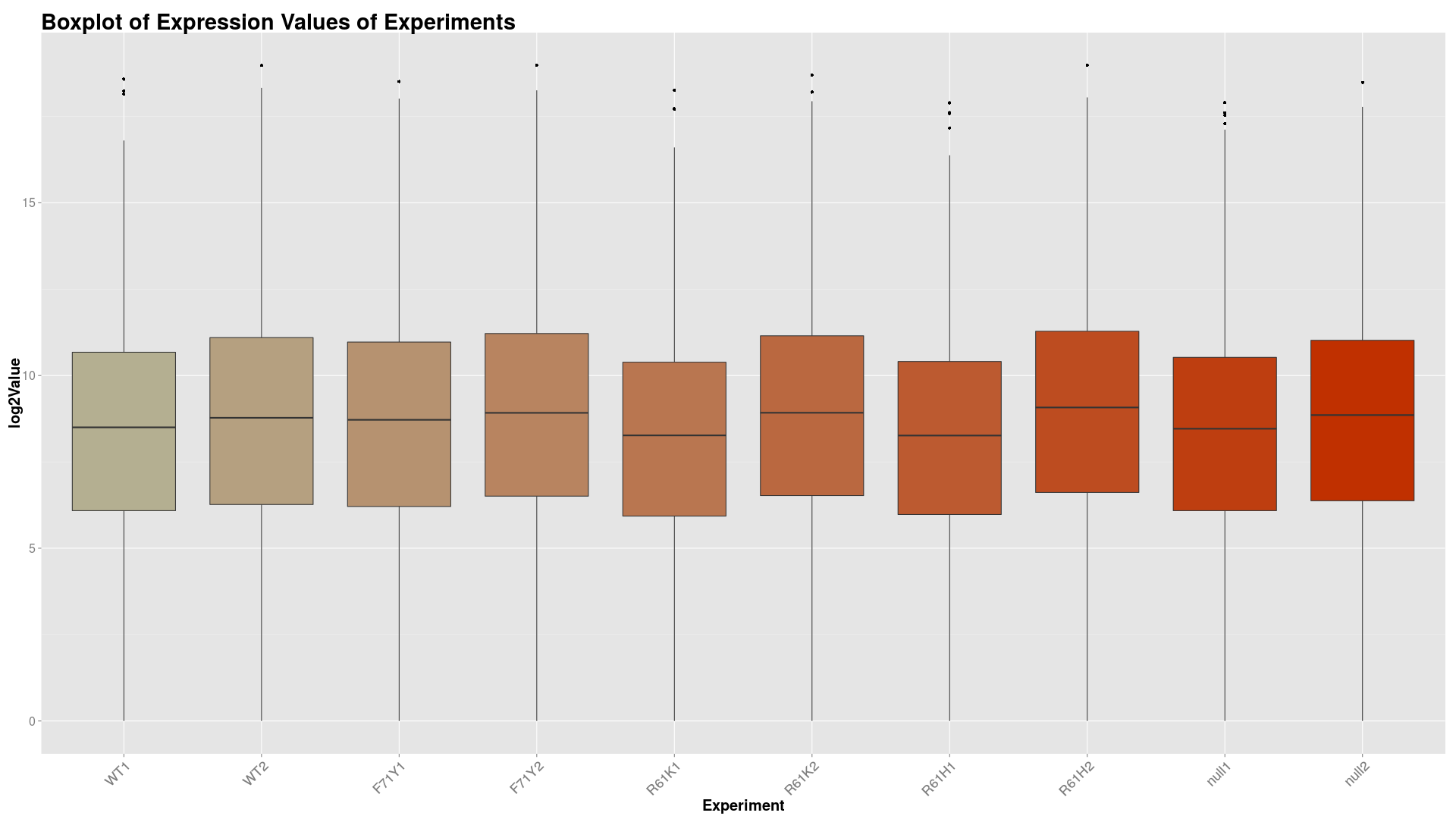

Supplement: Additional file 4: — Figures S4A and S4B; signal heatmaps of Class ‘Complex’ and Class ‘CodY’, respectively. (ZIP 1103 kb) [file 12864_2015_1834_MOESM4_ESM.zip › Figure_S2B.png]

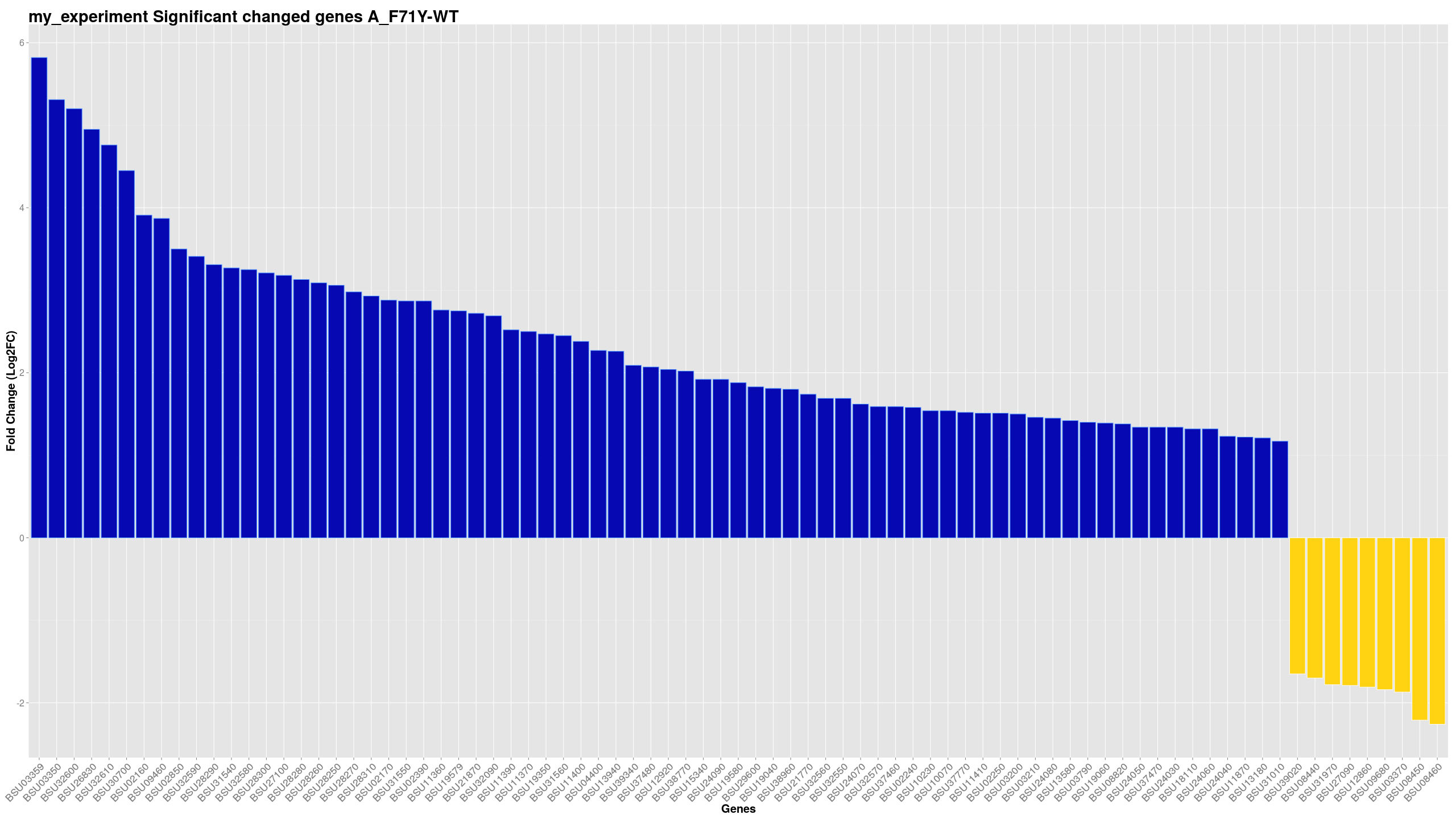

Supplement: Additional file 4: — Figures S4A and S4B; signal heatmaps of Class ‘Complex’ and Class ‘CodY’, respectively. (ZIP 1103 kb) [file 12864_2015_1834_MOESM4_ESM.zip › Figure_S2D.png]

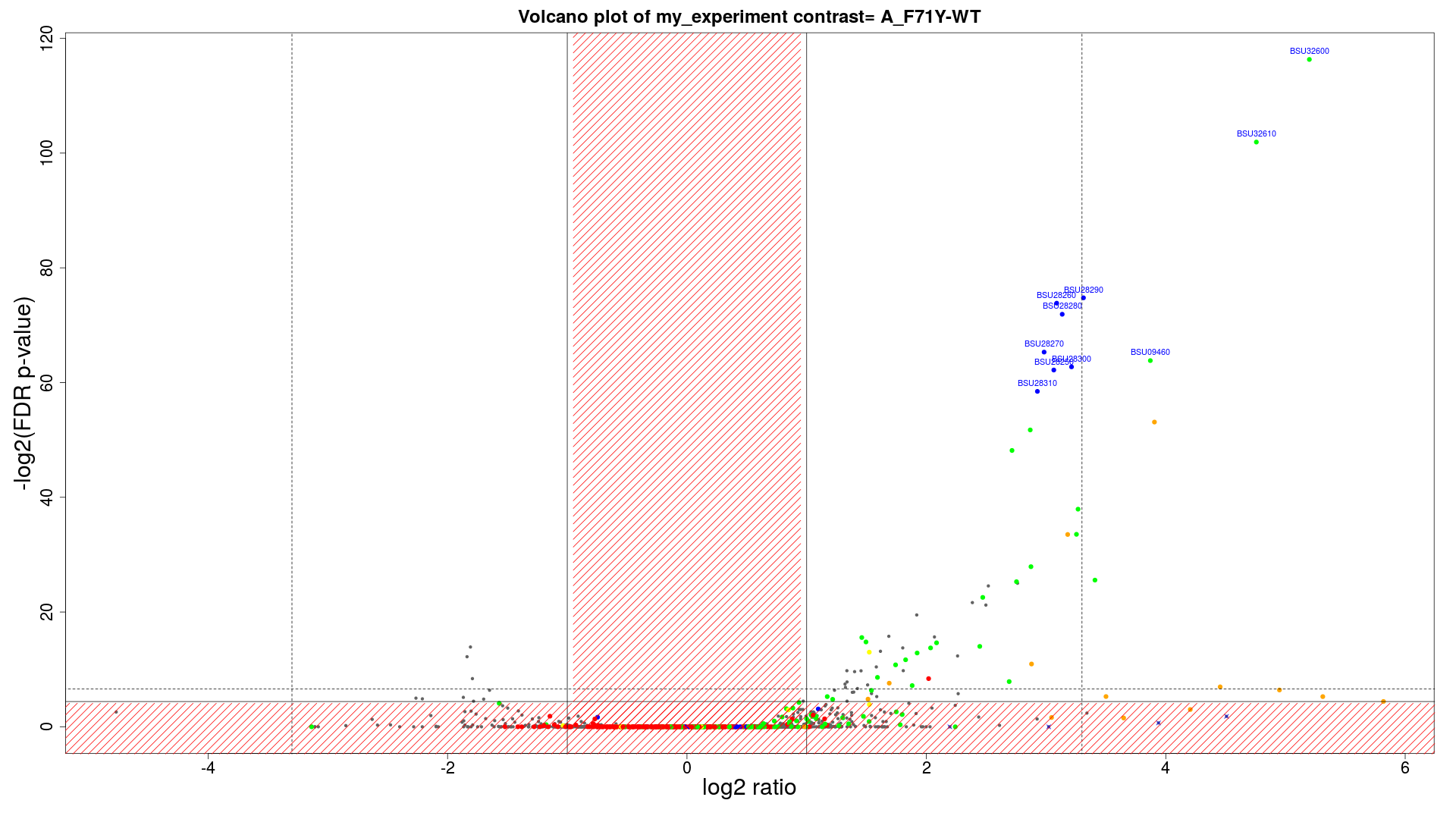

Supplement: Additional file 4: — Figures S4A and S4B; signal heatmaps of Class ‘Complex’ and Class ‘CodY’, respectively. (ZIP 1103 kb) [file 12864_2015_1834_MOESM4_ESM.zip › Figure_S2F.png]

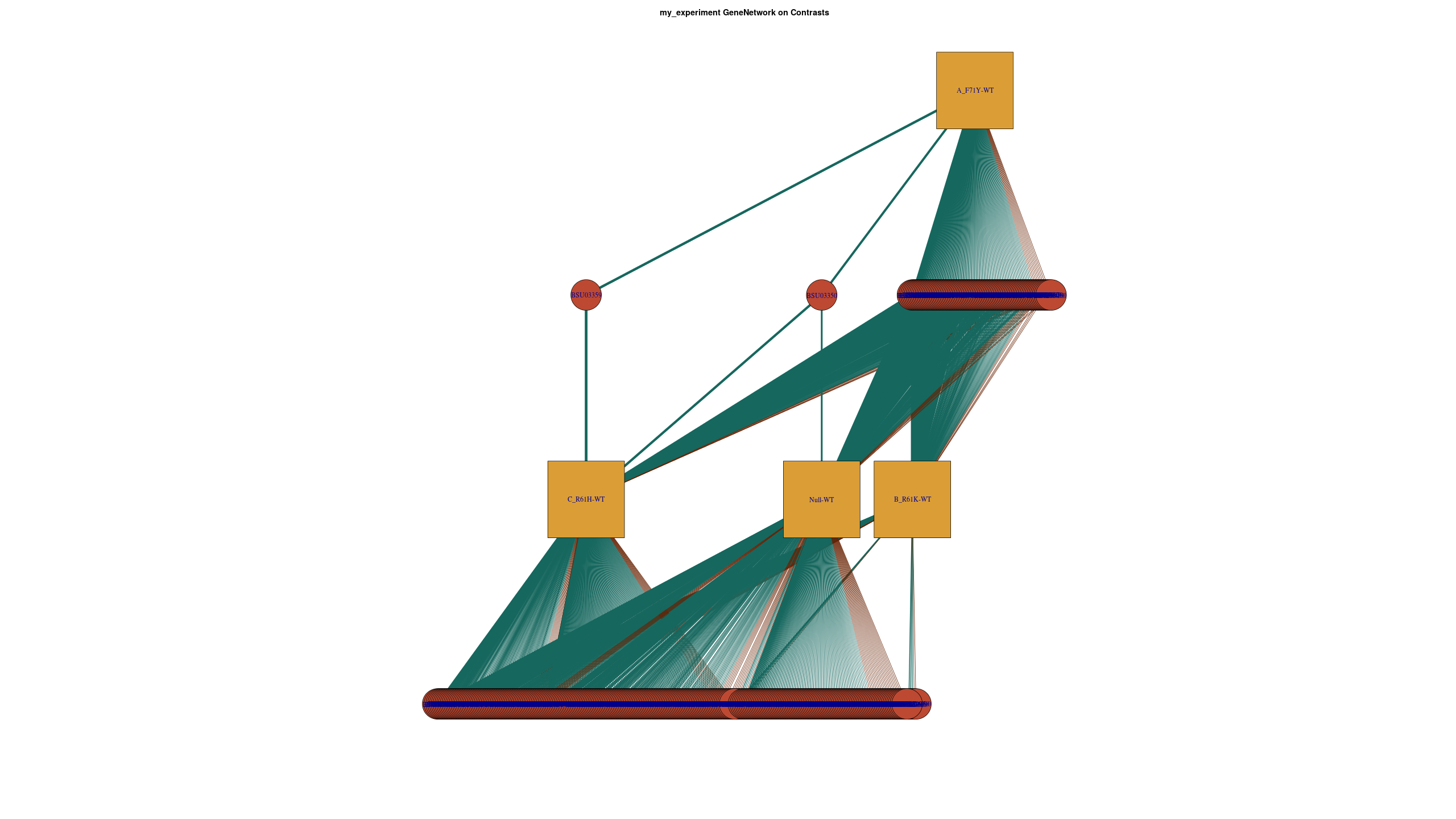

Supplement: Additional file 4: — Figures S4A and S4B; signal heatmaps of Class ‘Complex’ and Class ‘CodY’, respectively. (ZIP 1103 kb) [file 12864_2015_1834_MOESM4_ESM.zip › Figure_S2G.png]

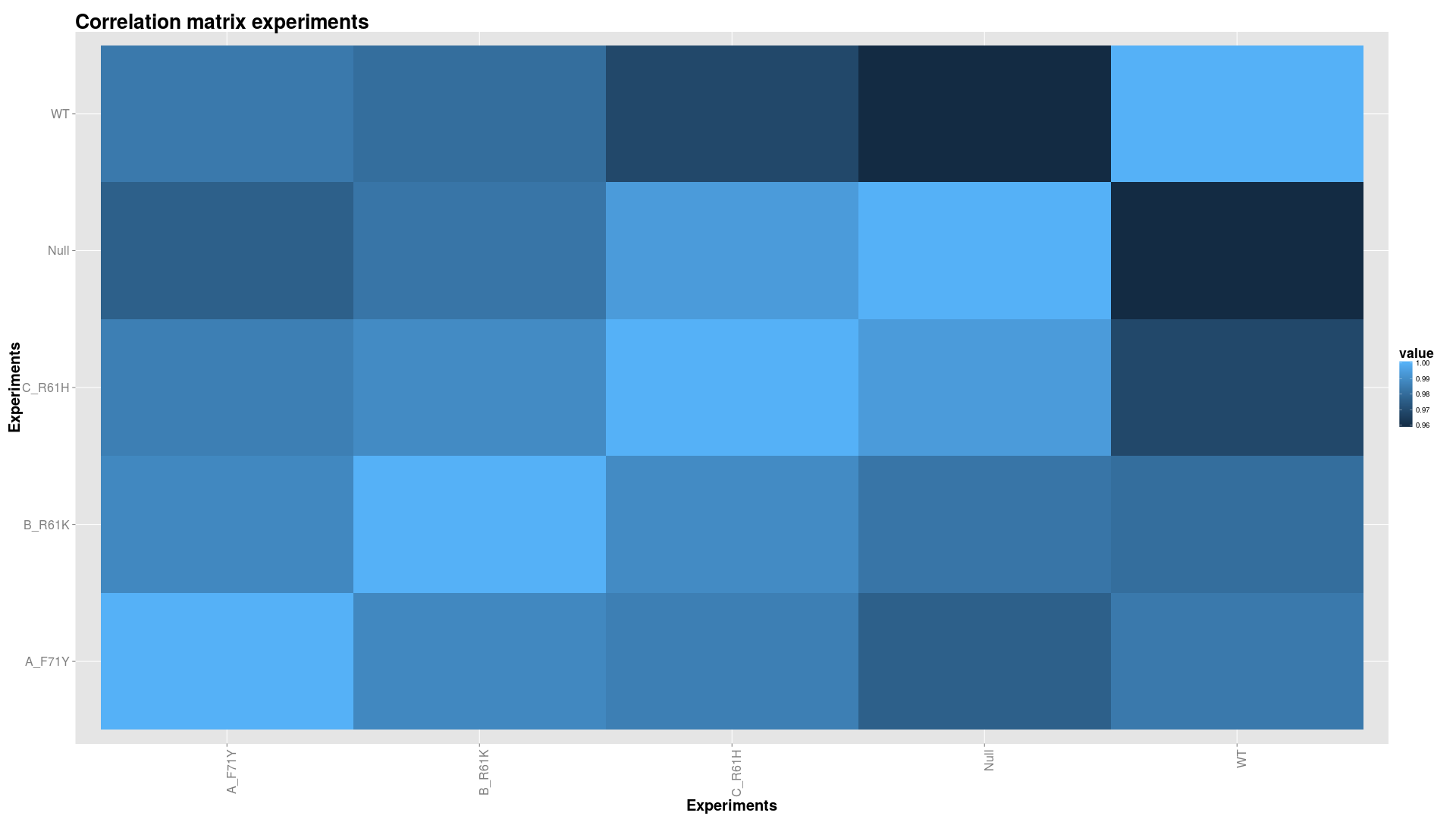

Supplement: Additional file 4: — Figures S4A and S4B; signal heatmaps of Class ‘Complex’ and Class ‘CodY’, respectively. (ZIP 1103 kb) [file 12864_2015_1834_MOESM4_ESM.zip › Figure_S2H.png]

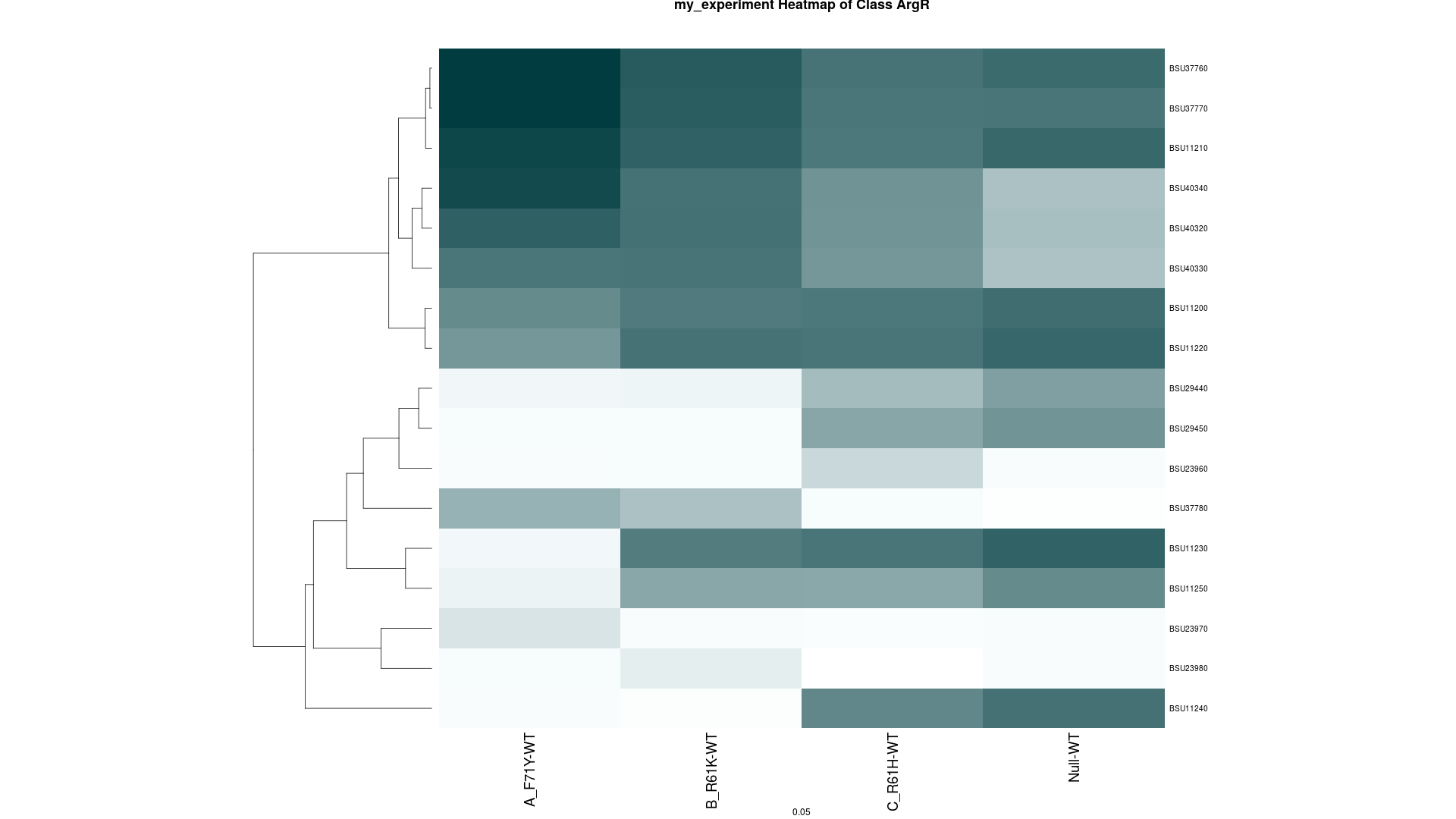

Supplement: Additional file 4: — Figures S4A and S4B; signal heatmaps of Class ‘Complex’ and Class ‘CodY’, respectively. (ZIP 1103 kb) [file 12864_2015_1834_MOESM4_ESM.zip › Figure_S2I.png]

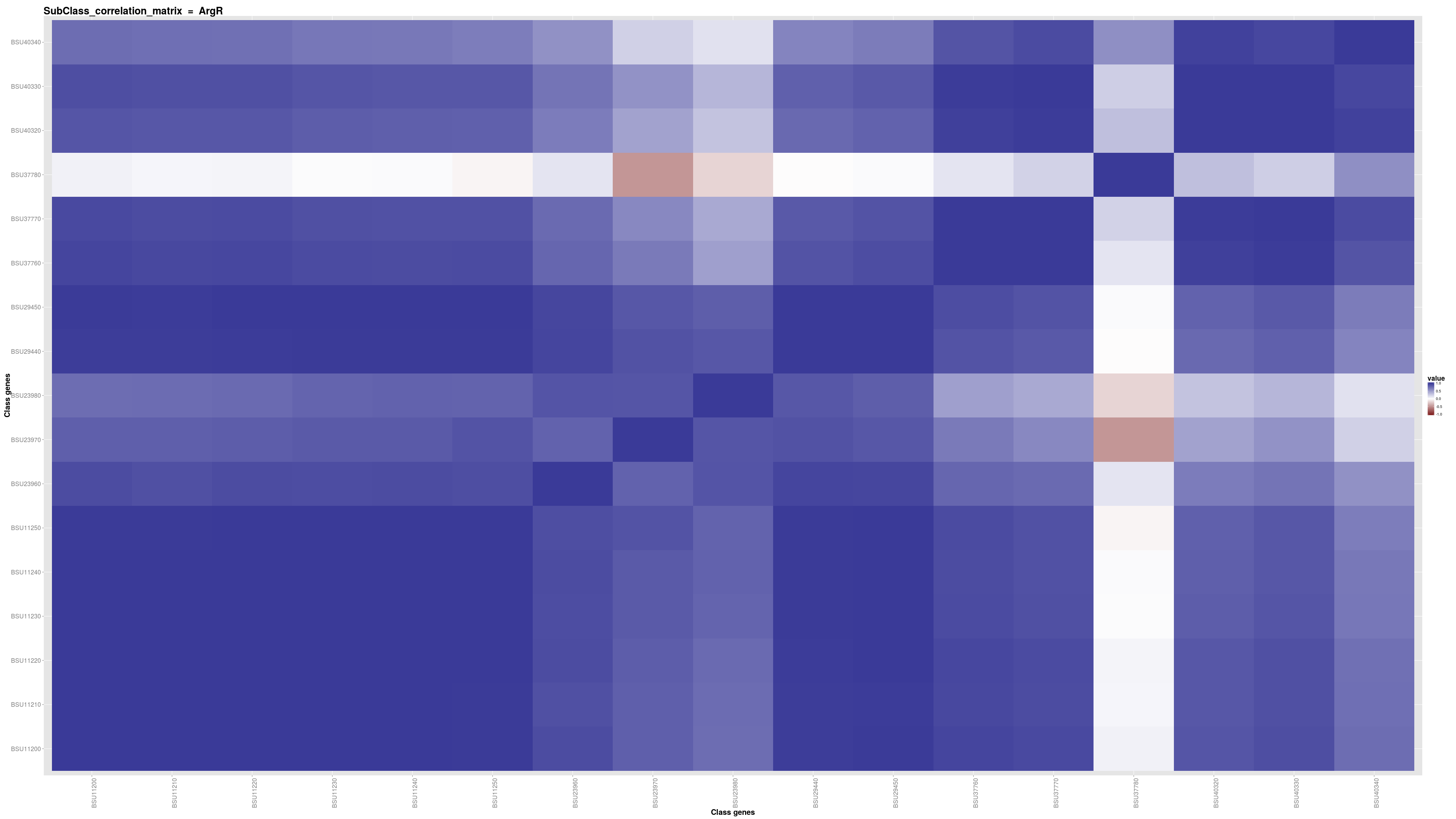

Supplement: Additional file 4: — Figures S4A and S4B; signal heatmaps of Class ‘Complex’ and Class ‘CodY’, respectively. (ZIP 1103 kb) [file 12864_2015_1834_MOESM4_ESM.zip › Figure_S2J.png]

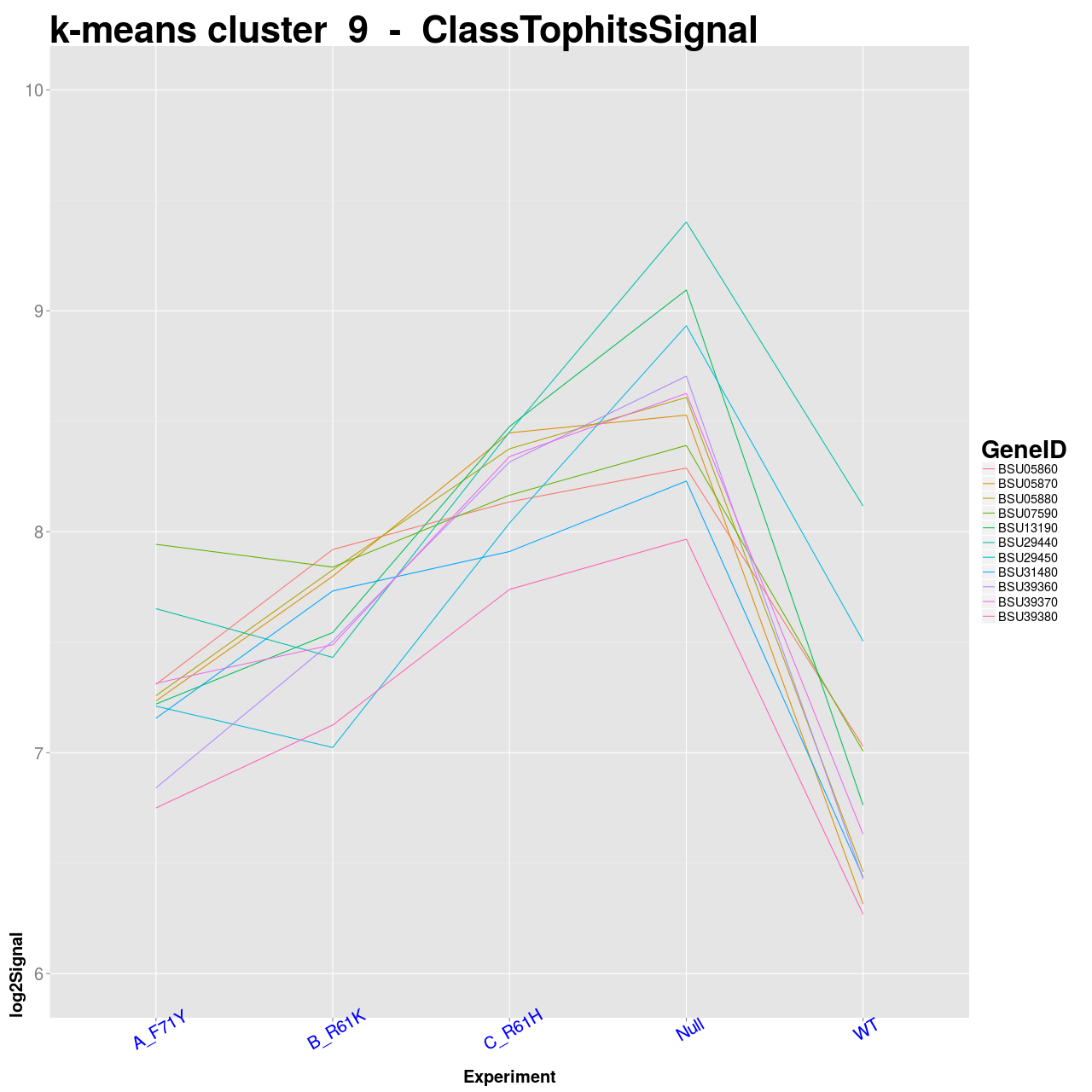

Supplement: Additional file 5: — T-REx main R-script. (PNG 173 kb) [file 12864_2015_1834_MOESM5_ESM.png]

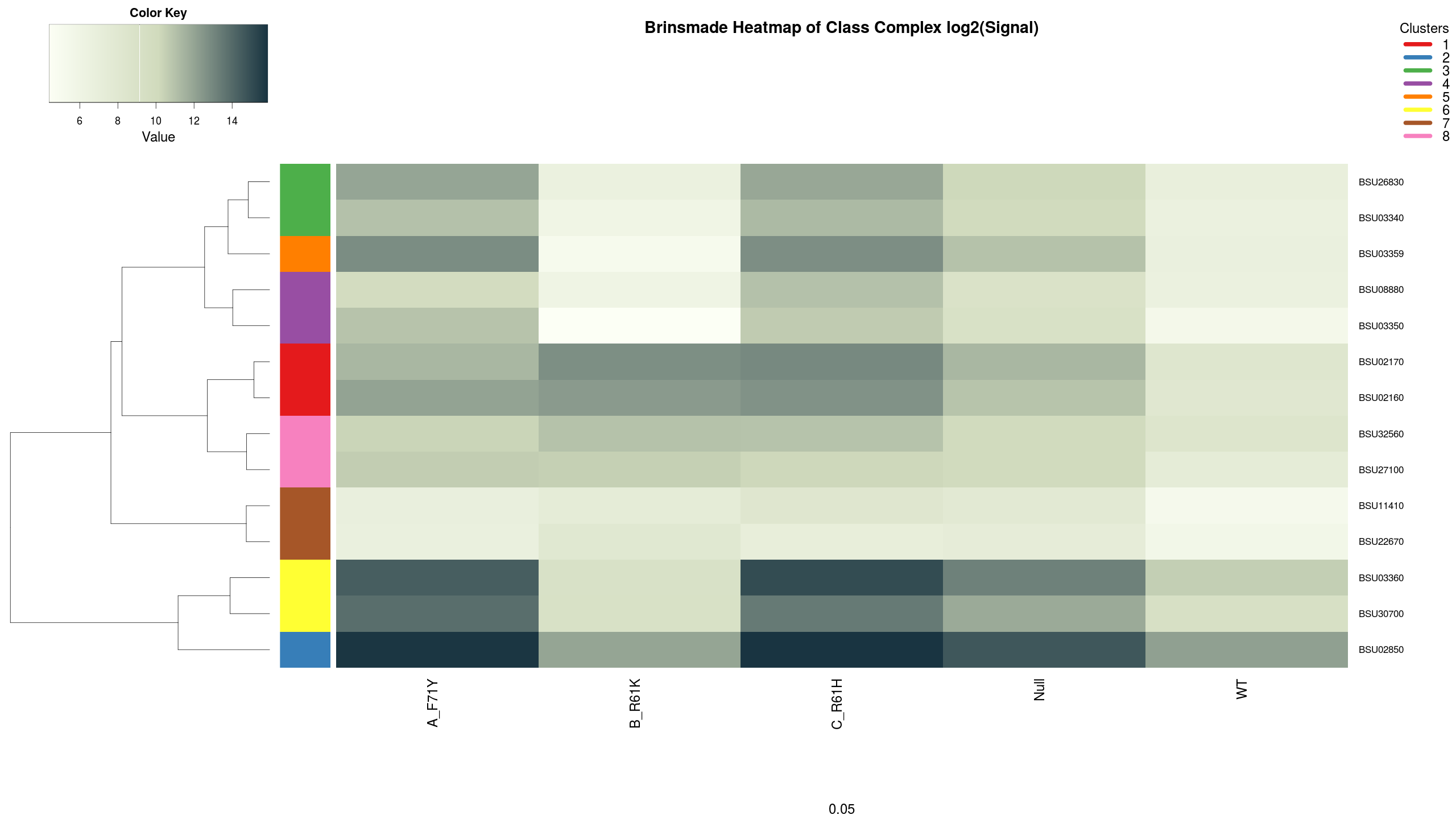

Supplement: Additional file 6: — T-REx R functions. (ZIP 179 kb) [file 12864_2015_1834_MOESM6_ESM.zip › Figure_S4A.png]

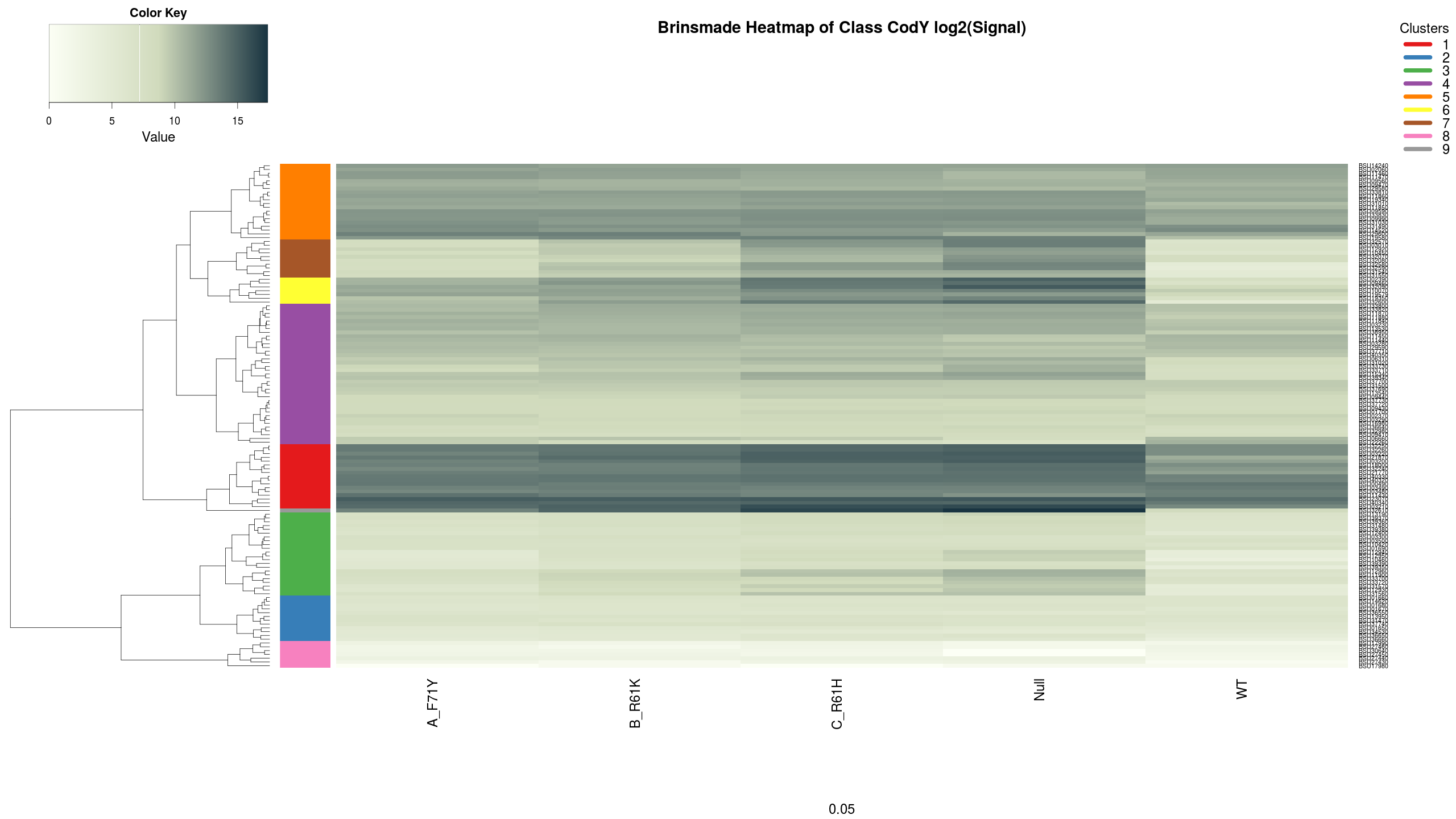

Supplement: Additional file 6: — T-REx R functions. (ZIP 179 kb) [file 12864_2015_1834_MOESM6_ESM.zip › Figure_S4B.png]
