# Supplementary material for: T-REx: Transcriptome analysis webserver for RNA-seq Expression data
Source: BMC Genomics. 2015 Sep 3;16(1):663. doi: 10.1186/s12864-015-1834-4 (PMC4558784; doi:10.1186/s12864-015-1834-4)
Supplement: Additional file 3: — Figure S3; k-means clustering of differentially expressed genes in the mutants. (ZIP 31925 kb) [file 12864_2015_1834_MOESM3_ESM.zip › Brinsmade.Contrasts_overview.html]

| Contrast | Total number of genes | Up regulated | Down regulated | Result table |
| --- | --- | --- | --- | --- |
