# Supplementary material for: T-REx: Transcriptome analysis webserver for RNA-seq Expression data
Source: BMC Genomics. 2015 Sep 3;16(1):663. doi: 10.1186/s12864-015-1834-4 (PMC4558784; doi:10.1186/s12864-015-1834-4)
Supplement: Additional file 3: — Figure S3; k-means clustering of differentially expressed genes in the mutants. (ZIP 31925 kb) [file 12864_2015_1834_MOESM3_ESM.zip › Brinsmade.Significant_Changed_Genes.1.A_F71Y-WT.txt.html]

| GeneID | logFC | logCPM | LR | pvalue | adj\_pvalue | Fold | minFDR |
| --- | --- | --- | --- | --- | --- | --- | --- |
| BSU03359 | 5.82 | 8.09 | 11.0 | 9.2e-04 | 4.7e-02 | 56.4 | 4.41 |
| BSU03350 | 5.31 | 6.12 | 12.4 | 4.3e-04 | 2.6e-02 | 39.7 | 5.26 |
| BSU32600 | 5.20 | 9.43 | 172.3 | 2.3e-39 | 9.6e-36 | 36.7 | 116.33 |
| BSU26830 | 4.95 | 7.25 | 14.2 | 1.7e-04 | 1.2e-02 | 30.9 | 6.41 |
| BSU32610 | 4.76 | 12.30 | 151.1 | 9.9e-35 | 2.1e-31 | 27.0 | 101.93 |
| BSU30700 | 4.45 | 8.90 | 15.0 | 1.1e-04 | 8.0e-03 | 21.9 | 6.96 |
| BSU02160 | 3.91 | 8.09 | 80.6 | 2.7e-19 | 1.0e-16 | 15.0 | 53.12 |
| BSU09460 | 3.87 | 9.44 | 96.2 | 1.0e-22 | 6.1e-20 | 14.6 | 63.83 |
| BSU02850 | 3.50 | 11.20 | 12.4 | 4.2e-04 | 2.6e-02 | 11.3 | 5.27 |
| BSU32590 | 3.41 | 7.88 | 42.1 | 8.7e-11 | 2.0e-08 | 10.6 | 25.57 |
| BSU28290 | 3.31 | 11.24 | 112.9 | 2.2e-26 | 3.1e-23 | 9.9 | 74.78 |
| BSU31540 | 3.27 | 6.49 | 59.4 | 1.3e-14 | 3.8e-12 | 9.6 | 37.93 |
| BSU32580 | 3.25 | 7.91 | 53.3 | 2.9e-13 | 8.0e-11 | 9.5 | 33.55 |
| BSU28300 | 3.21 | 9.51 | 94.5 | 2.5e-22 | 1.3e-19 | 9.3 | 62.74 |
| BSU27100 | 3.18 | 6.38 | 53.1 | 3.1e-13 | 8.1e-11 | 9.1 | 33.52 |
| BSU28280 | 3.13 | 11.01 | 108.0 | 2.7e-25 | 2.3e-22 | 8.8 | 71.91 |
| BSU28260 | 3.09 | 11.51 | 111.1 | 5.7e-26 | 5.9e-23 | 8.5 | 73.84 |
| BSU28250 | 3.06 | 9.68 | 93.5 | 4.1e-22 | 1.9e-19 | 8.4 | 62.19 |
| BSU28270 | 2.98 | 10.64 | 98.6 | 3.1e-23 | 2.2e-20 | 7.9 | 65.31 |
| BSU28310 | 2.93 | 10.87 | 88.2 | 6.0e-21 | 2.5e-18 | 7.6 | 58.47 |
| BSU02170 | 2.88 | 8.46 | 20.8 | 5.0e-06 | 5.1e-04 | 7.4 | 10.94 |
| BSU31550 | 2.87 | 6.12 | 45.4 | 1.6e-11 | 4.0e-09 | 7.3 | 27.91 |
| BSU02390 | 2.87 | 9.86 | 78.6 | 7.6e-19 | 2.7e-16 | 7.3 | 51.74 |
| BSU11360 | 2.76 | 6.42 | 41.2 | 1.4e-10 | 2.9e-08 | 6.8 | 25.02 |
| BSU19579 | 2.75 | 7.87 | 41.6 | 1.1e-10 | 2.4e-08 | 6.7 | 25.29 |
| BSU21870 | 2.72 | 10.75 | 73.5 | 9.9e-18 | 3.2e-15 | 6.6 | 48.17 |
| BSU32090 | 2.69 | 9.90 | 16.4 | 5.2e-05 | 4.2e-03 | 6.5 | 7.88 |
| BSU11390 | 2.52 | 6.72 | 40.4 | 2.0e-10 | 4.1e-08 | 5.7 | 24.55 |
| BSU11370 | 2.50 | 6.57 | 35.7 | 2.4e-09 | 4.1e-07 | 5.6 | 21.22 |
| BSU19350 | 2.47 | 8.26 | 37.6 | 8.5e-10 | 1.6e-07 | 5.5 | 22.56 |
| BSU31560 | 2.45 | 5.46 | 25.5 | 4.5e-07 | 6.0e-05 | 5.4 | 14.01 |
| BSU11400 | 2.38 | 6.58 | 36.3 | 1.7e-09 | 3.0e-07 | 5.2 | 21.65 |
| BSU04400 | 2.27 | 3.57 | 13.1 | 2.9e-04 | 1.8e-02 | 4.8 | 5.77 |
| BSU13940 | 2.26 | 6.11 | 22.9 | 1.7e-06 | 1.9e-04 | 4.8 | 12.35 |
| BSU39340 | 2.09 | 6.70 | 26.4 | 2.8e-07 | 3.9e-05 | 4.2 | 14.64 |
| BSU37480 | 2.07 | 4.66 | 27.9 | 1.2e-07 | 1.9e-05 | 4.2 | 15.66 |
| BSU12920 | 2.04 | 6.98 | 25.0 | 5.8e-07 | 7.3e-05 | 4.1 | 13.75 |
| BSU38770 | 2.02 | 5.03 | 17.1 | 3.6e-05 | 3.0e-03 | 4.1 | 8.39 |
| BSU15340 | 1.92 | 6.91 | 23.6 | 1.2e-06 | 1.3e-04 | 3.8 | 12.86 |
| BSU24090 | 1.92 | 8.53 | 33.2 | 8.1e-09 | 1.4e-06 | 3.8 | 19.49 |
| BSU19580 | 1.88 | 9.26 | 15.3 | 9.2e-05 | 6.9e-03 | 3.7 | 7.18 |
| BSU29600 | 1.83 | 8.91 | 21.9 | 2.9e-06 | 3.0e-04 | 3.5 | 11.68 |
| BSU19040 | 1.81 | 4.95 | 19.2 | 1.2e-05 | 1.1e-03 | 3.5 | 9.78 |
| BSU38960 | 1.80 | 8.25 | 24.9 | 5.9e-07 | 7.3e-05 | 3.5 | 13.75 |
| BSU21770 | 1.74 | 9.83 | 20.6 | 5.7e-06 | 5.6e-04 | 3.3 | 10.80 |
| BSU32560 | 1.69 | 6.76 | 15.9 | 6.5e-05 | 5.2e-03 | 3.2 | 7.60 |
| BSU32550 | 1.69 | 8.87 | 28.2 | 1.1e-07 | 1.8e-05 | 3.2 | 15.77 |
| BSU24070 | 1.62 | 8.47 | 24.1 | 9.1e-07 | 1.1e-04 | 3.1 | 13.16 |
| BSU32570 | 1.59 | 8.27 | 17.5 | 2.9e-05 | 2.6e-03 | 3.0 | 8.61 |
| BSU37460 | 1.59 | 9.42 | 12.4 | 4.4e-04 | 2.6e-02 | 3.0 | 5.26 |
| BSU02240 | 1.58 | 7.33 | 20.1 | 7.4e-06 | 7.2e-04 | 3.0 | 10.43 |
| BSU10230 | 1.54 | 4.84 | 13.2 | 2.9e-04 | 1.8e-02 | 2.9 | 5.79 |
| BSU10070 | 1.54 | 8.67 | 14.0 | 1.9e-04 | 1.2e-02 | 2.9 | 6.34 |
| BSU37770 | 1.52 | 8.97 | 23.8 | 1.0e-06 | 1.2e-04 | 2.9 | 13.01 |
| BSU11410 | 1.51 | 3.75 | 11.6 | 6.5e-04 | 3.5e-02 | 2.9 | 4.82 |
| BSU02250 | 1.51 | 5.48 | 15.5 | 8.4e-05 | 6.4e-03 | 2.8 | 7.29 |
| BSU03200 | 1.50 | 10.61 | 26.6 | 2.5e-07 | 3.5e-05 | 2.8 | 14.79 |
| BSU03210 | 1.46 | 11.70 | 27.7 | 1.4e-07 | 2.1e-05 | 2.8 | 15.56 |
| BSU24080 | 1.45 | 8.83 | 19.0 | 1.3e-05 | 1.2e-03 | 2.7 | 9.75 |
| BSU13580 | 1.42 | 7.32 | 14.5 | 1.4e-04 | 9.8e-03 | 2.7 | 6.67 |
| BSU03790 | 1.40 | 8.42 | 18.8 | 1.4e-05 | 1.3e-03 | 2.6 | 9.62 |
| BSU19060 | 1.39 | 6.00 | 13.6 | 2.3e-04 | 1.5e-02 | 2.6 | 6.05 |
| BSU08820 | 1.38 | 11.10 | 13.5 | 2.4e-04 | 1.6e-02 | 2.6 | 6.01 |
| BSU24050 | 1.34 | 8.78 | 16.3 | 5.5e-05 | 4.4e-03 | 2.5 | 7.81 |
| BSU37470 | 1.34 | 4.57 | 11.1 | 8.6e-04 | 4.5e-02 | 2.5 | 4.47 |
| BSU24030 | 1.34 | 9.27 | 19.1 | 1.2e-05 | 1.1e-03 | 2.5 | 9.77 |
| BSU18110 | 1.32 | 7.66 | 14.8 | 1.2e-04 | 8.6e-03 | 2.5 | 6.87 |
| BSU24060 | 1.32 | 8.93 | 15.7 | 7.5e-05 | 5.8e-03 | 2.5 | 7.44 |
| BSU24040 | 1.23 | 8.75 | 14.0 | 1.8e-04 | 1.2e-02 | 2.3 | 6.37 |
| BSU11870 | 1.22 | 7.37 | 11.5 | 6.8e-04 | 3.7e-02 | 2.3 | 4.78 |
| BSU13180 | 1.21 | 7.15 | 11.8 | 6.1e-04 | 3.4e-02 | 2.3 | 4.87 |
| BSU31010 | 1.17 | 7.90 | 12.3 | 4.4e-04 | 2.6e-02 | 2.3 | 5.26 |
| BSU39020 | -1.65 | 8.64 | 14.1 | 1.8e-04 | 1.2e-02 | -3.1 | 6.37 |
| BSU08440 | -1.70 | 5.02 | 11.6 | 6.4e-04 | 3.5e-02 | -3.2 | 4.82 |
| BSU31970 | -1.78 | 1.54 | 11.1 | 8.8e-04 | 4.5e-02 | -3.4 | 4.46 |
| BSU27090 | -1.79 | 5.20 | 17.1 | 3.5e-05 | 3.0e-03 | -3.5 | 8.39 |
| BSU12860 | -1.81 | 7.43 | 25.3 | 5.0e-07 | 6.5e-05 | -3.5 | 13.90 |
| BSU09680 | -1.84 | 7.24 | 22.6 | 2.0e-06 | 2.1e-04 | -3.6 | 12.21 |
| BSU03370 | -1.87 | 4.29 | 12.1 | 4.9e-04 | 2.9e-02 | -3.7 | 5.13 |
| BSU08450 | -2.21 | 3.88 | 11.7 | 6.1e-04 | 3.4e-02 | -4.6 | 4.87 |
| BSU08460 | -2.26 | 3.83 | 11.9 | 5.5e-04 | 3.1e-02 | -4.8 | 4.99 |
