# Supplementary material for: T-REx: Transcriptome analysis webserver for RNA-seq Expression data
Source: BMC Genomics. 2015 Sep 3;16(1):663. doi: 10.1186/s12864-015-1834-4 (PMC4558784; doi:10.1186/s12864-015-1834-4)
Supplement: Additional file 3: — Figure S3; k-means clustering of differentially expressed genes in the mutants. (ZIP 31925 kb) [file 12864_2015_1834_MOESM3_ESM.zip › Brinsmade.Significant_Changed_Genes.2.B_R61K-WT.txt.html]

| GeneID | logFC | logCPM | LR | pvalue | adj\_pvalue | Fold | minFDR |
| --- | --- | --- | --- | --- | --- | --- | --- |
| BSU32600 | 7.10 | 9.43 | 279.8 | 8.4e-63 | 3.5e-59 | 137.0 | 194.19 |
| BSU32610 | 6.49 | 12.30 | 239.6 | 4.8e-54 | 1.0e-50 | 89.8 | 166.09 |
| BSU32590 | 5.98 | 7.88 | 109.0 | 1.6e-25 | 4.6e-23 | 63.3 | 74.20 |
| BSU09460 | 5.63 | 9.44 | 174.4 | 7.9e-40 | 1.1e-36 | 49.4 | 119.44 |
| BSU32580 | 5.37 | 7.91 | 128.1 | 1.1e-29 | 4.5e-27 | 41.4 | 87.53 |
| BSU31540 | 4.78 | 6.49 | 115.8 | 5.3e-27 | 1.6e-24 | 27.5 | 79.07 |
| BSU02170 | 4.62 | 8.46 | 45.8 | 1.3e-11 | 1.6e-09 | 24.6 | 29.20 |
| BSU31550 | 4.45 | 6.12 | 99.5 | 1.9e-23 | 5.0e-21 | 21.9 | 67.44 |
| BSU02160 | 4.37 | 8.09 | 96.6 | 8.3e-23 | 2.0e-20 | 20.6 | 65.41 |
| BSU02390 | 4.20 | 9.86 | 151.2 | 9.3e-35 | 6.8e-32 | 18.4 | 103.53 |
| BSU31560 | 4.16 | 5.46 | 67.8 | 1.8e-16 | 3.1e-14 | 17.8 | 44.88 |
| BSU11360 | 4.14 | 6.42 | 83.7 | 5.6e-20 | 1.1e-17 | 17.6 | 56.31 |
| BSU19350 | 3.91 | 8.26 | 84.2 | 4.5e-20 | 9.3e-18 | 15.0 | 56.57 |
| BSU11390 | 3.86 | 6.72 | 86.4 | 1.5e-20 | 3.2e-18 | 14.6 | 58.09 |
| BSU31570 | 3.83 | 4.89 | 41.3 | 1.3e-10 | 1.6e-08 | 14.2 | 25.92 |
| BSU28280 | 3.82 | 11.01 | 151.1 | 9.8e-35 | 6.8e-32 | 14.2 | 103.53 |
| BSU11370 | 3.82 | 6.57 | 75.8 | 3.1e-18 | 5.5e-16 | 14.1 | 50.70 |
| BSU28250 | 3.79 | 9.68 | 134.1 | 5.2e-31 | 2.4e-28 | 13.8 | 91.73 |
| BSU28260 | 3.77 | 11.51 | 155.9 | 9.0e-36 | 9.4e-33 | 13.7 | 106.39 |
| BSU10460 | 3.75 | 4.25 | 30.0 | 4.3e-08 | 3.5e-06 | 13.5 | 18.11 |
| BSU10450 | 3.75 | 6.85 | 32.6 | 1.2e-08 | 1.1e-06 | 13.5 | 19.81 |
| BSU28270 | 3.74 | 10.64 | 144.7 | 2.5e-33 | 1.5e-30 | 13.3 | 99.10 |
| BSU28290 | 3.74 | 11.24 | 138.3 | 6.3e-32 | 3.3e-29 | 13.3 | 94.61 |
| BSU11400 | 3.73 | 6.58 | 80.8 | 2.5e-19 | 4.5e-17 | 13.2 | 54.29 |
| BSU28300 | 3.71 | 9.51 | 120.8 | 4.3e-28 | 1.5e-25 | 13.1 | 82.48 |
| BSU32570 | 3.68 | 8.27 | 83.0 | 8.4e-20 | 1.6e-17 | 12.8 | 55.81 |
| BSU28310 | 3.61 | 10.87 | 126.1 | 3.0e-29 | 1.1e-26 | 12.2 | 86.20 |
| BSU21870 | 3.58 | 10.75 | 118.5 | 1.3e-27 | 4.3e-25 | 11.9 | 80.95 |
| BSU12940 | 3.51 | 4.43 | 20.2 | 6.9e-06 | 3.9e-04 | 11.4 | 11.33 |
| BSU12930 | 3.32 | 4.80 | 22.6 | 2.0e-06 | 1.3e-04 | 10.0 | 12.87 |
| BSU12950 | 3.31 | 4.15 | 21.6 | 3.4e-06 | 2.1e-04 | 9.9 | 12.22 |
| BSU27100 | 3.26 | 6.38 | 55.4 | 9.9e-14 | 1.5e-11 | 9.6 | 35.98 |
| BSU32550 | 3.19 | 8.87 | 91.8 | 9.6e-22 | 2.2e-19 | 9.2 | 61.96 |
| BSU19580 | 3.07 | 9.26 | 37.2 | 1.0e-09 | 1.1e-07 | 8.4 | 23.05 |
| BSU37760 | 3.00 | 8.26 | 35.6 | 2.4e-09 | 2.5e-07 | 8.0 | 21.94 |
| BSU19579 | 2.96 | 7.87 | 47.3 | 6.2e-12 | 8.6e-10 | 7.8 | 30.11 |
| BSU11900 | 2.87 | 5.97 | 27.9 | 1.3e-07 | 9.5e-06 | 7.3 | 16.69 |
| BSU12920 | 2.86 | 6.98 | 46.8 | 7.8e-12 | 1.0e-09 | 7.3 | 29.83 |
| BSU10410 | 2.80 | 6.60 | 14.4 | 1.5e-04 | 6.1e-03 | 7.0 | 7.35 |
| BSU03010 | 2.78 | 8.23 | 40.8 | 1.7e-10 | 1.9e-08 | 6.9 | 25.63 |
| BSU32090 | 2.77 | 9.90 | 17.3 | 3.2e-05 | 1.6e-03 | 6.8 | 9.33 |
| BSU13940 | 2.74 | 6.11 | 32.5 | 1.2e-08 | 1.1e-06 | 6.7 | 19.81 |
| BSU38770 | 2.70 | 5.03 | 29.3 | 6.3e-08 | 5.1e-06 | 6.5 | 17.59 |
| BSU22670 | 2.68 | 3.61 | 16.0 | 6.4e-05 | 2.9e-03 | 6.4 | 8.43 |
| BSU32560 | 2.60 | 6.76 | 35.6 | 2.5e-09 | 2.5e-07 | 6.1 | 21.92 |
| BSU39350 | 2.59 | 3.30 | 14.4 | 1.5e-04 | 6.1e-03 | 6.0 | 7.35 |
| BSU39890 | 2.53 | 4.34 | 28.7 | 8.6e-08 | 6.8e-06 | 5.8 | 17.18 |
| BSU10070 | 2.52 | 8.67 | 34.9 | 3.4e-09 | 3.4e-07 | 5.7 | 21.49 |
| BSU38960 | 2.50 | 8.25 | 45.9 | 1.2e-11 | 1.6e-09 | 5.7 | 29.21 |
| BSU15340 | 2.35 | 6.91 | 34.2 | 5.0e-09 | 4.9e-07 | 5.1 | 20.97 |
| BSU03200 | 2.34 | 10.61 | 61.8 | 3.9e-15 | 6.3e-13 | 5.1 | 40.54 |
| BSU17820 | 2.33 | 1.69 | 14.6 | 1.3e-04 | 5.7e-03 | 5.0 | 7.46 |
| BSU11410 | 2.32 | 3.75 | 26.8 | 2.3e-07 | 1.6e-05 | 5.0 | 15.92 |
| BSU07530 | 2.31 | 3.70 | 18.5 | 1.7e-05 | 8.8e-04 | 5.0 | 10.15 |
| BSU39900 | 2.30 | 3.08 | 12.3 | 4.5e-04 | 1.6e-02 | 4.9 | 6.01 |
| BSU37770 | 2.26 | 8.97 | 50.0 | 1.6e-12 | 2.3e-10 | 4.8 | 32.04 |
| BSU39340 | 2.24 | 6.70 | 30.1 | 4.0e-08 | 3.4e-06 | 4.7 | 18.18 |
| BSU22640 | 2.24 | 5.33 | 22.6 | 2.0e-06 | 1.3e-04 | 4.7 | 12.87 |
| BSU22660 | 2.22 | 4.01 | 28.0 | 1.2e-07 | 9.4e-06 | 4.7 | 16.70 |
| BSU37480 | 2.21 | 4.66 | 31.8 | 1.7e-08 | 1.6e-06 | 4.6 | 19.27 |
| BSU29600 | 2.21 | 8.91 | 31.2 | 2.4e-08 | 2.1e-06 | 4.6 | 18.88 |
| BSU12960 | 2.19 | 5.86 | 27.1 | 1.9e-07 | 1.4e-05 | 4.6 | 16.14 |
| BSU03210 | 2.16 | 11.70 | 57.9 | 2.7e-14 | 4.2e-12 | 4.5 | 37.80 |
| BSU06310 | 2.08 | 6.64 | 20.5 | 5.9e-06 | 3.4e-04 | 4.2 | 11.51 |
| BSU02250 | 2.06 | 5.48 | 28.0 | 1.2e-07 | 9.3e-06 | 4.2 | 16.71 |
| BSU33730 | 2.06 | 6.15 | 27.4 | 1.7e-07 | 1.2e-05 | 4.2 | 16.33 |
| BSU03790 | 2.05 | 8.42 | 38.9 | 4.4e-10 | 5.0e-08 | 4.1 | 24.26 |
| BSU02240 | 2.00 | 7.33 | 31.3 | 2.2e-08 | 2.0e-06 | 4.0 | 18.94 |
| BSU22650 | 2.00 | 4.06 | 18.3 | 1.9e-05 | 9.7e-04 | 4.0 | 10.00 |
| BSU10300 | 2.00 | 3.25 | 19.4 | 1.0e-05 | 5.5e-04 | 4.0 | 10.82 |
| BSU14320 | 1.97 | 3.98 | 20.3 | 6.5e-06 | 3.7e-04 | 3.9 | 11.39 |
| BSU19040 | 1.96 | 4.95 | 22.4 | 2.2e-06 | 1.4e-04 | 3.9 | 12.81 |
| BSU33710 | 1.96 | 5.98 | 19.6 | 9.4e-06 | 5.1e-04 | 3.9 | 10.94 |
| BSU36660 | 1.95 | 1.81 | 12.7 | 3.6e-04 | 1.3e-02 | 3.9 | 6.26 |
| BSU22680 | 1.89 | 4.75 | 13.0 | 3.0e-04 | 1.1e-02 | 3.7 | 6.45 |
| BSU38090 | 1.85 | 6.27 | 21.4 | 3.7e-06 | 2.3e-04 | 3.6 | 12.11 |
| BSU12400 | 1.85 | 3.87 | 10.6 | 1.2e-03 | 3.6e-02 | 3.6 | 4.78 |
| BSU24090 | 1.83 | 8.53 | 30.3 | 3.8e-08 | 3.2e-06 | 3.5 | 18.24 |
| BSU21770 | 1.81 | 9.83 | 22.2 | 2.4e-06 | 1.5e-04 | 3.5 | 12.69 |
| BSU02400 | 1.73 | 7.79 | 14.4 | 1.5e-04 | 6.2e-03 | 3.3 | 7.34 |
| BSU10230 | 1.72 | 4.84 | 16.3 | 5.4e-05 | 2.5e-03 | 3.3 | 8.66 |
| BSU11010 | 1.71 | 5.16 | 12.5 | 4.1e-04 | 1.5e-02 | 3.3 | 6.11 |
| BSU13180 | 1.70 | 7.15 | 22.5 | 2.1e-06 | 1.4e-04 | 3.2 | 12.81 |
| BSU24080 | 1.69 | 8.83 | 25.4 | 4.7e-07 | 3.3e-05 | 3.2 | 14.90 |
| BSU37680 | 1.68 | 5.68 | 16.3 | 5.3e-05 | 2.5e-03 | 3.2 | 8.66 |
| BSU11880 | 1.65 | 7.22 | 13.5 | 2.4e-04 | 9.3e-03 | 3.1 | 6.75 |
| BSU10390 | 1.65 | 2.12 | 9.9 | 1.7e-03 | 5.0e-02 | 3.1 | 4.33 |
| BSU05870 | 1.64 | 4.27 | 15.9 | 6.6e-05 | 2.9e-03 | 3.1 | 8.42 |
| BSU19060 | 1.63 | 6.00 | 18.3 | 1.9e-05 | 9.7e-04 | 3.1 | 10.00 |
| BSU37740 | 1.60 | 3.15 | 11.0 | 9.3e-04 | 3.0e-02 | 3.0 | 5.05 |
| BSU24070 | 1.59 | 8.47 | 23.4 | 1.3e-06 | 9.0e-05 | 3.0 | 13.44 |
| BSU13580 | 1.59 | 7.32 | 18.0 | 2.2e-05 | 1.1e-03 | 3.0 | 9.81 |
| BSU18410 | 1.57 | 2.63 | 12.1 | 5.2e-04 | 1.8e-02 | 3.0 | 5.83 |
| BSU40021 | 1.56 | 3.85 | 13.9 | 1.9e-04 | 7.8e-03 | 3.0 | 7.01 |
| BSU18110 | 1.56 | 7.66 | 20.2 | 7.0e-06 | 3.9e-04 | 2.9 | 11.33 |
| BSU34190 | 1.54 | 8.32 | 10.4 | 1.2e-03 | 3.9e-02 | 2.9 | 4.70 |
| BSU26650 | 1.54 | 3.90 | 12.0 | 5.2e-04 | 1.8e-02 | 2.9 | 5.83 |
| BSU05880 | 1.53 | 4.29 | 12.9 | 3.3e-04 | 1.2e-02 | 2.9 | 6.34 |
| BSU29680 | 1.52 | 7.11 | 17.0 | 3.7e-05 | 1.8e-03 | 2.9 | 9.14 |
| BSU11870 | 1.50 | 7.37 | 17.4 | 3.1e-05 | 1.5e-03 | 2.8 | 9.37 |
| BSU03220 | 1.50 | 10.80 | 14.1 | 1.8e-04 | 7.1e-03 | 2.8 | 7.14 |
| BSU24060 | 1.49 | 8.93 | 19.7 | 9.1e-06 | 5.0e-04 | 2.8 | 10.97 |
| BSU18550 | 1.46 | 4.33 | 12.7 | 3.7e-04 | 1.3e-02 | 2.8 | 6.22 |
| BSU19050 | 1.44 | 5.71 | 15.2 | 9.7e-05 | 4.2e-03 | 2.7 | 7.90 |
| BSU24050 | 1.42 | 8.78 | 18.4 | 1.8e-05 | 9.4e-04 | 2.7 | 10.06 |
| BSU37460 | 1.42 | 9.42 | 10.0 | 1.6e-03 | 4.7e-02 | 2.7 | 4.40 |
| BSU40180 | 1.41 | 6.74 | 10.4 | 1.3e-03 | 3.9e-02 | 2.7 | 4.69 |
| BSU13560 | 1.41 | 5.43 | 13.6 | 2.3e-04 | 9.0e-03 | 2.6 | 6.80 |
| BSU08820 | 1.40 | 11.10 | 13.8 | 2.1e-04 | 8.2e-03 | 2.6 | 6.93 |
| BSU24030 | 1.39 | 9.27 | 20.7 | 5.5e-06 | 3.2e-04 | 2.6 | 11.60 |
| BSU32240 | 1.38 | 9.96 | 11.9 | 5.6e-04 | 1.9e-02 | 2.6 | 5.73 |
| BSU31020 | 1.38 | 6.22 | 11.8 | 5.8e-04 | 1.9e-02 | 2.6 | 5.70 |
| BSU07840 | 1.38 | 8.83 | 17.2 | 3.4e-05 | 1.6e-03 | 2.6 | 9.26 |
| BSU37470 | 1.37 | 4.57 | 11.6 | 6.6e-04 | 2.2e-02 | 2.6 | 5.52 |
| BSU32260 | 1.36 | 10.51 | 22.2 | 2.4e-06 | 1.5e-04 | 2.6 | 12.69 |
| BSU32250 | 1.35 | 10.56 | 21.2 | 4.1e-06 | 2.4e-04 | 2.6 | 12.00 |
| BSU31000 | 1.34 | 6.22 | 13.5 | 2.4e-04 | 9.3e-03 | 2.5 | 6.75 |
| BSU24040 | 1.32 | 8.75 | 16.0 | 6.5e-05 | 2.9e-03 | 2.5 | 8.43 |
| BSU31010 | 1.30 | 7.90 | 15.2 | 9.7e-05 | 4.2e-03 | 2.5 | 7.90 |
| BSU38950 | 1.27 | 6.84 | 10.0 | 1.6e-03 | 4.7e-02 | 2.4 | 4.40 |
| BSU32070 | 1.26 | 6.96 | 9.8 | 1.7e-03 | 5.0e-02 | 2.4 | 4.33 |
| BSU38550 | 1.18 | 10.15 | 16.3 | 5.3e-05 | 2.5e-03 | 2.3 | 8.66 |
| BSU33830 | 1.16 | 8.67 | 13.6 | 2.3e-04 | 9.0e-03 | 2.2 | 6.79 |
| BSU13850 | 1.14 | 9.94 | 9.9 | 1.7e-03 | 5.0e-02 | 2.2 | 4.33 |
| BSU33810 | 1.14 | 8.17 | 11.1 | 8.8e-04 | 2.9e-02 | 2.2 | 5.13 |
| BSU06170 | 1.05 | 8.39 | 10.8 | 1.0e-03 | 3.3e-02 | 2.1 | 4.94 |
| BSU29470 | -1.07 | 9.60 | 12.9 | 3.3e-04 | 1.2e-02 | -2.1 | 6.34 |
| BSU37660 | -1.15 | 8.98 | 12.7 | 3.6e-04 | 1.3e-02 | -2.2 | 6.25 |
| BSU35960 | -1.22 | 4.58 | 10.4 | 1.2e-03 | 3.9e-02 | -2.3 | 4.70 |
| BSU35940 | -1.27 | 4.78 | 10.5 | 1.2e-03 | 3.7e-02 | -2.4 | 4.75 |
| BSU38270 | -1.33 | 5.71 | 11.3 | 7.9e-04 | 2.6e-02 | -2.5 | 5.26 |
| BSU27090 | -1.53 | 5.20 | 12.6 | 3.9e-04 | 1.4e-02 | -2.9 | 6.15 |
| BSU39020 | -1.55 | 8.64 | 12.4 | 4.2e-04 | 1.5e-02 | -2.9 | 6.07 |
| BSU07150 | -1.82 | 3.75 | 13.4 | 2.5e-04 | 9.3e-03 | -3.5 | 6.75 |
| BSU01630 | -1.82 | 2.08 | 10.9 | 9.6e-04 | 3.1e-02 | -3.5 | 5.02 |
| BSU08440 | -1.91 | 5.02 | 14.5 | 1.4e-04 | 5.8e-03 | -3.8 | 7.42 |
| BSU24950 | -2.17 | 3.26 | 10.2 | 1.4e-03 | 4.2e-02 | -4.5 | 4.56 |
| BSU22260 | -2.18 | 5.63 | 19.5 | 1.0e-05 | 5.4e-04 | -4.5 | 10.87 |
| BSU09680 | -2.39 | 7.24 | 36.8 | 1.3e-09 | 1.4e-07 | -5.2 | 22.75 |
| BSU12860 | -2.41 | 7.43 | 42.9 | 5.8e-11 | 7.1e-09 | -5.3 | 27.06 |
| BSU24990 | -3.07 | 5.00 | 15.3 | 9.2e-05 | 4.0e-03 | -8.4 | 7.95 |
