# Supplementary material for: T-REx: Transcriptome analysis webserver for RNA-seq Expression data
Source: BMC Genomics. 2015 Sep 3;16(1):663. doi: 10.1186/s12864-015-1834-4 (PMC4558784; doi:10.1186/s12864-015-1834-4)
Supplement: Additional file 3: — Figure S3; k-means clustering of differentially expressed genes in the mutants. (ZIP 31925 kb) [file 12864_2015_1834_MOESM3_ESM.zip › Brinsmade.Significant_Changed_Genes.3.C_R61H-WT.txt.html]

| GeneID | logFC | logCPM | LR | pvalue | adj\_pvalue | Fold | minFDR |
| --- | --- | --- | --- | --- | --- | --- | --- |
| BSU32600 | 8.76 | 9.43 | 377.0 | 5.6e-84 | 2.3e-80 | 433.4 | 264.53 |
| BSU32610 | 8.10 | 12.30 | 324.3 | 1.7e-72 | 3.6e-69 | 273.5 | 227.37 |
| BSU32590 | 7.73 | 7.88 | 158.9 | 2.0e-36 | 5.2e-34 | 212.8 | 110.57 |
| BSU32580 | 7.47 | 7.91 | 211.7 | 5.8e-48 | 4.0e-45 | 177.8 | 147.47 |
| BSU09460 | 7.12 | 9.44 | 245.0 | 3.2e-55 | 3.3e-52 | 138.7 | 171.01 |
| BSU03359 | 6.36 | 8.09 | 12.5 | 4.1e-04 | 8.9e-03 | 82.2 | 6.81 |
| BSU02390 | 6.29 | 9.86 | 281.2 | 4.2e-63 | 5.9e-60 | 78.3 | 196.77 |
| BSU31540 | 6.15 | 6.49 | 172.7 | 1.9e-39 | 6.0e-37 | 70.8 | 120.33 |
| BSU32570 | 5.93 | 8.27 | 177.9 | 1.4e-40 | 4.9e-38 | 61.0 | 123.94 |
| BSU31550 | 5.76 | 6.12 | 151.1 | 9.8e-35 | 2.4e-32 | 54.1 | 105.04 |
| BSU03350 | 5.61 | 6.12 | 13.4 | 2.5e-04 | 5.7e-03 | 48.9 | 7.45 |
| BSU31560 | 5.49 | 5.46 | 107.6 | 3.3e-25 | 5.3e-23 | 45.0 | 74.01 |
| BSU03010 | 5.47 | 8.23 | 126.2 | 2.8e-29 | 5.7e-27 | 44.4 | 87.17 |
| BSU26830 | 5.41 | 7.25 | 16.2 | 5.7e-05 | 1.6e-03 | 42.7 | 9.29 |
| BSU19350 | 5.28 | 8.26 | 135.8 | 2.2e-31 | 4.8e-29 | 39.0 | 94.07 |
| BSU32550 | 5.16 | 8.87 | 202.2 | 6.9e-46 | 3.6e-43 | 35.9 | 141.00 |
| BSU10450 | 5.09 | 6.85 | 53.1 | 3.1e-13 | 3.0e-11 | 34.0 | 34.95 |
| BSU10460 | 5.05 | 4.25 | 50.0 | 1.5e-12 | 1.4e-10 | 33.1 | 32.73 |
| BSU31570 | 5.05 | 4.89 | 65.5 | 5.9e-16 | 6.9e-14 | 33.1 | 43.72 |
| BSU03340 | 5.02 | 6.61 | 11.7 | 6.4e-04 | 1.3e-02 | 32.5 | 6.25 |
| BSU32090 | 4.92 | 9.90 | 44.7 | 2.4e-11 | 1.8e-09 | 30.2 | 29.06 |
| BSU03360 | 4.88 | 10.22 | 9.8 | 1.7e-03 | 3.2e-02 | 29.5 | 4.96 |
| BSU08880 | 4.85 | 6.03 | 13.6 | 2.3e-04 | 5.2e-03 | 28.9 | 7.58 |
| BSU11360 | 4.84 | 6.42 | 107.8 | 3.0e-25 | 5.0e-23 | 28.6 | 74.08 |
| BSU02170 | 4.78 | 8.46 | 48.4 | 3.5e-12 | 3.0e-10 | 27.5 | 31.62 |
| BSU28260 | 4.62 | 11.51 | 215.8 | 7.5e-49 | 6.3e-46 | 24.6 | 150.16 |
| BSU28280 | 4.60 | 11.01 | 203.4 | 3.7e-46 | 2.2e-43 | 24.3 | 141.69 |
| BSU28290 | 4.60 | 11.24 | 193.3 | 6.1e-44 | 2.6e-41 | 24.2 | 134.84 |
| BSU11390 | 4.58 | 6.72 | 114.1 | 1.2e-26 | 2.4e-24 | 23.9 | 78.44 |
| BSU11370 | 4.57 | 6.57 | 101.7 | 6.4e-24 | 9.6e-22 | 23.7 | 69.82 |
| BSU02160 | 4.56 | 8.09 | 103.6 | 2.5e-24 | 3.9e-22 | 23.6 | 71.12 |
| BSU28250 | 4.54 | 9.68 | 179.6 | 5.8e-41 | 2.2e-38 | 23.3 | 125.09 |
| BSU11400 | 4.54 | 6.58 | 112.0 | 3.6e-26 | 6.3e-24 | 23.2 | 77.06 |
| BSU28270 | 4.52 | 10.64 | 196.8 | 1.0e-44 | 4.8e-42 | 22.9 | 137.26 |
| BSU28300 | 4.50 | 9.51 | 165.0 | 9.0e-38 | 2.5e-35 | 22.6 | 114.95 |
| BSU30700 | 4.44 | 8.90 | 14.9 | 1.1e-04 | 2.9e-03 | 21.7 | 8.44 |
| BSU12920 | 4.42 | 6.98 | 98.5 | 3.2e-23 | 4.6e-21 | 21.4 | 67.56 |
| BSU28310 | 4.34 | 10.87 | 170.7 | 5.2e-39 | 1.5e-36 | 20.3 | 118.96 |
| BSU12940 | 4.34 | 4.43 | 29.1 | 7.0e-08 | 3.8e-06 | 20.3 | 18.00 |
| BSU12930 | 4.30 | 4.80 | 35.2 | 3.0e-09 | 2.1e-07 | 19.7 | 22.20 |
| BSU13940 | 4.24 | 6.11 | 69.9 | 6.2e-17 | 7.8e-15 | 18.9 | 46.87 |
| BSU21870 | 4.12 | 10.75 | 149.6 | 2.1e-34 | 4.8e-32 | 17.4 | 104.04 |
| BSU10410 | 4.03 | 6.60 | 26.9 | 2.1e-07 | 1.0e-05 | 16.3 | 16.57 |
| BSU12950 | 3.92 | 4.15 | 28.9 | 7.5e-08 | 4.0e-06 | 15.1 | 17.93 |
| BSU02850 | 3.91 | 11.20 | 14.9 | 1.1e-04 | 2.9e-03 | 15.0 | 8.45 |
| BSU11900 | 3.90 | 5.97 | 47.6 | 5.3e-12 | 4.4e-10 | 14.9 | 31.08 |
| BSU39890 | 3.75 | 4.34 | 61.0 | 5.7e-15 | 6.2e-13 | 13.5 | 40.55 |
| BSU37760 | 3.60 | 8.26 | 48.7 | 3.0e-12 | 2.6e-10 | 12.2 | 31.82 |
| BSU39350 | 3.60 | 3.30 | 26.4 | 2.7e-07 | 1.2e-05 | 12.1 | 16.29 |
| BSU39900 | 3.46 | 3.08 | 27.4 | 1.6e-07 | 8.0e-06 | 11.0 | 16.94 |
| BSU19580 | 3.46 | 9.26 | 45.6 | 1.5e-11 | 1.1e-09 | 11.0 | 29.72 |
| BSU10070 | 3.46 | 8.67 | 61.0 | 5.8e-15 | 6.2e-13 | 11.0 | 40.55 |
| BSU15340 | 3.45 | 6.91 | 68.1 | 1.5e-16 | 1.8e-14 | 11.0 | 45.62 |
| BSU07530 | 3.41 | 3.70 | 38.6 | 5.2e-10 | 3.7e-08 | 10.6 | 24.67 |
| BSU19579 | 3.40 | 7.87 | 60.0 | 9.4e-15 | 9.8e-13 | 10.5 | 39.89 |
| BSU03200 | 3.30 | 10.61 | 113.8 | 1.5e-26 | 2.8e-24 | 9.9 | 78.25 |
| BSU39340 | 3.20 | 6.70 | 57.2 | 4.0e-14 | 4.0e-12 | 9.2 | 37.85 |
| BSU12960 | 3.19 | 5.86 | 53.9 | 2.2e-13 | 2.1e-11 | 9.1 | 35.44 |
| BSU38960 | 3.17 | 8.25 | 69.6 | 7.4e-17 | 9.1e-15 | 9.0 | 46.65 |
| BSU03210 | 3.14 | 11.70 | 113.4 | 1.8e-26 | 3.2e-24 | 8.8 | 78.04 |
| BSU11230 | 3.12 | 2.55 | 14.0 | 1.9e-04 | 4.4e-03 | 8.7 | 7.83 |
| BSU11410 | 3.07 | 3.75 | 45.8 | 1.3e-11 | 1.1e-09 | 8.4 | 29.81 |
| BSU12400 | 3.03 | 3.87 | 26.7 | 2.4e-07 | 1.1e-05 | 8.2 | 16.45 |
| BSU11220 | 2.95 | 3.69 | 14.3 | 1.6e-04 | 3.8e-03 | 7.7 | 8.03 |
| BSU38090 | 2.94 | 6.27 | 50.2 | 1.4e-12 | 1.3e-10 | 7.7 | 32.86 |
| BSU27100 | 2.92 | 6.38 | 45.6 | 1.4e-11 | 1.1e-09 | 7.6 | 29.73 |
| BSU21190 | 2.88 | 0.62 | 19.1 | 1.2e-05 | 4.0e-04 | 7.4 | 11.29 |
| BSU38930 | 2.86 | 1.98 | 16.0 | 6.4e-05 | 1.7e-03 | 7.2 | 9.19 |
| BSU38770 | 2.85 | 5.03 | 32.3 | 1.3e-08 | 7.6e-07 | 7.2 | 20.33 |
| BSU37770 | 2.82 | 8.97 | 74.6 | 5.8e-18 | 7.6e-16 | 7.0 | 50.23 |
| BSU17820 | 2.71 | 1.69 | 20.0 | 7.8e-06 | 2.7e-04 | 6.6 | 11.87 |
| BSU03790 | 2.70 | 8.42 | 64.2 | 1.1e-15 | 1.3e-13 | 6.5 | 42.84 |
| BSU02250 | 2.68 | 5.48 | 45.9 | 1.2e-11 | 1.0e-09 | 6.4 | 29.88 |
| BSU03020 | 2.66 | 6.88 | 27.5 | 1.6e-07 | 7.9e-06 | 6.3 | 16.95 |
| BSU02240 | 2.61 | 7.33 | 51.3 | 7.9e-13 | 7.5e-11 | 6.1 | 33.64 |
| BSU06310 | 2.61 | 6.64 | 31.0 | 2.6e-08 | 1.5e-06 | 6.1 | 19.32 |
| BSU32070 | 2.58 | 6.96 | 38.0 | 6.9e-10 | 4.9e-08 | 6.0 | 24.28 |
| BSU32080 | 2.57 | 7.17 | 21.1 | 4.5e-06 | 1.7e-04 | 6.0 | 12.55 |
| BSU08830 | 2.55 | 0.41 | 9.3 | 2.3e-03 | 4.1e-02 | 5.9 | 4.62 |
| BSU11010 | 2.54 | 5.16 | 26.4 | 2.8e-07 | 1.3e-05 | 5.8 | 16.27 |
| BSU13180 | 2.54 | 7.15 | 47.6 | 5.2e-12 | 4.4e-10 | 5.8 | 31.08 |
| BSU32560 | 2.50 | 6.76 | 33.0 | 9.2e-09 | 5.8e-07 | 5.6 | 20.71 |
| BSU12980 | 2.49 | 4.28 | 21.7 | 3.3e-06 | 1.3e-04 | 5.6 | 12.96 |
| BSU36660 | 2.49 | 1.81 | 21.2 | 4.1e-06 | 1.6e-04 | 5.6 | 12.65 |
| BSU19040 | 2.47 | 4.95 | 34.6 | 4.0e-09 | 2.7e-07 | 5.5 | 21.81 |
| BSU03220 | 2.42 | 10.80 | 34.5 | 4.2e-09 | 2.8e-07 | 5.4 | 21.77 |
| BSU11200 | 2.41 | 3.86 | 12.9 | 3.2e-04 | 7.1e-03 | 5.3 | 7.14 |
| BSU11210 | 2.40 | 3.22 | 13.1 | 3.0e-04 | 6.7e-03 | 5.3 | 7.22 |
| BSU18550 | 2.39 | 4.33 | 32.8 | 1.0e-08 | 6.5e-07 | 5.2 | 20.56 |
| BSU12970 | 2.38 | 4.04 | 29.5 | 5.6e-08 | 3.1e-06 | 5.2 | 18.30 |
| BSU02400 | 2.33 | 7.79 | 25.1 | 5.4e-07 | 2.3e-05 | 5.0 | 15.43 |
| BSU18540 | 2.32 | 4.23 | 26.4 | 2.8e-07 | 1.3e-05 | 5.0 | 16.28 |
| BSU37480 | 2.31 | 4.66 | 34.5 | 4.3e-09 | 2.8e-07 | 5.0 | 21.76 |
| BSU17130 | 2.31 | 1.88 | 18.7 | 1.5e-05 | 4.7e-04 | 5.0 | 11.05 |
| BSU17140 | 2.31 | 4.08 | 27.9 | 1.3e-07 | 6.6e-06 | 4.9 | 17.21 |
| BSU05870 | 2.28 | 4.27 | 30.1 | 4.2e-08 | 2.4e-06 | 4.8 | 18.68 |
| BSU12990 | 2.26 | 4.52 | 27.7 | 1.4e-07 | 7.1e-06 | 4.8 | 17.11 |
| BSU29680 | 2.24 | 7.11 | 35.2 | 3.0e-09 | 2.1e-07 | 4.7 | 22.21 |
| BSU21770 | 2.23 | 9.83 | 32.7 | 1.1e-08 | 6.5e-07 | 4.7 | 20.55 |
| BSU40180 | 2.21 | 6.74 | 24.3 | 8.2e-07 | 3.4e-05 | 4.6 | 14.84 |
| BSU39880 | 2.21 | 5.30 | 25.2 | 5.1e-07 | 2.2e-05 | 4.6 | 15.49 |
| BSU39910 | 2.18 | 3.62 | 18.5 | 1.7e-05 | 5.2e-04 | 4.5 | 10.92 |
| BSU32530 | 2.14 | 4.74 | 16.2 | 5.7e-05 | 1.6e-03 | 4.4 | 9.29 |
| BSU11880 | 2.13 | 7.22 | 21.8 | 3.1e-06 | 1.2e-04 | 4.4 | 13.03 |
| BSU13560 | 2.11 | 5.43 | 29.7 | 4.9e-08 | 2.7e-06 | 4.3 | 18.48 |
| BSU18560 | 2.10 | 3.76 | 24.8 | 6.3e-07 | 2.6e-05 | 4.3 | 15.22 |
| BSU13000 | 2.09 | 4.61 | 24.2 | 8.8e-07 | 3.6e-05 | 4.3 | 14.76 |
| BSU02860 | 2.09 | 7.31 | 8.9 | 2.8e-03 | 4.8e-02 | 4.2 | 4.38 |
| BSU13550 | 2.05 | 5.32 | 19.4 | 1.0e-05 | 3.5e-04 | 4.1 | 11.48 |
| BSU19060 | 2.03 | 6.00 | 27.9 | 1.3e-07 | 6.7e-06 | 4.1 | 17.19 |
| BSU39360 | 2.03 | 4.25 | 16.1 | 6.0e-05 | 1.6e-03 | 4.1 | 9.25 |
| BSU21150 | 2.02 | 1.99 | 16.1 | 5.9e-05 | 1.6e-03 | 4.0 | 9.26 |
| BSU05880 | 2.01 | 4.29 | 21.9 | 2.8e-06 | 1.1e-04 | 4.0 | 13.11 |
| BSU18110 | 2.00 | 7.66 | 32.5 | 1.2e-08 | 7.1e-07 | 4.0 | 20.43 |
| BSU18410 | 1.98 | 2.63 | 19.4 | 1.1e-05 | 3.5e-04 | 4.0 | 11.47 |
| BSU08820 | 1.98 | 11.10 | 26.8 | 2.3e-07 | 1.1e-05 | 4.0 | 16.51 |
| BSU14320 | 1.98 | 3.98 | 20.6 | 5.6e-06 | 2.0e-04 | 3.9 | 12.27 |
| BSU37560 | 1.97 | 7.67 | 9.0 | 2.7e-03 | 4.7e-02 | 3.9 | 4.41 |
| BSU12560 | 1.96 | 0.38 | 9.6 | 2.0e-03 | 3.6e-02 | 3.9 | 4.78 |
| BSU37680 | 1.96 | 5.68 | 21.9 | 2.9e-06 | 1.1e-04 | 3.9 | 13.11 |
| BSU11190 | 1.95 | 3.90 | 16.3 | 5.4e-05 | 1.6e-03 | 3.9 | 9.33 |
| BSU32240 | 1.95 | 9.96 | 22.9 | 1.8e-06 | 7.1e-05 | 3.9 | 13.78 |
| BSU33710 | 1.94 | 5.98 | 19.3 | 1.1e-05 | 3.7e-04 | 3.8 | 11.39 |
| BSU32260 | 1.94 | 10.51 | 43.7 | 3.7e-11 | 2.8e-09 | 3.8 | 28.41 |
| BSU17150 | 1.94 | 4.31 | 18.8 | 1.5e-05 | 4.6e-04 | 3.8 | 11.07 |
| BSU32250 | 1.93 | 10.56 | 41.6 | 1.1e-10 | 8.2e-09 | 3.8 | 26.86 |
| BSU11870 | 1.91 | 7.37 | 27.4 | 1.7e-07 | 8.1e-06 | 3.8 | 16.91 |
| BSU19050 | 1.90 | 5.71 | 25.8 | 3.7e-07 | 1.6e-05 | 3.7 | 15.92 |
| BSU10390 | 1.89 | 2.12 | 13.1 | 3.0e-04 | 6.7e-03 | 3.7 | 7.22 |
| BSU33770 | 1.86 | 7.78 | 15.7 | 7.5e-05 | 2.0e-03 | 3.6 | 8.98 |
| BSU39370 | 1.82 | 4.25 | 19.1 | 1.2e-05 | 4.0e-04 | 3.5 | 11.29 |
| BSU36650 | 1.80 | 2.14 | 10.5 | 1.2e-03 | 2.4e-02 | 3.5 | 5.39 |
| BSU13190 | 1.80 | 4.47 | 18.8 | 1.4e-05 | 4.6e-04 | 3.5 | 11.09 |
| BSU17180 | 1.79 | 7.45 | 20.5 | 5.9e-06 | 2.1e-04 | 3.5 | 12.21 |
| BSU33730 | 1.78 | 6.15 | 20.9 | 4.8e-06 | 1.8e-04 | 3.4 | 12.47 |
| BSU40021 | 1.78 | 3.85 | 17.9 | 2.3e-05 | 6.9e-04 | 3.4 | 10.51 |
| BSU07840 | 1.76 | 8.83 | 27.6 | 1.5e-07 | 7.7e-06 | 3.4 | 16.99 |
| BSU08470 | 1.76 | 6.95 | 18.9 | 1.4e-05 | 4.5e-04 | 3.4 | 11.12 |
| BSU31010 | 1.74 | 7.90 | 26.5 | 2.6e-07 | 1.2e-05 | 3.3 | 16.32 |
| BSU39390 | 1.74 | 3.13 | 10.5 | 1.2e-03 | 2.3e-02 | 3.3 | 5.45 |
| BSU02490 | 1.74 | 1.79 | 10.9 | 9.9e-04 | 1.9e-02 | 3.3 | 5.68 |
| BSU12430 | 1.74 | 7.86 | 20.2 | 6.9e-06 | 2.4e-04 | 3.3 | 12.01 |
| BSU31020 | 1.73 | 6.22 | 18.3 | 1.8e-05 | 5.6e-04 | 3.3 | 10.81 |
| BSU10300 | 1.71 | 3.25 | 14.4 | 1.5e-04 | 3.7e-03 | 3.3 | 8.09 |
| BSU31000 | 1.71 | 6.22 | 21.4 | 3.6e-06 | 1.4e-04 | 3.3 | 12.82 |
| BSU38950 | 1.70 | 6.84 | 17.4 | 3.0e-05 | 8.9e-04 | 3.2 | 10.14 |
| BSU11890 | 1.69 | 5.31 | 20.5 | 6.1e-06 | 2.2e-04 | 3.2 | 12.18 |
| BSU13580 | 1.69 | 7.32 | 20.2 | 7.1e-06 | 2.5e-04 | 3.2 | 11.99 |
| BSU37740 | 1.68 | 3.15 | 12.1 | 5.0e-04 | 1.1e-02 | 3.2 | 6.56 |
| BSU24090 | 1.68 | 8.53 | 25.9 | 3.7e-07 | 1.6e-05 | 3.2 | 15.92 |
| BSU17170 | 1.68 | 3.24 | 13.9 | 1.9e-04 | 4.5e-03 | 3.2 | 7.79 |
| BSU37750 | 1.67 | 2.06 | 9.0 | 2.7e-03 | 4.7e-02 | 3.2 | 4.40 |
| BSU34660 | 1.66 | 3.97 | 11.1 | 8.7e-04 | 1.7e-02 | 3.2 | 5.85 |
| BSU31480 | 1.66 | 4.06 | 10.3 | 1.3e-03 | 2.5e-02 | 3.2 | 5.30 |
| BSU34190 | 1.65 | 8.32 | 11.8 | 5.9e-04 | 1.2e-02 | 3.1 | 6.35 |
| BSU17160 | 1.64 | 3.82 | 15.5 | 8.2e-05 | 2.1e-03 | 3.1 | 8.87 |
| BSU37470 | 1.63 | 4.57 | 16.2 | 5.6e-05 | 1.6e-03 | 3.1 | 9.30 |
| BSU14330 | 1.62 | 4.05 | 14.1 | 1.7e-04 | 4.0e-03 | 3.1 | 7.95 |
| BSU32520 | 1.60 | 5.15 | 12.8 | 3.4e-04 | 7.4e-03 | 3.0 | 7.08 |
| BSU17190 | 1.59 | 7.58 | 17.5 | 2.9e-05 | 8.5e-04 | 3.0 | 10.20 |
| BSU39380 | 1.58 | 3.72 | 10.0 | 1.6e-03 | 3.0e-02 | 3.0 | 5.06 |
| BSU40022 | 1.58 | 4.55 | 14.3 | 1.6e-04 | 3.8e-03 | 3.0 | 8.05 |
| BSU15350 | 1.58 | 7.50 | 20.3 | 6.6e-06 | 2.3e-04 | 3.0 | 12.08 |
| BSU38550 | 1.57 | 10.15 | 28.3 | 1.0e-07 | 5.5e-06 | 3.0 | 17.48 |
| BSU24080 | 1.55 | 8.83 | 21.5 | 3.6e-06 | 1.4e-04 | 2.9 | 12.84 |
| BSU37460 | 1.54 | 9.42 | 11.8 | 6.1e-04 | 1.3e-02 | 2.9 | 6.32 |
| BSU09990 | 1.50 | 8.54 | 13.4 | 2.5e-04 | 5.8e-03 | 2.8 | 7.43 |
| BSU10230 | 1.49 | 4.84 | 12.3 | 4.4e-04 | 9.4e-03 | 2.8 | 6.73 |
| BSU09670 | 1.47 | 8.52 | 20.0 | 7.9e-06 | 2.7e-04 | 2.8 | 11.86 |
| BSU12460 | 1.46 | 4.40 | 10.9 | 9.5e-04 | 1.9e-02 | 2.8 | 5.72 |
| BSU34670 | 1.46 | 3.18 | 11.1 | 8.5e-04 | 1.7e-02 | 2.7 | 5.88 |
| BSU13620 | 1.44 | 6.75 | 14.8 | 1.2e-04 | 3.0e-03 | 2.7 | 8.40 |
| BSU22640 | 1.43 | 5.33 | 9.7 | 1.8e-03 | 3.4e-02 | 2.7 | 4.87 |
| BSU13610 | 1.42 | 6.70 | 14.9 | 1.1e-04 | 2.9e-03 | 2.7 | 8.45 |
| BSU07580 | 1.42 | 6.23 | 15.0 | 1.1e-04 | 2.7e-03 | 2.7 | 8.53 |
| BSU24070 | 1.41 | 8.47 | 18.7 | 1.6e-05 | 4.8e-04 | 2.7 | 11.01 |
| BSU33430 | 1.40 | 8.09 | 13.3 | 2.7e-04 | 6.1e-03 | 2.6 | 7.36 |
| BSU07600 | 1.40 | 4.67 | 12.8 | 3.5e-04 | 7.7e-03 | 2.6 | 7.03 |
| BSU23820 | 1.36 | 6.72 | 12.7 | 3.6e-04 | 7.9e-03 | 2.6 | 6.99 |
| BSU33830 | 1.35 | 8.67 | 18.2 | 1.9e-05 | 5.8e-04 | 2.5 | 10.75 |
| BSU17210 | 1.34 | 8.08 | 15.3 | 9.0e-05 | 2.3e-03 | 2.5 | 8.75 |
| BSU24060 | 1.34 | 8.93 | 16.1 | 6.1e-05 | 1.6e-03 | 2.5 | 9.25 |
| BSU18440 | 1.33 | 8.24 | 16.2 | 5.8e-05 | 1.6e-03 | 2.5 | 9.28 |
| BSU18000 | 1.33 | 9.96 | 19.1 | 1.3e-05 | 4.1e-04 | 2.5 | 11.26 |
| BSU39920 | 1.32 | 5.14 | 11.8 | 5.9e-04 | 1.2e-02 | 2.5 | 6.35 |
| BSU24050 | 1.32 | 8.78 | 15.7 | 7.2e-05 | 1.9e-03 | 2.5 | 9.02 |
| BSU33440 | 1.31 | 7.95 | 13.0 | 3.2e-04 | 7.0e-03 | 2.5 | 7.16 |
| BSU18450 | 1.31 | 9.61 | 16.6 | 4.7e-05 | 1.4e-03 | 2.5 | 9.53 |
| BSU33810 | 1.30 | 8.17 | 14.4 | 1.5e-04 | 3.7e-03 | 2.5 | 8.09 |
| BSU17200 | 1.30 | 7.49 | 13.0 | 3.1e-04 | 6.9e-03 | 2.5 | 7.17 |
| BSU02410 | 1.29 | 6.12 | 8.9 | 2.8e-03 | 4.8e-02 | 2.4 | 4.38 |
| BSU18570 | 1.25 | 7.87 | 14.7 | 1.2e-04 | 3.1e-03 | 2.4 | 8.34 |
| BSU24040 | 1.25 | 8.75 | 14.3 | 1.5e-04 | 3.7e-03 | 2.4 | 8.07 |
| BSU13590 | 1.25 | 6.86 | 11.3 | 7.8e-04 | 1.6e-02 | 2.4 | 5.98 |
| BSU27200 | 1.24 | 3.86 | 8.9 | 2.8e-03 | 4.8e-02 | 2.4 | 4.38 |
| BSU07590 | 1.24 | 4.29 | 10.0 | 1.6e-03 | 3.0e-02 | 2.4 | 5.08 |
| BSU24030 | 1.23 | 9.27 | 16.4 | 5.2e-05 | 1.5e-03 | 2.4 | 9.37 |
| BSU05860 | 1.23 | 4.20 | 9.5 | 2.1e-03 | 3.8e-02 | 2.3 | 4.72 |
| BSU23680 | 1.23 | 5.44 | 9.6 | 2.0e-03 | 3.6e-02 | 2.3 | 4.79 |
| BSU31450 | 1.21 | 6.21 | 11.2 | 8.0e-04 | 1.6e-02 | 2.3 | 5.96 |
| BSU23070 | 1.20 | 11.55 | 18.6 | 1.6e-05 | 4.9e-04 | 2.3 | 11.00 |
| BSU40100 | 1.20 | 13.13 | 10.9 | 9.7e-04 | 1.9e-02 | 2.3 | 5.70 |
| BSU11840 | 1.18 | 7.04 | 9.3 | 2.3e-03 | 4.1e-02 | 2.3 | 4.61 |
| BSU18010 | 1.17 | 4.59 | 8.9 | 2.8e-03 | 4.8e-02 | 2.3 | 4.38 |
| BSU11860 | 1.17 | 8.11 | 13.3 | 2.6e-04 | 5.9e-03 | 2.2 | 7.40 |
| BSU15600 | 1.16 | 7.54 | 10.8 | 9.9e-04 | 1.9e-02 | 2.2 | 5.68 |
| BSU13600 | 1.16 | 6.68 | 10.0 | 1.5e-03 | 2.9e-02 | 2.2 | 5.09 |
| BSU15590 | 1.16 | 8.37 | 13.4 | 2.5e-04 | 5.7e-03 | 2.2 | 7.45 |
| BSU40090 | 1.15 | 12.17 | 15.2 | 9.8e-05 | 2.5e-03 | 2.2 | 8.63 |
| BSU29690 | 1.13 | 5.29 | 9.4 | 2.2e-03 | 4.0e-02 | 2.2 | 4.66 |
| BSU08780 | 1.12 | 4.97 | 8.9 | 2.9e-03 | 4.9e-02 | 2.2 | 4.34 |
| BSU15630 | 1.12 | 7.10 | 9.1 | 2.5e-03 | 4.4e-02 | 2.2 | 4.50 |
| BSU09450 | 1.12 | 5.14 | 9.4 | 2.1e-03 | 3.9e-02 | 2.2 | 4.69 |
| BSU06170 | 1.10 | 8.39 | 11.8 | 5.9e-04 | 1.2e-02 | 2.1 | 6.35 |
| BSU15570 | 1.10 | 8.16 | 11.3 | 7.9e-04 | 1.6e-02 | 2.1 | 5.96 |
| BSU31400 | 1.09 | 9.12 | 9.4 | 2.2e-03 | 4.0e-02 | 2.1 | 4.64 |
| BSU40340 | 1.08 | 10.85 | 13.9 | 1.9e-04 | 4.5e-03 | 2.1 | 7.80 |
| BSU17220 | 1.06 | 7.35 | 9.0 | 2.8e-03 | 4.8e-02 | 2.1 | 4.39 |
| BSU11850 | 1.06 | 7.65 | 9.1 | 2.6e-03 | 4.5e-02 | 2.1 | 4.48 |
| BSU40420 | -1.15 | 11.41 | 15.9 | 6.6e-05 | 1.8e-03 | -2.2 | 9.15 |
| BSU23560 | -1.16 | 6.94 | 10.0 | 1.6e-03 | 3.0e-02 | -2.2 | 5.08 |
| BSU18120 | -1.22 | 8.45 | 13.6 | 2.2e-04 | 5.2e-03 | -2.3 | 7.59 |
| BSU35920 | -1.27 | 5.14 | 10.3 | 1.3e-03 | 2.5e-02 | -2.4 | 5.31 |
| BSU00090 | -1.32 | 10.74 | 20.8 | 5.0e-06 | 1.8e-04 | -2.5 | 12.40 |
| BSU03380 | -1.40 | 5.52 | 12.1 | 5.1e-04 | 1.1e-02 | -2.6 | 6.54 |
| BSU29990 | -1.43 | 7.78 | 12.0 | 5.3e-04 | 1.1e-02 | -2.7 | 6.49 |
| BSU37660 | -1.46 | 8.98 | 20.4 | 6.2e-06 | 2.2e-04 | -2.8 | 12.16 |
| BSU29470 | -1.66 | 9.60 | 30.3 | 3.6e-08 | 2.1e-06 | -3.2 | 18.89 |
| BSU01630 | -1.70 | 2.08 | 9.6 | 2.0e-03 | 3.6e-02 | -3.2 | 4.78 |
| BSU27090 | -1.70 | 5.20 | 15.5 | 8.3e-05 | 2.2e-03 | -3.3 | 8.85 |
| BSU03370 | -1.71 | 4.29 | 10.2 | 1.4e-03 | 2.7e-02 | -3.3 | 5.22 |
| BSU08440 | -1.76 | 5.02 | 12.4 | 4.2e-04 | 9.0e-03 | -3.4 | 6.80 |
| BSU35930 | -1.86 | 2.81 | 17.4 | 3.0e-05 | 8.7e-04 | -3.6 | 10.16 |
| BSU07150 | -1.88 | 3.75 | 14.2 | 1.6e-04 | 3.9e-03 | -3.7 | 8.01 |
| BSU35960 | -1.94 | 4.58 | 25.2 | 5.2e-07 | 2.2e-05 | -3.8 | 15.45 |
| BSU39020 | -1.98 | 8.64 | 19.8 | 8.6e-06 | 2.9e-04 | -3.9 | 11.75 |
| BSU03670 | -2.00 | 5.72 | 16.3 | 5.5e-05 | 1.6e-03 | -4.0 | 9.33 |
| BSU32130 | -2.00 | 7.62 | 32.7 | 1.1e-08 | 6.5e-07 | -4.0 | 20.55 |
| BSU35940 | -2.07 | 4.78 | 26.4 | 2.8e-07 | 1.3e-05 | -4.2 | 16.27 |
| BSU35950 | -2.16 | 4.06 | 22.2 | 2.5e-06 | 9.9e-05 | -4.5 | 13.31 |
| BSU22260 | -2.98 | 5.63 | 33.9 | 5.8e-09 | 3.7e-07 | -7.9 | 21.36 |
| BSU11780 | -2.98 | -0.23 | 10.2 | 1.4e-03 | 2.7e-02 | -7.9 | 5.21 |
| BSU12860 | -3.85 | 7.43 | 96.0 | 1.1e-22 | 1.6e-20 | -14.4 | 65.77 |
| BSU09680 | -3.91 | 7.24 | 85.8 | 2.0e-20 | 2.8e-18 | -15.0 | 58.34 |
