# Supplementary material for: T-REx: Transcriptome analysis webserver for RNA-seq Expression data
Source: BMC Genomics. 2015 Sep 3;16(1):663. doi: 10.1186/s12864-015-1834-4 (PMC4558784; doi:10.1186/s12864-015-1834-4)
Supplement: Additional file 3: — Figure S3; k-means clustering of differentially expressed genes in the mutants. (ZIP 31925 kb) [file 12864_2015_1834_MOESM3_ESM.zip › Brinsmade.Significant_Changed_Genes.4.Null-WT.txt.html]

| GeneID | logFC | logCPM | LR | pvalue | adj\_pvalue | Fold | minFDR |
| --- | --- | --- | --- | --- | --- | --- | --- |
| BSU32600 | 9.82 | 9.43 | 439.7 | 1.3e-97 | 5.2e-94 | 905.8 | 309.88 |
| BSU32610 | 9.17 | 12.30 | 381.4 | 6.1e-85 | 1.3e-81 | 576.5 | 268.74 |
| BSU32590 | 8.93 | 7.88 | 193.7 | 5.0e-44 | 1.5e-41 | 489.2 | 135.64 |
| BSU32580 | 8.76 | 7.91 | 264.2 | 2.1e-59 | 1.5e-56 | 433.4 | 185.47 |
| BSU09460 | 7.68 | 9.44 | 272.1 | 4.0e-61 | 3.4e-58 | 205.0 | 190.92 |
| BSU02390 | 7.50 | 9.86 | 359.4 | 3.8e-80 | 5.3e-77 | 180.7 | 253.40 |
| BSU32570 | 7.26 | 8.27 | 237.6 | 1.3e-53 | 6.2e-51 | 153.1 | 166.78 |
| BSU03010 | 6.93 | 8.23 | 177.7 | 1.6e-40 | 3.8e-38 | 121.7 | 124.30 |
| BSU32090 | 6.85 | 9.90 | 72.3 | 1.8e-17 | 1.9e-15 | 115.1 | 48.92 |
| BSU32550 | 6.44 | 8.87 | 280.1 | 7.0e-63 | 7.3e-60 | 86.7 | 196.44 |
| BSU31540 | 6.31 | 6.49 | 179.9 | 5.0e-41 | 1.3e-38 | 79.5 | 125.84 |
| BSU19350 | 6.06 | 8.26 | 166.6 | 4.1e-38 | 9.6e-36 | 66.9 | 116.33 |
| BSU10450 | 5.88 | 6.85 | 66.1 | 4.3e-16 | 4.0e-14 | 59.0 | 44.50 |
| BSU10460 | 5.86 | 4.25 | 63.4 | 1.7e-15 | 1.5e-13 | 58.0 | 42.60 |
| BSU31550 | 5.85 | 6.12 | 154.9 | 1.5e-35 | 3.1e-33 | 57.6 | 107.99 |
| BSU31560 | 5.66 | 5.46 | 112.9 | 2.3e-26 | 3.3e-24 | 50.6 | 78.00 |
| BSU39890 | 5.58 | 4.34 | 121.2 | 3.5e-28 | 5.9e-26 | 47.9 | 83.81 |
| BSU13940 | 5.48 | 6.11 | 105.0 | 1.2e-24 | 1.6e-22 | 44.7 | 72.36 |
| BSU12940 | 5.46 | 4.43 | 41.9 | 9.4e-11 | 5.0e-09 | 44.1 | 27.58 |
| BSU39900 | 5.36 | 3.08 | 59.4 | 1.3e-14 | 1.0e-12 | 40.9 | 39.85 |
| BSU12930 | 5.33 | 4.80 | 49.5 | 2.0e-12 | 1.2e-10 | 40.2 | 32.91 |
| BSU12920 | 5.29 | 6.98 | 130.8 | 2.8e-30 | 5.1e-28 | 39.2 | 90.68 |
| BSU11230 | 5.29 | 2.55 | 35.2 | 3.0e-09 | 1.2e-07 | 39.0 | 22.94 |
| BSU31570 | 5.17 | 4.89 | 68.0 | 1.6e-16 | 1.6e-14 | 36.0 | 45.87 |
| BSU28280 | 5.15 | 11.01 | 241.5 | 1.9e-54 | 9.8e-52 | 35.4 | 169.44 |
| BSU12950 | 5.11 | 4.15 | 44.8 | 2.2e-11 | 1.3e-09 | 34.5 | 29.56 |
| BSU28260 | 5.05 | 11.51 | 247.7 | 8.3e-56 | 4.9e-53 | 33.2 | 173.76 |
| BSU28250 | 5.05 | 9.68 | 211.9 | 5.2e-48 | 1.8e-45 | 33.1 | 148.64 |
| BSU28290 | 5.05 | 11.24 | 222.9 | 2.1e-50 | 8.0e-48 | 33.0 | 156.45 |
| BSU28270 | 5.02 | 10.64 | 231.4 | 3.0e-52 | 1.2e-49 | 32.4 | 162.46 |
| BSU11360 | 4.97 | 6.42 | 112.6 | 2.6e-26 | 3.7e-24 | 31.4 | 77.85 |
| BSU28300 | 4.94 | 9.51 | 190.5 | 2.5e-43 | 6.9e-41 | 30.6 | 133.42 |
| BSU10410 | 4.86 | 6.60 | 36.2 | 1.8e-09 | 7.7e-08 | 29.1 | 23.63 |
| BSU28310 | 4.78 | 10.87 | 198.2 | 5.1e-45 | 1.6e-42 | 27.4 | 138.81 |
| BSU32080 | 4.74 | 7.17 | 58.9 | 1.6e-14 | 1.2e-12 | 26.7 | 39.57 |
| BSU11390 | 4.72 | 6.72 | 120.0 | 6.4e-28 | 9.9e-26 | 26.4 | 83.07 |
| BSU11900 | 4.70 | 5.97 | 64.4 | 1.0e-15 | 9.1e-14 | 26.0 | 43.32 |
| BSU11370 | 4.65 | 6.57 | 104.7 | 1.5e-24 | 1.9e-22 | 25.1 | 72.16 |
| BSU11400 | 4.65 | 6.58 | 116.4 | 3.9e-27 | 5.9e-25 | 25.0 | 80.49 |
| BSU32070 | 4.47 | 6.96 | 97.6 | 5.1e-23 | 6.4e-21 | 22.2 | 67.08 |
| BSU11220 | 4.43 | 3.69 | 28.7 | 8.4e-08 | 2.8e-06 | 21.6 | 18.46 |
| BSU12960 | 4.31 | 5.86 | 89.9 | 2.5e-21 | 2.9e-19 | 19.9 | 61.56 |
| BSU11210 | 4.25 | 3.22 | 36.7 | 1.4e-09 | 6.3e-08 | 19.0 | 23.93 |
| BSU21870 | 4.22 | 10.75 | 155.5 | 1.1e-35 | 2.4e-33 | 18.7 | 108.35 |
| BSU03020 | 4.17 | 6.88 | 59.2 | 1.5e-14 | 1.1e-12 | 18.0 | 39.71 |
| BSU03350 | 4.11 | 6.12 | 8.3 | 4.0e-03 | 4.9e-02 | 17.3 | 4.34 |
| BSU10070 | 4.09 | 8.67 | 80.5 | 2.9e-19 | 3.2e-17 | 17.0 | 54.78 |
| BSU15340 | 4.08 | 6.91 | 89.9 | 2.5e-21 | 2.9e-19 | 16.9 | 61.56 |
| BSU39910 | 4.01 | 3.62 | 56.4 | 5.8e-14 | 4.3e-12 | 16.1 | 37.77 |
| BSU07530 | 4.01 | 3.70 | 51.4 | 7.7e-13 | 4.9e-11 | 16.1 | 34.24 |
| BSU39350 | 3.98 | 3.30 | 31.5 | 2.0e-08 | 6.8e-07 | 15.8 | 20.48 |
| BSU39880 | 3.86 | 5.30 | 68.6 | 1.2e-16 | 1.2e-14 | 14.6 | 46.26 |
| BSU37760 | 3.79 | 8.26 | 52.9 | 3.4e-13 | 2.3e-11 | 13.8 | 35.33 |
| BSU38090 | 3.73 | 6.27 | 75.8 | 3.1e-18 | 3.3e-16 | 13.3 | 51.45 |
| BSU26830 | 3.72 | 7.25 | 9.0 | 2.7e-03 | 3.6e-02 | 13.2 | 4.79 |
| BSU12980 | 3.70 | 4.28 | 43.8 | 3.6e-11 | 2.0e-09 | 13.0 | 28.91 |
| BSU03200 | 3.69 | 10.61 | 136.9 | 1.2e-31 | 2.5e-29 | 12.9 | 95.03 |
| BSU39340 | 3.63 | 6.70 | 70.9 | 3.7e-17 | 3.7e-15 | 12.4 | 47.96 |
| BSU11200 | 3.56 | 3.86 | 26.0 | 3.4e-07 | 9.8e-06 | 11.8 | 16.64 |
| BSU03210 | 3.50 | 11.70 | 136.8 | 1.3e-31 | 2.6e-29 | 11.3 | 94.98 |
| BSU19580 | 3.49 | 9.26 | 46.2 | 1.0e-11 | 6.2e-10 | 11.2 | 30.58 |
| BSU21190 | 3.45 | 0.62 | 28.3 | 1.0e-07 | 3.4e-06 | 10.9 | 18.19 |
| BSU19579 | 3.40 | 7.87 | 60.0 | 9.3e-15 | 7.3e-13 | 10.5 | 40.31 |
| BSU38960 | 3.40 | 8.25 | 78.4 | 8.4e-19 | 9.0e-17 | 10.5 | 53.30 |
| BSU32530 | 3.35 | 4.74 | 36.5 | 1.5e-09 | 6.8e-08 | 10.2 | 23.81 |
| BSU03790 | 3.34 | 8.42 | 93.4 | 4.3e-22 | 5.3e-20 | 10.1 | 64.02 |
| BSU33710 | 3.34 | 5.98 | 51.5 | 7.0e-13 | 4.6e-11 | 10.1 | 34.35 |
| BSU12970 | 3.34 | 4.04 | 54.8 | 1.3e-13 | 9.5e-12 | 10.1 | 36.61 |
| BSU33700 | 3.30 | 5.36 | 20.8 | 5.2e-06 | 1.2e-04 | 9.9 | 12.99 |
| BSU13000 | 3.28 | 4.61 | 54.9 | 1.3e-13 | 9.1e-12 | 9.7 | 36.68 |
| BSU02170 | 3.26 | 8.46 | 25.8 | 3.7e-07 | 1.1e-05 | 9.6 | 16.49 |
| BSU33730 | 3.24 | 6.15 | 62.5 | 2.7e-15 | 2.3e-13 | 9.5 | 41.99 |
| BSU12990 | 3.24 | 4.52 | 53.7 | 2.3e-13 | 1.6e-11 | 9.5 | 35.86 |
| BSU11240 | 3.23 | 4.08 | 25.3 | 4.9e-07 | 1.4e-05 | 9.4 | 16.14 |
| BSU33720 | 3.14 | 4.95 | 21.7 | 3.3e-06 | 8.2e-05 | 8.8 | 13.58 |
| BSU21180 | 3.12 | -0.69 | 8.3 | 3.9e-03 | 4.8e-02 | 8.7 | 4.37 |
| BSU17140 | 3.08 | 4.08 | 47.7 | 5.1e-12 | 3.1e-10 | 8.5 | 31.58 |
| BSU12400 | 3.07 | 3.87 | 27.4 | 1.7e-07 | 5.1e-06 | 8.4 | 17.57 |
| BSU38930 | 3.06 | 1.98 | 18.2 | 2.0e-05 | 4.4e-04 | 8.3 | 11.15 |
| BSU29350 | 3.05 | -0.44 | 8.6 | 3.5e-03 | 4.4e-02 | 8.3 | 4.51 |
| BSU17820 | 3.05 | 1.69 | 25.2 | 5.1e-07 | 1.4e-05 | 8.3 | 16.11 |
| BSU02160 | 3.05 | 8.09 | 52.8 | 3.6e-13 | 2.4e-11 | 8.3 | 35.28 |
| BSU17130 | 3.04 | 1.88 | 32.4 | 1.3e-08 | 4.6e-07 | 8.2 | 21.06 |
| BSU38770 | 3.01 | 5.03 | 35.6 | 2.4e-09 | 1.0e-07 | 8.0 | 23.21 |
| BSU11010 | 3.00 | 5.16 | 35.4 | 2.6e-09 | 1.1e-07 | 8.0 | 23.12 |
| BSU06310 | 2.98 | 6.64 | 39.4 | 3.5e-10 | 1.8e-08 | 7.9 | 25.73 |
| BSU13180 | 2.95 | 7.15 | 62.2 | 3.1e-15 | 2.5e-13 | 7.7 | 41.84 |
| BSU02250 | 2.94 | 5.48 | 54.3 | 1.7e-13 | 1.2e-11 | 7.7 | 36.27 |
| BSU37770 | 2.94 | 8.97 | 80.3 | 3.2e-19 | 3.5e-17 | 7.7 | 54.66 |
| BSU02850 | 2.92 | 11.20 | 9.1 | 2.6e-03 | 3.4e-02 | 7.6 | 4.87 |
| BSU02240 | 2.92 | 7.33 | 62.3 | 2.9e-15 | 2.4e-13 | 7.5 | 41.90 |
| BSU11190 | 2.88 | 3.90 | 33.8 | 6.0e-09 | 2.3e-07 | 7.4 | 22.06 |
| BSU27100 | 2.88 | 6.38 | 44.5 | 2.6e-11 | 1.5e-09 | 7.3 | 29.36 |
| BSU21410 | 2.87 | -0.90 | 8.6 | 3.4e-03 | 4.3e-02 | 7.3 | 4.53 |
| BSU17150 | 2.84 | 4.31 | 38.4 | 5.7e-10 | 2.7e-08 | 7.2 | 25.13 |
| BSU39920 | 2.78 | 5.14 | 48.1 | 4.0e-12 | 2.5e-10 | 6.9 | 31.90 |
| BSU18550 | 2.77 | 4.33 | 43.0 | 5.4e-11 | 2.9e-09 | 6.8 | 28.37 |
| BSU18540 | 2.75 | 4.23 | 36.1 | 1.8e-09 | 8.0e-08 | 6.7 | 23.58 |
| BSU11410 | 2.75 | 3.75 | 37.1 | 1.1e-09 | 5.0e-08 | 6.7 | 24.26 |
| BSU03220 | 2.74 | 10.80 | 43.1 | 5.2e-11 | 2.8e-09 | 6.7 | 28.41 |
| BSU33770 | 2.70 | 7.78 | 30.9 | 2.8e-08 | 9.4e-07 | 6.5 | 20.02 |
| BSU32520 | 2.64 | 5.15 | 32.8 | 1.0e-08 | 3.8e-07 | 6.2 | 21.34 |
| BSU39930 | 2.64 | 3.83 | 38.8 | 4.8e-10 | 2.4e-08 | 6.2 | 25.34 |
| BSU17170 | 2.63 | 3.24 | 33.2 | 8.3e-09 | 3.1e-07 | 6.2 | 21.62 |
| BSU21770 | 2.62 | 9.83 | 43.7 | 3.7e-11 | 2.1e-09 | 6.1 | 28.86 |
| BSU17160 | 2.61 | 3.82 | 37.8 | 7.9e-10 | 3.7e-08 | 6.1 | 24.69 |
| BSU19040 | 2.58 | 4.95 | 37.5 | 9.0e-10 | 4.2e-08 | 6.0 | 24.51 |
| BSU29680 | 2.56 | 7.11 | 45.1 | 1.9e-11 | 1.1e-09 | 5.9 | 29.78 |
| BSU17180 | 2.55 | 7.45 | 39.3 | 3.6e-10 | 1.8e-08 | 5.8 | 25.73 |
| BSU13190 | 2.53 | 4.47 | 35.7 | 2.3e-09 | 9.7e-08 | 5.8 | 23.29 |
| BSU33760 | 2.52 | 6.57 | 16.6 | 4.7e-05 | 1.0e-03 | 5.8 | 9.97 |
| BSU21150 | 2.52 | 1.99 | 25.3 | 4.9e-07 | 1.4e-05 | 5.7 | 16.14 |
| BSU39360 | 2.52 | 4.25 | 24.1 | 9.1e-07 | 2.4e-05 | 5.7 | 15.33 |
| BSU10390 | 2.51 | 2.12 | 22.9 | 1.7e-06 | 4.4e-05 | 5.7 | 14.48 |
| BSU39870 | 2.50 | 3.10 | 24.2 | 8.7e-07 | 2.4e-05 | 5.7 | 15.37 |
| BSU18560 | 2.50 | 3.76 | 34.6 | 4.0e-09 | 1.6e-07 | 5.6 | 22.58 |
| BSU36660 | 2.48 | 1.81 | 20.9 | 4.9e-06 | 1.2e-04 | 5.6 | 13.07 |
| BSU13560 | 2.45 | 5.43 | 39.2 | 3.8e-10 | 1.9e-08 | 5.5 | 25.65 |
| BSU40180 | 2.45 | 6.74 | 29.4 | 6.0e-08 | 2.0e-06 | 5.5 | 18.95 |
| BSU05870 | 2.43 | 4.27 | 33.9 | 5.7e-09 | 2.2e-07 | 5.4 | 22.10 |
| BSU37480 | 2.41 | 4.66 | 37.3 | 9.9e-10 | 4.5e-08 | 5.3 | 24.39 |
| BSU11890 | 2.40 | 5.31 | 39.8 | 2.8e-10 | 1.5e-08 | 5.3 | 26.02 |
| BSU18110 | 2.40 | 7.66 | 45.5 | 1.5e-11 | 9.1e-10 | 5.3 | 30.03 |
| BSU32250 | 2.39 | 10.56 | 62.1 | 3.2e-15 | 2.6e-13 | 5.3 | 41.83 |
| BSU21090 | 2.39 | 0.51 | 10.6 | 1.1e-03 | 1.7e-02 | 5.2 | 5.89 |
| BSU32240 | 2.39 | 9.96 | 33.3 | 7.7e-09 | 2.9e-07 | 5.2 | 21.71 |
| BSU32260 | 2.38 | 10.51 | 63.8 | 1.4e-15 | 1.2e-13 | 5.2 | 42.88 |
| BSU05880 | 2.35 | 4.29 | 29.5 | 5.5e-08 | 1.8e-06 | 5.1 | 19.05 |
| BSU13550 | 2.34 | 5.32 | 24.8 | 6.5e-07 | 1.8e-05 | 5.1 | 15.76 |
| BSU14320 | 2.33 | 3.98 | 28.1 | 1.1e-07 | 3.6e-06 | 5.0 | 18.09 |
| BSU11880 | 2.33 | 7.22 | 25.5 | 4.3e-07 | 1.3e-05 | 5.0 | 16.29 |
| BSU02400 | 2.31 | 7.79 | 24.6 | 7.0e-07 | 1.9e-05 | 4.9 | 15.66 |
| BSU17190 | 2.29 | 7.58 | 34.8 | 3.7e-09 | 1.5e-07 | 4.9 | 22.70 |
| BSU36650 | 2.29 | 2.14 | 16.7 | 4.3e-05 | 9.3e-04 | 4.9 | 10.07 |
| BSU10570 | 2.26 | 4.72 | 11.7 | 6.2e-04 | 1.0e-02 | 4.8 | 6.60 |
| BSU40021 | 2.25 | 3.85 | 28.2 | 1.1e-07 | 3.5e-06 | 4.8 | 18.14 |
| BSU39370 | 2.21 | 4.25 | 27.8 | 1.3e-07 | 4.1e-06 | 4.6 | 17.88 |
| BSU18410 | 2.20 | 2.63 | 23.7 | 1.1e-06 | 2.9e-05 | 4.6 | 15.06 |
| BSU11870 | 2.17 | 7.37 | 34.8 | 3.6e-09 | 1.4e-07 | 4.5 | 22.72 |
| BSU19060 | 2.17 | 6.00 | 31.6 | 1.9e-08 | 6.8e-07 | 4.5 | 20.49 |
| BSU38450 | 2.16 | 6.08 | 29.7 | 5.1e-08 | 1.7e-06 | 4.5 | 19.16 |
| BSU32510 | 2.12 | 0.68 | 9.6 | 1.9e-03 | 2.7e-02 | 4.3 | 5.20 |
| BSU12440 | 2.10 | 1.94 | 8.7 | 3.2e-03 | 4.1e-02 | 4.3 | 4.60 |
| BSU19050 | 2.09 | 5.71 | 30.9 | 2.7e-08 | 9.4e-07 | 4.3 | 20.02 |
| BSU21229 | 2.07 | 0.55 | 11.9 | 5.5e-04 | 9.3e-03 | 4.2 | 6.74 |
| BSU34660 | 2.07 | 3.97 | 16.8 | 4.1e-05 | 8.9e-04 | 4.2 | 10.14 |
| BSU24090 | 2.06 | 8.53 | 37.9 | 7.3e-10 | 3.5e-08 | 4.2 | 24.78 |
| BSU15350 | 2.06 | 7.50 | 33.4 | 7.6e-09 | 2.9e-07 | 4.2 | 21.72 |
| BSU31480 | 2.03 | 4.06 | 15.2 | 9.6e-05 | 1.9e-03 | 4.1 | 9.03 |
| BSU12560 | 2.02 | 0.38 | 10.1 | 1.5e-03 | 2.2e-02 | 4.1 | 5.54 |
| BSU08470 | 2.01 | 6.95 | 24.1 | 9.0e-07 | 2.4e-05 | 4.0 | 15.34 |
| BSU39390 | 2.00 | 3.13 | 13.7 | 2.1e-04 | 3.9e-03 | 4.0 | 7.99 |
| BSU31020 | 1.99 | 6.22 | 24.1 | 9.1e-07 | 2.4e-05 | 4.0 | 15.33 |
| BSU18440 | 1.99 | 8.24 | 34.7 | 3.8e-09 | 1.5e-07 | 4.0 | 22.64 |
| BSU08820 | 1.97 | 11.10 | 26.5 | 2.6e-07 | 7.9e-06 | 3.9 | 16.95 |
| BSU34190 | 1.96 | 8.32 | 16.4 | 5.0e-05 | 1.1e-03 | 3.9 | 9.90 |
| BSU22670 | 1.96 | 3.61 | 8.9 | 2.9e-03 | 3.8e-02 | 3.9 | 4.72 |
| BSU12430 | 1.96 | 7.86 | 25.4 | 4.7e-07 | 1.3e-05 | 3.9 | 16.19 |
| BSU18450 | 1.95 | 9.61 | 35.5 | 2.5e-09 | 1.1e-07 | 3.9 | 23.17 |
| BSU11250 | 1.95 | 3.04 | 10.0 | 1.6e-03 | 2.3e-02 | 3.9 | 5.44 |
| BSU31010 | 1.94 | 7.90 | 32.5 | 1.2e-08 | 4.2e-07 | 3.8 | 21.17 |
| BSU37680 | 1.93 | 5.68 | 21.3 | 3.9e-06 | 9.8e-05 | 3.8 | 13.32 |
| BSU40022 | 1.93 | 4.55 | 20.9 | 4.8e-06 | 1.1e-04 | 3.8 | 13.09 |
| BSU07840 | 1.93 | 8.83 | 32.7 | 1.1e-08 | 3.9e-07 | 3.8 | 21.28 |
| BSU39380 | 1.92 | 3.72 | 14.4 | 1.5e-04 | 2.8e-03 | 3.8 | 8.46 |
| BSU32950 | 1.91 | 1.89 | 15.8 | 7.0e-05 | 1.4e-03 | 3.8 | 9.44 |
| BSU37460 | 1.90 | 9.42 | 17.5 | 2.9e-05 | 6.4e-04 | 3.7 | 10.61 |
| BSU29330 | 1.90 | 0.80 | 10.3 | 1.3e-03 | 1.9e-02 | 3.7 | 5.69 |
| BSU24080 | 1.88 | 8.83 | 31.1 | 2.4e-08 | 8.4e-07 | 3.7 | 20.18 |
| BSU17200 | 1.87 | 7.49 | 26.1 | 3.2e-07 | 9.5e-06 | 3.7 | 16.68 |
| BSU38950 | 1.87 | 6.84 | 20.8 | 5.1e-06 | 1.2e-04 | 3.7 | 13.02 |
| BSU14330 | 1.85 | 4.05 | 18.3 | 1.9e-05 | 4.2e-04 | 3.6 | 11.22 |
| BSU03530 | 1.84 | 6.68 | 8.5 | 3.6e-03 | 4.5e-02 | 3.6 | 4.47 |
| BSU31000 | 1.84 | 6.22 | 24.7 | 6.7e-07 | 1.8e-05 | 3.6 | 15.72 |
| BSU13580 | 1.84 | 7.32 | 23.6 | 1.2e-06 | 3.1e-05 | 3.6 | 14.96 |
| BSU18000 | 1.80 | 9.96 | 34.0 | 5.5e-09 | 2.1e-07 | 3.5 | 22.16 |
| BSU09530 | 1.79 | 3.74 | 10.6 | 1.1e-03 | 1.7e-02 | 3.5 | 5.88 |
| BSU12520 | 1.79 | 1.16 | 11.2 | 8.3e-04 | 1.3e-02 | 3.5 | 6.24 |
| BSU17210 | 1.78 | 8.08 | 26.5 | 2.6e-07 | 7.9e-06 | 3.4 | 16.95 |
| BSU29310 | 1.78 | 1.40 | 12.5 | 4.0e-04 | 7.2e-03 | 3.4 | 7.12 |
| BSU21220 | 1.78 | 1.27 | 9.9 | 1.7e-03 | 2.4e-02 | 3.4 | 5.39 |
| BSU09990 | 1.77 | 8.54 | 18.5 | 1.7e-05 | 3.8e-04 | 3.4 | 11.35 |
| BSU04800 | 1.75 | 6.48 | 9.0 | 2.7e-03 | 3.6e-02 | 3.4 | 4.79 |
| BSU12460 | 1.75 | 4.40 | 15.3 | 9.0e-05 | 1.8e-03 | 3.4 | 9.12 |
| BSU12800 | 1.73 | 3.18 | 14.5 | 1.4e-04 | 2.6e-03 | 3.3 | 8.56 |
| BSU29450 | 1.71 | 4.35 | 13.2 | 2.9e-04 | 5.3e-03 | 3.3 | 7.56 |
| BSU37750 | 1.70 | 2.06 | 9.4 | 2.2e-03 | 3.0e-02 | 3.3 | 5.05 |
| BSU33430 | 1.70 | 8.09 | 19.1 | 1.2e-05 | 2.9e-04 | 3.2 | 11.76 |
| BSU24070 | 1.69 | 8.47 | 26.3 | 2.9e-07 | 8.7e-06 | 3.2 | 16.81 |
| BSU32560 | 1.68 | 6.76 | 15.7 | 7.4e-05 | 1.5e-03 | 3.2 | 9.38 |
| BSU38550 | 1.67 | 10.15 | 31.6 | 1.9e-08 | 6.8e-07 | 3.2 | 20.49 |
| BSU15590 | 1.66 | 8.37 | 26.9 | 2.2e-07 | 6.7e-06 | 3.2 | 17.19 |
| BSU33800 | 1.64 | 7.42 | 8.9 | 2.8e-03 | 3.7e-02 | 3.1 | 4.75 |
| BSU13570 | 1.64 | 6.62 | 9.2 | 2.4e-03 | 3.3e-02 | 3.1 | 4.94 |
| BSU15600 | 1.64 | 7.54 | 21.1 | 4.4e-06 | 1.1e-04 | 3.1 | 13.19 |
| BSU31450 | 1.64 | 6.21 | 20.2 | 6.9e-06 | 1.6e-04 | 3.1 | 12.59 |
| BSU37470 | 1.63 | 4.57 | 16.4 | 5.3e-05 | 1.1e-03 | 3.1 | 9.83 |
| BSU28340 | 1.63 | 1.87 | 11.0 | 9.2e-04 | 1.4e-02 | 3.1 | 6.12 |
| BSU13620 | 1.63 | 6.75 | 18.7 | 1.5e-05 | 3.4e-04 | 3.1 | 11.51 |
| BSU10300 | 1.62 | 3.25 | 12.9 | 3.3e-04 | 6.0e-03 | 3.1 | 7.38 |
| BSU15610 | 1.62 | 8.15 | 17.4 | 3.1e-05 | 6.7e-04 | 3.1 | 10.55 |
| BSU27200 | 1.62 | 3.86 | 14.9 | 1.2e-04 | 2.3e-03 | 3.1 | 8.77 |
| BSU07580 | 1.61 | 6.23 | 19.2 | 1.2e-05 | 2.8e-04 | 3.1 | 11.83 |
| BSU38470 | 1.60 | 2.83 | 10.1 | 1.5e-03 | 2.1e-02 | 3.0 | 5.55 |
| BSU07590 | 1.59 | 4.29 | 16.3 | 5.4e-05 | 1.1e-03 | 3.0 | 9.80 |
| BSU12619 | 1.59 | 3.43 | 11.1 | 8.8e-04 | 1.4e-02 | 3.0 | 6.18 |
| BSU13610 | 1.58 | 6.70 | 18.4 | 1.8e-05 | 4.0e-04 | 3.0 | 11.27 |
| BSU15580 | 1.58 | 7.82 | 13.0 | 3.1e-04 | 5.6e-03 | 3.0 | 7.47 |
| BSU12770 | 1.58 | 1.60 | 9.3 | 2.3e-03 | 3.1e-02 | 3.0 | 4.99 |
| BSU24060 | 1.58 | 8.93 | 22.1 | 2.6e-06 | 6.6e-05 | 3.0 | 13.88 |
| BSU33440 | 1.57 | 7.95 | 18.4 | 1.8e-05 | 4.0e-04 | 3.0 | 11.27 |
| BSU18570 | 1.57 | 7.87 | 22.9 | 1.7e-06 | 4.4e-05 | 3.0 | 14.48 |
| BSU38460 | 1.55 | 4.16 | 15.1 | 1.0e-04 | 2.0e-03 | 2.9 | 8.97 |
| BSU15570 | 1.55 | 8.16 | 21.8 | 3.0e-06 | 7.5e-05 | 2.9 | 13.71 |
| BSU12600 | 1.55 | 4.73 | 14.7 | 1.2e-04 | 2.4e-03 | 2.9 | 8.68 |
| BSU29440 | 1.55 | 4.81 | 12.7 | 3.7e-04 | 6.7e-03 | 2.9 | 7.23 |
| BSU24050 | 1.53 | 8.78 | 21.0 | 4.5e-06 | 1.1e-04 | 2.9 | 13.16 |
| BSU09670 | 1.52 | 8.52 | 21.1 | 4.4e-06 | 1.1e-04 | 2.9 | 13.19 |
| BSU22640 | 1.51 | 5.33 | 10.7 | 1.1e-03 | 1.6e-02 | 2.8 | 5.94 |
| BSU24030 | 1.50 | 9.27 | 23.9 | 9.9e-07 | 2.6e-05 | 2.8 | 15.21 |
| BSU12660 | 1.50 | 4.27 | 12.7 | 3.7e-04 | 6.7e-03 | 2.8 | 7.22 |
| BSU15630 | 1.49 | 7.10 | 15.9 | 6.6e-05 | 1.4e-03 | 2.8 | 9.52 |
| BSU12570 | 1.49 | 4.11 | 9.4 | 2.1e-03 | 3.0e-02 | 2.8 | 5.08 |
| BSU33810 | 1.49 | 8.17 | 18.7 | 1.5e-05 | 3.4e-04 | 2.8 | 11.51 |
| BSU33830 | 1.49 | 8.67 | 22.1 | 2.6e-06 | 6.7e-05 | 2.8 | 13.87 |
| BSU05860 | 1.49 | 4.20 | 13.8 | 2.1e-04 | 3.9e-03 | 2.8 | 8.02 |
| BSU18010 | 1.48 | 4.59 | 14.0 | 1.9e-04 | 3.5e-03 | 2.8 | 8.15 |
| BSU29700 | 1.47 | 4.91 | 15.5 | 8.2e-05 | 1.7e-03 | 2.8 | 9.23 |
| BSU12590 | 1.47 | 4.68 | 12.6 | 3.8e-04 | 6.8e-03 | 2.8 | 7.21 |
| BSU07600 | 1.47 | 4.67 | 14.0 | 1.8e-04 | 3.5e-03 | 2.8 | 8.17 |
| BSU27220 | 1.47 | 5.53 | 13.8 | 2.0e-04 | 3.8e-03 | 2.8 | 8.03 |
| BSU17220 | 1.46 | 7.35 | 16.7 | 4.4e-05 | 9.4e-04 | 2.8 | 10.05 |
| BSU12649 | 1.46 | 3.20 | 8.6 | 3.3e-03 | 4.2e-02 | 2.8 | 4.56 |
| BSU12630 | 1.46 | 3.86 | 11.7 | 6.3e-04 | 1.0e-02 | 2.8 | 6.58 |
| BSU09450 | 1.46 | 5.14 | 15.7 | 7.3e-05 | 1.5e-03 | 2.7 | 9.39 |
| BSU01910 | 1.45 | 2.75 | 8.7 | 3.2e-03 | 4.2e-02 | 2.7 | 4.58 |
| BSU23070 | 1.45 | 11.55 | 26.8 | 2.2e-07 | 6.7e-06 | 2.7 | 17.18 |
| BSU34670 | 1.45 | 3.18 | 10.9 | 9.4e-04 | 1.5e-02 | 2.7 | 6.10 |
| BSU12580 | 1.44 | 4.44 | 13.0 | 3.0e-04 | 5.6e-03 | 2.7 | 7.48 |
| BSU24040 | 1.44 | 8.75 | 19.0 | 1.3e-05 | 3.0e-04 | 2.7 | 11.72 |
| BSU12780 | 1.44 | 3.94 | 10.3 | 1.3e-03 | 1.9e-02 | 2.7 | 5.69 |
| BSU12810 | 1.43 | 5.10 | 12.8 | 3.4e-04 | 6.2e-03 | 2.7 | 7.33 |
| BSU08780 | 1.43 | 4.97 | 14.2 | 1.7e-04 | 3.2e-03 | 2.7 | 8.28 |
| BSU12750 | 1.41 | 4.96 | 9.3 | 2.3e-03 | 3.1e-02 | 2.7 | 5.02 |
| BSU13600 | 1.41 | 6.68 | 14.6 | 1.3e-04 | 2.6e-03 | 2.7 | 8.60 |
| BSU22660 | 1.40 | 4.01 | 11.5 | 7.1e-04 | 1.2e-02 | 2.6 | 6.43 |
| BSU12640 | 1.40 | 3.53 | 11.0 | 9.1e-04 | 1.4e-02 | 2.6 | 6.13 |
| BSU15100 | 1.40 | 4.55 | 12.4 | 4.3e-04 | 7.6e-03 | 2.6 | 7.04 |
| BSU26770 | 1.40 | 4.05 | 11.4 | 7.5e-04 | 1.2e-02 | 2.6 | 6.35 |
| BSU31420 | 1.40 | 1.53 | 8.7 | 3.1e-03 | 4.1e-02 | 2.6 | 4.62 |
| BSU12760 | 1.39 | 2.39 | 8.8 | 3.1e-03 | 4.0e-02 | 2.6 | 4.63 |
| BSU15620 | 1.38 | 8.17 | 18.7 | 1.5e-05 | 3.4e-04 | 2.6 | 11.51 |
| BSU32980 | 1.38 | 4.71 | 13.1 | 2.9e-04 | 5.3e-03 | 2.6 | 7.55 |
| BSU12650 | 1.37 | 5.87 | 12.2 | 4.7e-04 | 8.1e-03 | 2.6 | 6.94 |
| BSU12610 | 1.35 | 4.92 | 11.3 | 7.9e-04 | 1.3e-02 | 2.6 | 6.30 |
| BSU03230 | 1.33 | 7.03 | 10.4 | 1.2e-03 | 1.8e-02 | 2.5 | 5.76 |
| BSU11860 | 1.33 | 8.11 | 17.1 | 3.5e-05 | 7.5e-04 | 2.5 | 10.38 |
| BSU11840 | 1.32 | 7.04 | 11.5 | 6.9e-04 | 1.1e-02 | 2.5 | 6.46 |
| BSU23820 | 1.32 | 6.72 | 11.9 | 5.5e-04 | 9.3e-03 | 2.5 | 6.75 |
| BSU09440 | 1.32 | 5.43 | 12.9 | 3.3e-04 | 5.9e-03 | 2.5 | 7.40 |
| BSU27230 | 1.31 | 3.33 | 9.4 | 2.2e-03 | 3.0e-02 | 2.5 | 5.05 |
| BSU13590 | 1.31 | 6.86 | 12.4 | 4.2e-04 | 7.5e-03 | 2.5 | 7.06 |
| BSU03030 | 1.31 | 4.55 | 11.8 | 5.8e-04 | 9.8e-03 | 2.5 | 6.68 |
| BSU36640 | 1.31 | 4.87 | 9.5 | 2.0e-03 | 2.8e-02 | 2.5 | 5.16 |
| BSU21750 | 1.26 | 5.94 | 9.4 | 2.2e-03 | 3.0e-02 | 2.4 | 5.07 |
| BSU29690 | 1.24 | 5.29 | 11.3 | 7.7e-04 | 1.2e-02 | 2.4 | 6.32 |
| BSU12700 | 1.24 | 3.15 | 8.4 | 3.7e-03 | 4.7e-02 | 2.4 | 4.42 |
| BSU06170 | 1.23 | 8.39 | 14.7 | 1.3e-04 | 2.5e-03 | 2.4 | 8.65 |
| BSU23670 | 1.22 | 7.22 | 10.8 | 1.0e-03 | 1.5e-02 | 2.3 | 6.01 |
| BSU11850 | 1.22 | 7.65 | 12.0 | 5.3e-04 | 9.1e-03 | 2.3 | 6.78 |
| BSU13850 | 1.18 | 9.94 | 10.5 | 1.2e-03 | 1.8e-02 | 2.3 | 5.79 |
| BSU40320 | 1.16 | 10.25 | 9.9 | 1.7e-03 | 2.4e-02 | 2.2 | 5.38 |
| BSU40340 | 1.14 | 10.85 | 15.5 | 8.2e-05 | 1.7e-03 | 2.2 | 9.24 |
| BSU35310 | 1.13 | 8.42 | 11.7 | 6.3e-04 | 1.0e-02 | 2.2 | 6.59 |
| BSU28240 | 1.11 | 7.96 | 10.3 | 1.3e-03 | 1.9e-02 | 2.2 | 5.69 |
| BSU17120 | 1.11 | 5.17 | 8.7 | 3.2e-03 | 4.2e-02 | 2.2 | 4.58 |
| BSU31400 | 1.10 | 9.12 | 9.6 | 1.9e-03 | 2.7e-02 | 2.1 | 5.22 |
| BSU40100 | 1.07 | 13.13 | 8.8 | 3.0e-03 | 4.0e-02 | 2.1 | 4.66 |
| BSU09590 | 1.06 | 8.64 | 12.0 | 5.4e-04 | 9.1e-03 | 2.1 | 6.78 |
| BSU02780 | 1.02 | 9.30 | 11.1 | 8.4e-04 | 1.3e-02 | 2.0 | 6.23 |
| BSU40090 | 1.02 | 12.17 | 12.0 | 5.2e-04 | 9.0e-03 | 2.0 | 6.80 |
| BSU29140 | 1.01 | 8.65 | 10.4 | 1.2e-03 | 1.8e-02 | 2.0 | 5.77 |
| BSU15550 | -1.01 | 8.93 | 10.3 | 1.3e-03 | 1.9e-02 | -2.0 | 5.69 |
| BSU06420 | -1.03 | 9.07 | 9.3 | 2.3e-03 | 3.1e-02 | -2.0 | 4.99 |
| BSU15530 | -1.03 | 8.76 | 10.5 | 1.2e-03 | 1.8e-02 | -2.0 | 5.78 |
| BSU15520 | -1.03 | 10.63 | 12.5 | 4.2e-04 | 7.4e-03 | -2.0 | 7.08 |
| BSU15510 | -1.05 | 9.08 | 11.2 | 8.3e-04 | 1.3e-02 | -2.1 | 6.24 |
| BSU15500 | -1.05 | 8.95 | 11.5 | 7.0e-04 | 1.1e-02 | -2.1 | 6.46 |
| BSU15490 | -1.05 | 8.39 | 11.2 | 8.4e-04 | 1.3e-02 | -2.1 | 6.24 |
| BSU14700 | -1.05 | 5.70 | 8.3 | 4.1e-03 | 5.0e-02 | -2.1 | 4.32 |
| BSU15540 | -1.07 | 8.96 | 8.3 | 4.0e-03 | 5.0e-02 | -2.1 | 4.32 |
| BSU15560 | -1.10 | 8.78 | 11.2 | 8.2e-04 | 1.3e-02 | -2.1 | 6.24 |
| BSU35920 | -1.16 | 5.14 | 8.7 | 3.1e-03 | 4.1e-02 | -2.2 | 4.61 |
| BSU23560 | -1.17 | 6.94 | 10.0 | 1.5e-03 | 2.2e-02 | -2.2 | 5.50 |
| BSU38810 | -1.25 | 4.48 | 9.4 | 2.2e-03 | 3.0e-02 | -2.4 | 5.07 |
| BSU38270 | -1.31 | 5.71 | 11.0 | 9.3e-04 | 1.4e-02 | -2.5 | 6.11 |
| BSU05840 | -1.40 | 2.36 | 8.9 | 2.8e-03 | 3.7e-02 | -2.6 | 4.76 |
| BSU37660 | -1.49 | 8.98 | 21.1 | 4.5e-06 | 1.1e-04 | -2.8 | 13.17 |
| BSU40420 | -1.50 | 11.41 | 26.6 | 2.5e-07 | 7.5e-06 | -2.8 | 17.03 |
| BSU18120 | -1.53 | 8.45 | 21.1 | 4.3e-06 | 1.1e-04 | -2.9 | 13.19 |
| BSU08240 | -1.58 | 3.09 | 9.8 | 1.8e-03 | 2.5e-02 | -3.0 | 5.33 |
| BSU33320 | -1.61 | 3.05 | 10.5 | 1.2e-03 | 1.8e-02 | -3.1 | 5.78 |
| BSU27090 | -1.65 | 5.20 | 14.6 | 1.4e-04 | 2.6e-03 | -3.1 | 8.58 |
| BSU01630 | -1.71 | 2.08 | 9.7 | 1.8e-03 | 2.6e-02 | -3.3 | 5.28 |
| BSU35930 | -1.72 | 2.81 | 15.1 | 1.0e-04 | 2.1e-03 | -3.3 | 8.92 |
| BSU31970 | -1.80 | 1.54 | 10.9 | 9.5e-04 | 1.5e-02 | -3.5 | 6.09 |
| BSU00090 | -1.82 | 10.74 | 38.7 | 5.0e-10 | 2.4e-08 | -3.5 | 25.28 |
| BSU29990 | -1.83 | 7.78 | 19.2 | 1.1e-05 | 2.7e-04 | -3.6 | 11.86 |
| BSU08450 | -1.83 | 3.88 | 8.3 | 4.0e-03 | 5.0e-02 | -3.6 | 4.32 |
| BSU29470 | -1.84 | 9.60 | 36.7 | 1.4e-09 | 6.2e-08 | -3.6 | 23.93 |
| BSU01600 | -1.89 | 0.82 | 9.0 | 2.8e-03 | 3.7e-02 | -3.7 | 4.77 |
| BSU33310 | -1.89 | 2.56 | 8.4 | 3.7e-03 | 4.7e-02 | -3.7 | 4.41 |
| BSU39610 | -1.90 | 1.78 | 12.0 | 5.4e-04 | 9.1e-03 | -3.7 | 6.78 |
| BSU07150 | -2.04 | 3.75 | 16.5 | 4.9e-05 | 1.0e-03 | -4.1 | 9.92 |
| BSU35960 | -2.05 | 4.58 | 27.8 | 1.4e-07 | 4.3e-06 | -4.1 | 17.83 |
| BSU06660 | -2.11 | 6.06 | 12.3 | 4.5e-04 | 7.8e-03 | -4.3 | 6.99 |
| BSU35950 | -2.13 | 4.06 | 21.7 | 3.2e-06 | 8.1e-05 | -4.4 | 13.59 |
| BSU08460 | -2.14 | 3.83 | 10.7 | 1.1e-03 | 1.6e-02 | -4.4 | 5.93 |
| BSU35940 | -2.21 | 4.78 | 29.7 | 5.1e-08 | 1.7e-06 | -4.6 | 19.16 |
| BSU32130 | -2.23 | 7.62 | 39.7 | 3.0e-10 | 1.5e-08 | -4.7 | 25.95 |
| BSU39020 | -2.40 | 8.64 | 28.2 | 1.1e-07 | 3.6e-06 | -5.3 | 18.10 |
| BSU03670 | -2.49 | 5.72 | 24.4 | 8.0e-07 | 2.2e-05 | -5.6 | 15.49 |
| BSU11780 | -2.64 | -0.23 | 8.4 | 3.8e-03 | 4.8e-02 | -6.2 | 4.38 |
| BSU08440 | -2.77 | 5.02 | 28.4 | 1.0e-07 | 3.3e-06 | -6.8 | 18.22 |
| BSU22260 | -3.60 | 5.63 | 46.7 | 8.3e-12 | 5.0e-10 | -12.1 | 30.89 |
| BSU36070 | -4.36 | -0.96 | 8.4 | 3.7e-03 | 4.7e-02 | -20.5 | 4.42 |
| BSU12860 | -4.44 | 7.43 | 120.3 | 5.4e-28 | 8.7e-26 | -21.6 | 83.26 |
| BSU09680 | -5.04 | 7.24 | 127.0 | 1.9e-29 | 3.2e-27 | -33.0 | 88.00 |
| BSU06890 | -5.85 | -1.48 | 12.3 | 4.5e-04 | 7.9e-03 | -57.7 | 6.99 |
