# Supplementary material for: T-REx: Transcriptome analysis webserver for RNA-seq Expression data
Source: BMC Genomics. 2015 Sep 3;16(1):663. doi: 10.1186/s12864-015-1834-4 (PMC4558784; doi:10.1186/s12864-015-1834-4)
Supplement: Additional file 3: — Figure S3; k-means clustering of differentially expressed genes in the mutants. (ZIP 31925 kb) [file 12864_2015_1834_MOESM3_ESM.zip › Brinsmade.TMEV_TOPHITS_logFC.html]

|  | A\_F71Y-WT | B\_R61K-WT | C\_R61H-WT | Null-WT |
| --- | --- | --- | --- | --- |
| BSU03359 | 5.81854228631785 | -1.76102126688091 | 6.36028177469038 | 4.57475348290335 |
| BSU03350 | 5.31163063747002 | -1.06657048768142 | 5.61076290291042 | 4.10853743610537 |
| BSU32600 | 5.19947111852463 | 7.09797586762521 | 8.75962463491816 | 9.82305365494778 |
| BSU26830 | 4.94960364909713 | -0.243425251499388 | 5.41475216917428 | 3.72491789618169 |
| BSU32610 | 4.75666745302881 | 6.48855124752904 | 8.09528487606473 | 9.17117177416286 |
| BSU30700 | 4.45430542299422 | -0.17423389652003 | 4.43964783480523 | 2.92140031553318 |
| BSU03340 | 4.20470758021989 | -0.713120231114411 | 5.02433555424909 | 3.78569117515062 |
| BSU02160 | 3.90530520726753 | 4.36620865490375 | 4.56026741317268 | 3.04671116386519 |
| BSU09460 | 3.87090168056219 | 5.62523719133867 | 7.11631349827985 | 7.67929694735044 |
| BSU03360 | 3.64809646422632 | -1.62128116098681 | 4.88427132083401 | 2.99097729013844 |
| BSU02850 | 3.50046871155346 | -0.10225950245817 | 3.90655383491232 | 2.91902025470276 |
| BSU32590 | 3.40831342779737 | 5.9842745705733 | 7.73336384370222 | 8.93433610616005 |
| BSU28290 | 3.31228444141486 | 3.73582150561588 | 4.59922746759373 | 5.0452307270706 |
| BSU31540 | 3.26705238713192 | 4.77891988589241 | 6.14527551959672 | 6.31312843842836 |
| BSU32580 | 3.25379713372793 | 5.37292817713965 | 7.47426040721339 | 8.75970044877394 |
| BSU28300 | 3.21252481675222 | 3.7133413607446 | 4.50071449287278 | 4.93508695491588 |
| BSU27100 | 3.18042035087532 | 3.25792723603647 | 2.91773348528229 | 2.87653209119475 |
| BSU28280 | 3.13482247966086 | 3.82469012886943 | 4.60215060155925 | 5.14500735862654 |
| BSU28260 | 3.08863930533554 | 3.7723195974471 | 4.61852622942544 | 5.0513173472734 |
| BSU28250 | 3.064547564204 | 3.78804284262461 | 4.53920633586219 | 5.05028097786902 |
| BSU08880 | 3.04574652915241 | -0.523080947363587 | 4.85395139068164 | 2.68605774223788 |
| BSU28270 | 2.98357516829601 | 3.73724358489644 | 4.52037165038337 | 5.0181389393239 |
| BSU28310 | 2.92730935360521 | 3.60639084273959 | 4.34257619543232 | 4.77705355388596 |
| BSU02170 | 2.87834834814058 | 4.61906427420167 | 4.7837466339503 | 3.2560932720361 |
| BSU31550 | 2.87361033134126 | 4.45113359845482 | 5.7568906177511 | 5.84879456495448 |
| BSU02390 | 2.86763875735888 | 4.20044320918913 | 6.29106058158158 | 7.49763073116043 |
| BSU11360 | 2.76154752446206 | 4.14122321898953 | 4.8355580235173 | 4.97144666118183 |
| BSU19579 | 2.75281701761181 | 2.95826039936561 | 3.39590498768393 | 3.39692938679694 |
| BSU21870 | 2.7153814905939 | 3.5782401705512 | 4.12342990755881 | 4.22279175832919 |
| BSU32090 | 2.69178641655789 | 2.7748729867004 | 4.91701613063612 | 6.84673471410225 |
| BSU11390 | 2.5175452257588 | 3.86434256815205 | 4.57623773066336 | 4.72229221503926 |
| BSU11370 | 2.49715179749593 | 3.81973389570467 | 4.56547751260779 | 4.64777849660873 |
| BSU36070 | -2.47649147795836 | -1.91687895314974 | -2.7016739891719 | -4.35786862764901 |
| BSU19350 | 2.47106940428989 | 3.91168477066988 | 5.28457928342403 | 6.06370450648203 |
| BSU31560 | 2.44609494220275 | 4.15602893167292 | 5.49199996964544 | 5.66175680456131 |
| BSU11400 | 2.38437164423072 | 3.72621482366549 | 4.53569428151019 | 4.64645177028047 |
| BSU04400 | 2.26563156467345 | 1.14814684679348 | 1.16047347988464 | 1.61174787432283 |
| BSU08460 | -2.26415737273669 | -1.69757321154842 | -1.47999805173602 | -2.13851973879897 |
| BSU13940 | 2.26088277570281 | 2.73509642180928 | 4.23973054460611 | 5.48167911295559 |
| BSU08450 | -2.21043330445141 | -1.77550574606613 | -1.21902624912315 | -1.83245586892743 |
| BSU39340 | 2.08503321431679 | 2.24037856247446 | 3.19722482630711 | 3.62644745380239 |
| BSU37480 | 2.06731153725135 | 2.21415214744157 | 2.31193683496782 | 2.412482016964 |
| BSU12920 | 2.03516835153127 | 2.85890195983721 | 4.41717320449707 | 5.29171938989883 |
| BSU38770 | 2.01871766485577 | 2.69721745438794 | 2.85021053599406 | 3.00633285936937 |
| BSU15340 | 1.92279566199245 | 2.34510865710575 | 3.45319047026833 | 4.07774868557023 |
| BSU24090 | 1.91930995631388 | 1.82584172512951 | 1.68052207811328 | 2.06008541434505 |
| BSU02860 | 1.91410772743487 | -0.131752698667106 | 2.08622800927432 | 1.08099877462642 |
| BSU19580 | 1.88129996197495 | 3.07342767928621 | 3.45851934899955 | 3.48822199079559 |
| BSU03370 | -1.86828842626225 | -0.640826381360555 | -1.70622338385175 | -1.17713857113965 |
| BSU33310 | -1.85515200195508 | -1.90700786753359 | -1.41680821969328 | -1.89010098164185 |
| BSU09680 | -1.83753740391561 | -2.39162243908562 | -3.91058907424068 | -5.0423913592957 |
| BSU29600 | 1.8266099178047 | 2.20892504745612 | 0.817541220742135 | -0.411457774008807 |
| BSU12860 | -1.80882520887119 | -2.40929436499031 | -3.84657425451976 | -4.43502132732207 |
| BSU19040 | 1.80668379141457 | 1.96366442202221 | 2.47096815603876 | 2.58271873222555 |
| BSU38960 | 1.80263787644801 | 2.50497368714746 | 3.166879631122 | 3.39550466652741 |
| BSU39900 | 1.8020928800537 | 2.3047143599825 | 3.46442231533633 | 5.35529132895714 |
| BSU10460 | 1.79763988646729 | 3.75435511646933 | 5.04932647941125 | 5.85761223733958 |
| BSU27090 | -1.79242655517202 | -1.52506787986234 | -1.70323654745272 | -1.64909279019838 |
| BSU31970 | -1.78304168436542 | -1.21723876881133 | -1.19972082263689 | -1.80478931279198 |
| BSU10450 | 1.74862462418008 | 3.75027917789964 | 5.08655876427457 | 5.88236951839555 |
| BSU21770 | 1.74272611595811 | 1.81436497944491 | 2.23189912257022 | 2.61994321603571 |
| BSU17820 | 1.70298152949615 | 2.32849807071101 | 2.71279739525128 | 3.04977097499264 |
| BSU08440 | -1.6984988516183 | -1.91262015069365 | -1.76262406385222 | -2.77172915445137 |
| BSU32560 | 1.68969861818206 | 2.60161466912124 | 2.49636343552884 | 1.67783545816523 |
| BSU32550 | 1.68630445463608 | 3.19390218211128 | 5.16408817527052 | 6.4387559184327 |
| BSU39020 | -1.64914837517632 | -1.54615350049479 | -1.97789646831609 | -2.39565452810573 |
| BSU11780 | -1.6285863320597 | -2.90885190727284 | -2.97824649693953 | -2.63699551036896 |
| BSU09530 | 1.61670899517544 | 1.28945896060914 | 1.06230402259464 | 1.79424237712616 |
| BSU24070 | 1.61644807629078 | 1.59195141998302 | 1.41482124163294 | 1.69203914250382 |
| BSU29350 | 1.60334810720281 | 2.85167919975706 | 2.23373844081623 | 3.05255199543287 |
| BSU32570 | 1.59125264996719 | 3.68282810264555 | 5.93185465243559 | 7.25828830860928 |
| BSU37460 | 1.58516837175225 | 1.41765720658834 | 1.54371847348557 | 1.90234032431105 |
| BSU02240 | 1.58172644611075 | 1.99833369397028 | 2.61467275551231 | 2.91594948127261 |
| BSU22260 | -1.56857604710743 | -2.1837283605642 | -2.97775025421228 | -3.60247498839162 |
| BSU10230 | 1.54185438881662 | 1.72401925009232 | 1.4928180119829 | 1.09252654886833 |
| BSU10070 | 1.54112484881409 | 2.51661545392672 | 3.45783003010058 | 4.09094325542894 |
| BSU07150 | -1.54006756136826 | -1.81947114498463 | -1.87704779512243 | -2.03571303615496 |
| BSU37760 | 1.52355262100534 | 3.00166602139887 | 3.60318609280032 | 3.78781361473628 |
| BSU37770 | 1.52297837061859 | 2.25611166363953 | 2.81664624660581 | 2.9372939544884 |
| BSU39350 | 1.52170741331424 | 2.5861127805074 | 3.59972544026417 | 3.98379806624784 |
| BSU11410 | 1.5124825249605 | 2.31547444900801 | 3.07286559306797 | 2.74776828206193 |
| BSU02250 | 1.50927026655189 | 2.05800267781225 | 2.68300926771307 | 2.94436876645466 |
| BSU33320 | -1.49733588649548 | -1.4306268305032 | -1.20916877379612 | -1.60902605245804 |
| BSU03200 | 1.49540436907275 | 2.33972149892446 | 3.30441449885645 | 3.68906630352373 |
| BSU31570 | 1.47392493988918 | 3.83237476815731 | 5.04847070839317 | 5.1704217927302 |
| BSU03210 | 1.46050072821846 | 2.15751350175848 | 3.13913577300071 | 3.50447692130138 |
| BSU01600 | -1.45717993332961 | -1.01524241787271 | -1.4633266150406 | -1.88922586486986 |
| BSU24080 | 1.45409682424373 | 1.68983165244939 | 1.54835337690405 | 1.88241445169651 |
| BSU13580 | 1.42370382034946 | 1.59121315038573 | 1.68970092526501 | 1.83515316254103 |
| BSU03670 | -1.40833440386016 | -1.19054242871453 | -1.9991173956265 | -2.49454507219082 |
| BSU03790 | 1.40028600352565 | 2.05188938794814 | 2.69757984267933 | 3.34214729935024 |
| BSU19060 | 1.39306488905475 | 1.6260095712987 | 2.03145460310079 | 2.17189599728723 |
| BSU08820 | 1.38139810070011 | 1.39746898937563 | 1.98303430948016 | 1.97122301528272 |
| BSU28340 | 1.37415829390905 | 1.09851292664539 | 1.42233652756547 | 1.62862032133796 |
| BSU24990 | -1.36129755572384 | -3.07427815901731 | -1.34131241306061 | -0.972926006710481 |
| BSU24050 | 1.33720362441406 | 1.42482585232284 | 1.31559808010626 | 1.52842801305998 |
| BSU37470 | 1.33709959574857 | 1.36951222436092 | 1.62581229984555 | 1.63304792537351 |
| BSU24030 | 1.3359606181954 | 1.39132915834568 | 1.23334749919134 | 1.50165352788869 |
| BSU13570 | 1.33511879302603 | 1.08408265035795 | 1.39006635707478 | 1.64352773221897 |
| BSU18110 | 1.32417555811877 | 1.5554605563755 | 1.99909091710643 | 2.3971037119243 |
| BSU24060 | 1.32006337992148 | 1.48510090838705 | 1.33723749246769 | 1.57729928186176 |
| BSU07530 | 1.30673982430788 | 2.30851896327935 | 3.40943304688202 | 4.0067603153996 |
| BSU32950 | 1.30425496044346 | 1.27798637590748 | 1.26875357655314 | 1.91339190825222 |
| BSU11900 | 1.29964422339262 | 2.86605271914627 | 3.89930145629327 | 4.70008979702158 |
| BSU38930 | 1.29366581345124 | 2.20753316591967 | 2.85748526865923 | 3.05901485294501 |
| BSU01630 | -1.29136629399316 | -1.82185056340794 | -1.69559812719809 | -1.714999461046 |
| BSU12930 | 1.26901853660962 | 3.31968732095547 | 4.29965205408495 | 5.32850807846281 |
| BSU37680 | 1.26012450436191 | 1.68000647720928 | 1.96030822590517 | 1.93155024461882 |
| BSU10300 | 1.25565595186362 | 1.9966069581106 | 1.71363794350567 | 1.62235685037186 |
| BSU08830 | 1.24311533857422 | 2.18203421123655 | 2.55419525577574 | 2.0033033098708 |
| BSU08240 | -1.23737327487871 | -1.09484660958871 | -0.947395459267625 | -1.57580651038959 |
| BSU24040 | 1.23260710002627 | 1.3168644206807 | 1.24647183631525 | 1.44208465303978 |
| BSU11870 | 1.21730717939625 | 1.50389214314006 | 1.91018153214566 | 2.17328484874851 |
| BSU13180 | 1.21448795743835 | 1.69845371413838 | 2.5405134305796 | 2.95090478828495 |
| BSU08470 | 1.21366659398462 | 1.10132865411847 | 1.75975902759195 | 2.00637736534346 |
| BSU18550 | 1.20454020262989 | 1.4629537127056 | 2.39115992883279 | 2.76928159831826 |
| BSU40180 | 1.19394915707466 | 1.41057056994283 | 2.20701716205161 | 2.44759387735426 |
| BSU10410 | 1.1894539148952 | 2.79795904551285 | 4.03064879583484 | 4.86092293346629 |
| BSU04800 | 1.17663278498016 | 1.11159366395983 | 1.32121863948533 | 1.74597406486518 |
| BSU06660 | -1.1763695124951 | -0.466908808706767 | -0.877689165417744 | -2.10583463288487 |
| BSU31010 | 1.17186649574012 | 1.30446654047573 | 1.74232820538711 | 1.94248283267107 |
| BSU31000 | 1.16151979246246 | 1.34250254542376 | 1.70710363593631 | 1.83905711448297 |
| BSU06890 | -1.16060120394248 | -2.66776024844429 | -1.47681885076775 | -5.85000255520518 |
| BSU19050 | 1.15416355374374 | 1.44410020080124 | 1.90357916410712 | 2.09270046913635 |
| BSU36660 | 1.15357720366215 | 1.95066202082183 | 2.49171503569526 | 2.4784011797894 |
| BSU39890 | 1.15049923461701 | 2.53482950732912 | 3.75156394419878 | 5.58055862984311 |
| BSU35950 | -1.14482216829344 | -1.26865468615847 | -2.15963160112317 | -2.1342628699394 |
| BSU12430 | 1.12171661925689 | 1.17027224533765 | 1.73547907216907 | 1.95915838618821 |
| BSU24950 | -1.11502767211345 | -2.1687900204752 | -0.49734337400314 | -0.39258579652426 |
| BSU29310 | 1.10410040767306 | 0.787472907080915 | 1.41902447997636 | 1.77944553999083 |
| BSU29680 | 1.09662535318318 | 1.52167799159486 | 2.23652900162382 | 2.56138161017132 |
| BSU22660 | 1.08713957841916 | 2.22454989127785 | 1.21711421163058 | 1.40489430313707 |
| BSU11880 | 1.079728773496 | 1.65316364718149 | 2.13407799248518 | 2.32639429480307 |
| BSU03010 | 1.07899073456851 | 2.778250428862 | 5.47175696894404 | 6.92673313465123 |
| BSU40021 | 1.0760139357609 | 1.56278656211111 | 1.77980647490957 | 2.25271205217165 |
| BSU38090 | 1.06700343212052 | 1.85226269699893 | 2.93831875769424 | 3.72985392723237 |
| BSU13560 | 1.0621122781158 | 1.40547029268831 | 2.11422737059966 | 2.45236872309722 |
| BSU23680 | 1.0618574242664 | 0.817467064021274 | 1.22956042366531 | 1.08913011524496 |
| BSU21180 | 1.06157871642811 | 0.81637877528339 | 2.59411263467668 | 3.12093030926563 |
| BSU23670 | 1.05630742041213 | 0.813511479895941 | 1.07330714685933 | 1.22488323524503 |
| BSU18560 | 1.05598932647189 | 1.04510739545516 | 2.09607874568361 | 2.49644909540967 |
| BSU15100 | 1.052898104435 | 0.895859361532632 | 1.04732427862043 | 1.39956641152922 |
| BSU09990 | 1.04705736030482 | 0.990042853820342 | 1.49738818202862 | 1.7745124091166 |
| BSU11840 | 1.03647836672113 | 0.895554375705406 | 1.18369775242781 | 1.32089983543826 |
| BSU13850 | 1.03538904505381 | 1.14472561611377 | 0.857749165977318 | 1.18107802785361 |
| BSU13550 | 1.02603672372155 | 1.36302319174963 | 2.05201524506953 | 2.33762717667402 |
| BSU39610 | -1.02310893186878 | -1.55600218385313 | -1.29230162097161 | -1.89670762791071 |
| BSU14320 | 1.01355242112353 | 1.96679441436913 | 1.98152783205817 | 2.33077398380018 |
| BSU06310 | 1.01203115782914 | 2.0840130974713 | 2.6077026481548 | 2.98393242547901 |
| BSU37750 | 1.00934652541752 | 1.4369060909874 | 1.66668387702078 | 1.70243975764221 |
| BSU12960 | 0.999614135674769 | 2.18891657114642 | 3.18631768245105 | 4.31155521822042 |
| BSU15610 | 0.995044194218104 | 0.85532549791258 | 1.06454815896543 | 1.61567868738945 |
| BSU12940 | 0.991604132298406 | 3.50861934740714 | 4.34077697985919 | 5.46433089495396 |
| BSU31420 | 0.990381471137014 | 1.17666751190033 | 1.12916010153581 | 1.39698794596194 |
| BSU21190 | 0.982251747336801 | 0.576014670406566 | 2.87806764423401 | 3.44717591265234 |
| BSU22670 | 0.980619657669441 | 2.68423500385722 | 1.62808264409116 | 1.96029347032311 |
| BSU11010 | 0.975839256328578 | 1.7094153613201 | 2.54316861774339 | 2.99733583520696 |
| BSU38550 | 0.971181198359644 | 1.18388516923899 | 1.57343747392301 | 1.66645740520713 |
| BSU09670 | 0.968795141227491 | 1.01609006215466 | 1.47493809298442 | 1.51776756174462 |
| BSU06170 | 0.951700393658924 | 1.05305582246129 | 1.10240464326067 | 1.23274830932488 |
| BSU13620 | 0.950296808839931 | 1.01447244861805 | 1.43918748610339 | 1.62576404855188 |
| BSU15590 | 0.938530631633005 | 0.876141785403963 | 1.15922807393731 | 1.6611385621566 |
| BSU32260 | 0.935336918300697 | 1.35843008893046 | 1.9382979884795 | 2.37734690560588 |
| BSU11210 | 0.929904220730628 | 1.57180574033296 | 2.40004660148099 | 4.25146565310887 |
| BSU21410 | 0.928399643695904 | 1.11917930441184 | 1.7175282258459 | 2.87216463580233 |
| BSU07840 | 0.926456446840897 | 1.37555729844117 | 1.7607683164666 | 1.92799592718249 |
| BSU12400 | 0.92579942580332 | 1.84656634618524 | 3.02774408653308 | 3.07060950861143 |
| BSU31020 | 0.914568434558226 | 1.37603589665788 | 1.72690216510991 | 1.99476796993128 |
| BSU39910 | 0.890870094378311 | 1.13749326911455 | 2.18460746977487 | 4.0091861299055 |
| BSU15570 | 0.889228783547751 | 0.904892791389855 | 1.09991566463706 | 1.5482064319917 |
| BSU15600 | 0.888357587847435 | 0.897898228667312 | 1.16003404268721 | 1.63724029000018 |
| BSU32250 | 0.884621210265427 | 1.3529772199085 | 1.92605619302676 | 2.39341765532461 |
| BSU38270 | -0.883265250322081 | -1.33108216453068 | -0.984987743019174 | -1.31348127091058 |
| BSU29330 | 0.881069102010942 | 0.835842037919843 | 1.08744857204215 | 1.89698361741239 |
| BSU07580 | 0.877058944310318 | 1.00925732073281 | 1.42010023686631 | 1.61173851716994 |
| BSU34670 | 0.876017862386959 | 0.820164824663463 | 1.45721296058666 | 1.44608743574918 |
| BSU40022 | 0.873288820695461 | 1.04959393856972 | 1.58253638772349 | 1.92870412104405 |
| BSU12950 | 0.873144177097037 | 3.31315222248889 | 3.91715409810868 | 5.10680593565005 |
| BSU33730 | 0.858323327492686 | 2.05754317511061 | 1.78480640393406 | 3.24295751865324 |
| BSU03380 | -0.856349801906806 | -0.716916952646403 | -1.40210912680222 | -1.05964753849046 |
| BSU32070 | 0.855614760309233 | 1.26331060561184 | 2.57562454833961 | 4.4735901037498 |
| BSU21150 | 0.854977363594072 | 0.956215879304322 | 2.01510346263123 | 2.52150320578117 |
| BSU07590 | 0.854948293846856 | 0.975561454137045 | 1.23922687005234 | 1.59064743289633 |
| BSU07600 | 0.854689537714246 | 0.99636452072566 | 1.3994777091437 | 1.46797675184379 |
| BSU33770 | 0.853201560936455 | 1.25523413728949 | 1.86396084626707 | 2.69601872459841 |
| BSU40340 | 0.846962306175204 | 0.91017637490607 | 1.07857847580442 | 1.13998943402643 |
| BSU26650 | 0.842605505653577 | 1.53834366717273 | 0.624039215645579 | 1.14101999854396 |
| BSU22640 | 0.839953137454742 | 2.23686070245627 | 1.4339376045156 | 1.50992124707129 |
| BSU05870 | 0.837633194191582 | 1.63845066933468 | 2.27730296505865 | 2.42871988053025 |
| BSU22650 | 0.836342378590199 | 1.99722984452254 | 1.14382741102233 | 1.29443865139887 |
| BSU23070 | 0.830396695767052 | 0.964072873693502 | 1.19766577810297 | 1.44651993359475 |
| BSU18410 | 0.796071348471146 | 1.56954239734066 | 1.98410467246149 | 2.1975790428384 |
| BSU33830 | 0.795024810873538 | 1.15807952415746 | 1.34921507677353 | 1.48920987741618 |
| BSU12440 | 0.790492059214966 | 1.84946334061957 | 2.04412939066729 | 2.09723710790946 |
| BSU35960 | -0.784377552219102 | -1.21543281404852 | -1.93777089487054 | -2.04701222730582 |
| BSU13610 | 0.780602900750345 | 1.00570359030324 | 1.42185300444041 | 1.58489129357103 |
| BSU23820 | 0.776593899522137 | 1.10499470007342 | 1.36018099549678 | 1.31706407984626 |
| BSU35920 | -0.774098049523979 | -0.646715666475417 | -1.26974251454706 | -1.16387506898981 |
| BSU37660 | -0.769642763444844 | -1.1468601149887 | -1.4649124792542 | -1.48816782368784 |
| BSU18010 | 0.768643122446963 | 0.691669280853252 | 1.17483886578101 | 1.4768725961286 |
| BSU12520 | 0.765088929657305 | 0.413361855334179 | 1.59719475566045 | 1.79360354179857 |
| BSU40090 | 0.762747889709834 | 0.587132641325343 | 1.14610276001242 | 1.01781781847248 |
| BSU37740 | 0.762223607595259 | 1.59855818689039 | 1.68275656745352 | 1.25044718317253 |
| BSU12640 | -0.757821349604204 | -0.45514495545197 | 0.799468394494859 | 1.40104587073366 |
| BSU29470 | -0.746871873442324 | -1.0659897157913 | -1.66201898390961 | -1.83743404116267 |
| BSU35940 | -0.745888467325239 | -1.27148855759594 | -2.07010328045329 | -2.21400601929437 |
| BSU33710 | 0.741162298152774 | 1.96087215769962 | 1.94154437556768 | 3.33936868109715 |
| BSU15540 | -0.737739985157388 | -0.490819488289826 | -0.890193952658772 | -1.06594803628656 |
| BSU11860 | 0.729660364464517 | 0.877400470099211 | 1.16971058344103 | 1.33114087987812 |
| BSU36650 | 0.728539679818778 | 1.10553761506031 | 1.80390715865876 | 2.28790507600101 |
| BSU29990 | -0.724507697134221 | -0.52992116944629 | -1.42945479062937 | -1.83174147553687 |
| BSU34190 | 0.723169745881114 | 1.54308363410258 | 1.64760117260388 | 1.96368229671504 |
| BSU02410 | 0.717011873871467 | 1.26631202429029 | 1.28870733969403 | 1.07341004939034 |
| BSU14330 | 0.708171728607303 | 1.22317576462032 | 1.6159917341748 | 1.84561744993794 |
| BSU33760 | 0.70721359343334 | 1.06957985274192 | 1.51073661837468 | 2.52425474456622 |
| BSU02400 | 0.707139219344686 | 1.72799694477187 | 2.33201193651264 | 2.30554952321878 |
| BSU34660 | 0.696212075607548 | 1.45698881391201 | 1.66410001987865 | 2.06727724337399 |
| BSU05880 | 0.692633988954474 | 1.52651046522086 | 2.00785420562775 | 2.35042084290956 |
| BSU12619 | -0.688748764316951 | -0.00286225884316181 | 0.753126906965058 | 1.59060654459683 |
| BSU21750 | 0.682698679878782 | 0.657473423217912 | 1.11255732378301 | 1.26122927863163 |
| BSU18540 | 0.6692269432908 | 1.32905491506591 | 2.32100337276226 | 2.74838487339712 |
| BSU22680 | 0.663312498825109 | 1.88635999923879 | 1.24967263233728 | 1.24559097000803 |
| BSU13600 | 0.653892033240327 | 0.784797341729651 | 1.15949454282648 | 1.406742533692 |
| BSU32240 | 0.646991136479073 | 1.38209435711902 | 1.94624935276893 | 2.38816975787508 |
| BSU18000 | 0.637608920093855 | 0.768068659454753 | 1.32985364515112 | 1.80058345988877 |
| BSU12760 | -0.63283039355094 | -0.289330686034518 | 0.782841190232711 | 1.38917843033145 |
| BSU03230 | 0.631363535970901 | 0.640625560758311 | 1.21291955079238 | 1.33280877446563 |
| BSU38950 | 0.619074158231773 | 1.27434441731427 | 1.6997688192562 | 1.86806089484278 |
| BSU23560 | -0.616987121032207 | -0.628139444627911 | -1.16469257940398 | -1.16779116179615 |
| BSU15620 | 0.6167431251132 | 0.670787523904476 | 0.917437279425907 | 1.38132121907672 |
| BSU31480 | 0.611017568651769 | 1.49692789213007 | 1.65629644185262 | 2.0280987445769 |
| BSU33810 | 0.610601191306274 | 1.1370901881836 | 1.30039811346808 | 1.49103072563418 |
| BSU18570 | 0.602122843304875 | 0.794437107080191 | 1.2469012923167 | 1.56755771923517 |
| BSU12560 | 0.600192785222882 | 0.610633650001555 | 1.96258969408235 | 2.02495958636682 |
| BSU11250 | -0.599295004207415 | 0.226804339095965 | 0.719618934467439 | 1.95051895530732 |
| BSU35310 | 0.598855967102729 | 0.674417978212784 | 0.990065957635118 | 1.13185091724213 |
| BSU32980 | 0.594020259072216 | 0.91916619406975 | 1.10221304677843 | 1.38025079874667 |
| BSU40100 | 0.593871702380159 | 0.649455685212031 | 1.19702101098661 | 1.07360040017471 |
| BSU17130 | 0.591893264781742 | 1.3070965319302 | 2.31034540580976 | 3.04030106930932 |
| BSU39370 | 0.590255361675906 | 1.02383670621414 | 1.81681823062985 | 2.2119449023281 |
| BSU29700 | 0.580660845326877 | 0.734533997241705 | 1.07491536076343 | 1.47479119973092 |
| BSU21090 | 0.578673356309184 | 1.30658572961696 | 1.89406466889006 | 2.38850460067624 |
| BSU11190 | 0.576791002006736 | 0.925390144210489 | 1.95120734147482 | 2.88245457406559 |
| BSU15350 | 0.571985564900004 | 0.617749949164042 | 1.58111726587211 | 2.0555591386278 |
| BSU09590 | 0.562640571908193 | 0.750335276226689 | 0.898237124733223 | 1.06073916163846 |
| BSU15630 | 0.561353269596047 | 0.718231790756235 | 1.12153223802348 | 1.49309044501827 |
| BSU15530 | -0.54944898895249 | -0.513647688694735 | -0.831656380937077 | -1.03165573583266 |
| BSU12610 | -0.546859309916656 | -0.283890555832924 | 0.758208366594881 | 1.35247574510802 |
| BSU29440 | -0.545855104341179 | -0.500126686731177 | 0.483065454557519 | 1.54525330220245 |
| BSU17120 | -0.544982562857771 | -0.164766879294718 | 0.50968210232027 | 1.10607311888126 |
| BSU11230 | -0.538322640465798 | 0.670977014229235 | 3.12482167566819 | 5.28536501678399 |
| BSU27230 | 0.532579892156808 | 0.729927781988155 | 0.916639944398069 | 1.31142996332669 |
| BSU35930 | -0.532088354188107 | -0.832300971299378 | -1.85738922772353 | -1.71975263589652 |
| BSU15560 | -0.532028373049701 | -0.506878431366073 | -0.839176689397819 | -1.09821347341998 |
| BSU40320 | 0.530724142368939 | 0.939465479523719 | 1.06793009169279 | 1.16420591535479 |
| BSU33430 | 0.520444373076556 | 1.03842217500896 | 1.40220504401708 | 1.6950097549426 |
| BSU12810 | -0.518863347109594 | -0.217693590167573 | 0.833099717850176 | 1.42972067918365 |
| BSU31400 | 0.516963854110327 | 0.97238258642794 | 1.08547256153608 | 1.10162276396446 |
| BSU08780 | 0.515500842140345 | 0.719658105521905 | 1.12283176633406 | 1.42686210791525 |
| BSU11850 | 0.514038640301539 | 0.746825816423561 | 1.05609824925847 | 1.2167418116582 |
| BSU33440 | 0.50415108326229 | 0.959703323987792 | 1.3112051678971 | 1.57052503376404 |
| BSU26770 | 0.501964963338096 | 0.825959190697483 | 0.878096168831753 | 1.39808070635563 |
| BSU29690 | 0.497415902369694 | 0.432134372223447 | 1.12849358100452 | 1.24179696362863 |
| BSU15550 | -0.486082029158792 | -0.454976261758938 | -0.848742431648297 | -1.01398798429587 |
| BSU12630 | -0.481710280649683 | -0.130324227675484 | 0.909331327274848 | 1.4601482709257 |
| BSU32130 | -0.48080085104781 | -0.668535378019209 | -2.00447721939583 | -2.22604309421486 |
| BSU15520 | -0.474012945894245 | -0.464627852162869 | -0.839562021376496 | -1.03379430440573 |
| BSU01910 | -0.469681063789071 | -0.116340845369636 | 0.594594807520793 | 1.44968938224626 |
| BSU05840 | -0.457713844483683 | -0.859872086687838 | -0.996758496097927 | -1.40327337348542 |
| BSU13590 | 0.450441195904745 | 0.778236489959453 | 1.2453650040239 | 1.30832173663904 |
| BSU15580 | 0.44671830056926 | 1.01492216911558 | 1.14905537428346 | 1.58290054267667 |
| BSU18120 | -0.445007689396828 | -0.534684155935419 | -1.21978769075854 | -1.53080007213043 |
| BSU12460 | 0.432294088025671 | 0.414778360150915 | 1.46303864573824 | 1.74573795012246 |
| BSU39880 | 0.418429190893732 | 1.15245730673225 | 2.20584256767028 | 3.86405291892233 |
| BSU29140 | 0.416345104134627 | 0.363904059221105 | 0.626037941303472 | 1.01111083874852 |
| BSU02780 | 0.401528758938819 | 0.501988595162867 | 0.930116386810367 | 1.02487524247204 |
| BSU12990 | 0.392253183072827 | 1.19082162697208 | 2.25900617015079 | 3.24070917451589 |
| BSU12650 | -0.392029903890515 | -0.30867092108867 | 0.846741806771827 | 1.37313645545811 |
| BSU28240 | 0.39070691509858 | 0.452442264745659 | 0.829650895273805 | 1.10643647429482 |
| BSU00090 | -0.386117363311402 | -0.713682028658492 | -1.31735443470087 | -1.82107810121339 |
| BSU13190 | 0.385529911953168 | 0.905536222504729 | 1.80132310449088 | 2.53042800835124 |
| BSU03220 | 0.372780974248483 | 1.49912824307799 | 2.42022682788631 | 2.73897456578329 |
| BSU17210 | 0.367092236136395 | 0.756469495721535 | 1.34090615863526 | 1.78365617263534 |
| BSU29450 | -0.363592246701117 | -0.364518090631885 | 0.74373998964318 | 1.70719389477664 |
| BSU21220 | 0.36278294287344 | -0.0602024518331389 | 0.923950545101297 | 1.77746099172593 |
| BSU39380 | 0.360604707348663 | 1.04251959360679 | 1.58318113778437 | 1.91668061071956 |
| BSU15490 | -0.348994673121093 | -0.460831728850886 | -0.833809625572705 | -1.05300407984151 |
| BSU02490 | 0.346862432062917 | 1.26241734309473 | 1.74160446315806 | 1.44017622442576 |
| BSU15510 | -0.330671380324978 | -0.519995910877037 | -0.824827216620284 | -1.04785032653112 |
| BSU38450 | 0.326794253204442 | 0.229220527910444 | 0.562329895488109 | 2.16360670058364 |
| BSU31450 | 0.31843746113182 | 0.580896433014583 | 1.20923894401138 | 1.63693290152343 |
| BSU37560 | -0.307507896281202 | -0.125738922854882 | 1.97021726951075 | 0.684903597756389 |
| BSU15500 | -0.303240471727288 | -0.514848701098543 | -0.802744589992559 | -1.04997337267214 |
| BSU18450 | -0.300765523899059 | 0.163857574910403 | 1.30979939674563 | 1.95305532991509 |
| BSU32080 | -0.299245030365783 | 1.07793982937333 | 2.57436893068828 | 4.73813480570192 |
| BSU06420 | -0.299135306971798 | -0.34592799607077 | -0.871017753605442 | -1.02629958570674 |
| BSU39360 | 0.295966030037813 | 1.26018401136153 | 2.02793994319691 | 2.51748198600881 |
| BSU11890 | 0.287393966108836 | 0.830902764074844 | 1.68973457184387 | 2.40391491074077 |
| BSU12700 | -0.286548265486277 | -0.085743529875382 | 0.441870838995549 | 1.23503820304671 |
| BSU17150 | -0.282365921114858 | 0.976713388294289 | 1.93699499335933 | 2.84455549426497 |
| BSU38460 | 0.269856652849434 | 0.0080552728070218 | 0.532508810549217 | 1.55197987864247 |
| BSU33800 | 0.269257739091942 | 1.33093978957674 | 1.42971099704557 | 1.64495272901557 |
| BSU38810 | -0.268909972721231 | -0.540533816501735 | -0.988840856018109 | -1.25232522683797 |
| BSU17220 | 0.265439189115674 | 0.58385413108454 | 1.06172476349342 | 1.46152550998272 |
| BSU14700 | -0.254359578290152 | -0.362449495829186 | -0.593584949574198 | -1.05427807794081 |
| BSU11200 | 0.253174653571757 | 0.718740598406406 | 2.41018414467075 | 3.56373945673079 |
| BSU12780 | -0.236520682340918 | -0.408175357678888 | 0.802421681669143 | 1.43599514887916 |
| BSU27200 | 0.23645915675307 | 0.732742923128325 | 1.24489693311677 | 1.61505394010665 |
| BSU12570 | -0.230015313799239 | -0.0807023118793691 | 0.802704258650202 | 1.49105685406424 |
| BSU27220 | 0.228852673321853 | 0.561882579632505 | 0.903941688137816 | 1.46651001320832 |
| BSU33700 | 0.226138528051418 | 2.1255860717182 | 1.92898798037919 | 3.30428014839492 |
| BSU09450 | 0.221765500379777 | 0.518432420823487 | 1.12028776072653 | 1.45552819624127 |
| BSU12970 | 0.221585488062314 | 1.10698785073858 | 2.38302023354071 | 3.33877897864588 |
| BSU11220 | 0.206814146625171 | 0.895264246234388 | 2.9496055503575 | 4.431884210218 |
| BSU12980 | 0.202073741699522 | 1.5250367561749 | 2.49244177521812 | 3.69744273081034 |
| BSU12590 | -0.199084801082353 | -0.0805772370828899 | 0.952844842325129 | 1.46937859018308 |
| BSU05860 | 0.197534767319766 | 1.0196213872645 | 1.22962723711561 | 1.48748451342954 |
| BSU11240 | -0.194612144061293 | -0.000776023011401638 | 1.40938108375712 | 3.22973333091024 |
| BSU39920 | 0.193887203307035 | 0.47606584719938 | 1.32262806419322 | 2.77765369398584 |
| BSU10390 | 0.187179532902947 | 1.6486677372028 | 1.89422194191036 | 2.50780312990385 |
| BSU38470 | 0.186105526099733 | 0.82126111916688 | 1.26907728132981 | 1.59801071672848 |
| BSU40420 | -0.180517514848279 | -0.543430009336784 | -1.15382940941963 | -1.50485868357166 |
| BSU17160 | -0.176219141369392 | 0.812570077225897 | 1.6374723596151 | 2.60626743128632 |
| BSU33720 | 0.174269751557906 | 1.88034359272328 | 1.80762003626617 | 3.13887371438431 |
| BSU12580 | -0.165485871470658 | -0.0551066575183959 | 1.02423014001394 | 1.44435121977575 |
| BSU17170 | 0.15651023871682 | 0.878528744490713 | 1.67607956910223 | 2.6301488601484 |
| BSU12649 | -0.155777878524711 | -0.473688051577784 | 0.763018657776494 | 1.46065442781835 |
| BSU32510 | -0.151389766736321 | 0.849172396189985 | 1.07477551808768 | 2.11678344234077 |
| BSU17140 | 0.148837153574921 | 1.26486110464626 | 2.30701706072748 | 3.07979957614784 |
| BSU32520 | -0.147989164877114 | 0.678596411792278 | 1.60286886181327 | 2.64309040965787 |
| BSU10570 | -0.144784237460144 | 0.773619784309057 | 1.45088789808168 | 2.25782771526293 |
| BSU12660 | -0.139864793133251 | -0.0253454403523691 | 1.13484438789812 | 1.50003806509686 |
| BSU39390 | -0.131456096651371 | 1.09409172265729 | 1.7419663782401 | 1.99895101602174 |
| BSU09440 | 0.12373931121778 | 0.524081828408307 | 1.04438236721636 | 1.31554288252115 |
| BSU17180 | -0.120406377229364 | 0.863597658160181 | 1.79341741024458 | 2.54803089081716 |
| BSU18440 | -0.115217952740421 | 0.248604546174838 | 1.33416419159212 | 1.99236925566472 |
| BSU03020 | -0.107399594846108 | 0.729919130790364 | 2.66100352252756 | 4.16714129935678 |
| BSU12770 | -0.0956318026631321 | -0.524797350021957 | 0.958696088190152 | 1.5805434028729 |
| BSU17190 | 0.0701199044229008 | 0.776105807311715 | 1.58934308197243 | 2.29137306666488 |
| BSU17200 | 0.0661511489521166 | 0.504975596983675 | 1.29925645613804 | 1.87015733328799 |
| BSU12750 | 0.0657910176872147 | 0.0193973741137033 | 0.782985021858919 | 1.40688618021567 |
| BSU03530 | 0.0621035055050389 | 1.3637943041005 | 1.65059883353568 | 1.83986209047384 |
| BSU36640 | 0.0556617757350394 | 0.946049727040758 | 1.04077058939047 | 1.30543703401903 |
| BSU39870 | 0.0450423350617583 | 0.355632333265446 | 1.07896966186563 | 2.500888921172 |
| BSU12800 | -0.0341927313615263 | 0.135622915800124 | 1.23870695222865 | 1.73056680764038 |
| BSU12600 | 0.0290808838746833 | -0.171878815191546 | 1.02720575276993 | 1.54689335797051 |
| BSU39930 | 0.0281495333774274 | 0.289388737365277 | 1.00797041223381 | 2.63834504418494 |
| BSU32530 | 0.0143141298781585 | 0.853387449885718 | 2.13973073860539 | 3.35264826651821 |
| BSU03030 | -0.0106869325653492 | 0.251299594579649 | 0.69106683388147 | 1.30697752315289 |
| BSU13000 | -0.00984936738748062 | 1.18092335178527 | 2.09496278871184 | 3.27648347360213 |
| BSU21229 | 0.00802567082441628 | 0.532501688012462 | 1.35299227615762 | 2.06971704742701 |
