# Supplementary material for: T-REx: Transcriptome analysis webserver for RNA-seq Expression data
Source: BMC Genomics. 2015 Sep 3;16(1):663. doi: 10.1186/s12864-015-1834-4 (PMC4558784; doi:10.1186/s12864-015-1834-4)
Supplement: Additional file 3: — Figure S3; k-means clustering of differentially expressed genes in the mutants. (ZIP 31925 kb) [file 12864_2015_1834_MOESM3_ESM.zip › Brinsmade.contrasts_cohesion.high.html]

**Cohesion of Contrasts**  
  
  
  
  
  
  
  
  
  
  

| A\_F71Y-WT | B\_R61K-WT | C\_R61H-WT | Null-WT | GeneCount | Download Genes | Group\_ID | Cohesion\_ID |
| --- | --- | --- | --- | --- | --- | --- | --- |
|  |  |  |  |  |  |  |  |
| --- | --- | --- | --- | --- | --- | --- | --- |
|  |  | XXX | XXX | 46 | genes | 4 | C\_R61H-WT\|Null-WT |
|  |  |  | XXX | 44 | genes | 3 | Null-WT |
|  | XXX | XXX | XXX | 32 | genes | 1 | B\_R61K-WT\|C\_R61H-WT\|Null-WT |
| XXX | XXX | XXX | XXX | 32 | genes | 5 | A\_F71Y-WT\|B\_R61K-WT\|C\_R61H-WT\|Null-WT |
|  | XXX |  |  | 7 | genes | 2 | B\_R61K-WT |
|  |  | XXX |  | 5 | genes | 9 | C\_R61H-WT |
| XXX |  | XXX |  | 1 | genes | 6 | A\_F71Y-WT\|C\_R61H-WT |
|  | XXX | XXX |  | 1 | genes | 7 | B\_R61K-WT\|C\_R61H-WT |
|  | XXX |  | XXX | 1 | genes | 8 | B\_R61K-WT\|Null-WT |
