# Supplementary material for: T-REx: Transcriptome analysis webserver for RNA-seq Expression data
Source: BMC Genomics. 2015 Sep 3;16(1):663. doi: 10.1186/s12864-015-1834-4 (PMC4558784; doi:10.1186/s12864-015-1834-4)
Supplement: Additional file 3: — Figure S3; k-means clustering of differentially expressed genes in the mutants. (ZIP 31925 kb) [file 12864_2015_1834_MOESM3_ESM.zip › 00.overviewtable.html]

# T-REx results of Brinsmade

**Tutorial for Interpretation of T-REx Results**


---

|  |  |  |  |  |  |  |  |  |  |  |  |  |
| --- | --- | --- | --- | --- | --- | --- | --- | --- | --- | --- | --- | --- |
||  |  |  |  |  |  |  |  |
| --- | --- | --- | --- | --- | --- | --- | --- |
| **Global analysis** | | | | | | | |
| DATA Table  Factors Table  Contrasts  Class data  Download results | \_\_\_\_\_\_\_\_\_ | Library size | MDS plot | Box plot of normalized signals |

  

|  |  |  |  |  |  |  |  |  |  |  |  |  |  |  |  |  |  |  |  |  |
| --- | --- | --- | --- | --- | --- | --- | --- | --- | --- | --- | --- | --- | --- | --- | --- | --- | --- | --- | --- | --- |
||  |  |  |  |  |  |  |  |
| --- | --- | --- | --- | --- | --- | --- | --- |
| **Individual Contrasts analysis** | | | | | | | |
| Volcano plots | NEW: Volcano plots Interactive mining | Significant Changed Genes | MA plots |
|  |
| Differential Expression Each Contrast | Differential Expression All Contrasts | Heatmap TopHits | Heatmap TopHits, High Fold |
| Data table | Data table | -Heatmap TopHits data  -Heatmap Cluster data | -Heatmap High Fold data  -Heatmap Cluster data |

  

|  |  |  |  |  |  |  |  |  |  |  |  |  |  |  |  |  |  |  |  |  |  |  |  |  |  |  |  |  |  |  |  |  |  |  |  |  |  |  |  |
| --- | --- | --- | --- | --- | --- | --- | --- | --- | --- | --- | --- | --- | --- | --- | --- | --- | --- | --- | --- | --- | --- | --- | --- | --- | --- | --- | --- | --- | --- | --- | --- | --- | --- | --- | --- | --- | --- | --- | --- |
||  |  |  |  |  |  |  |  |
| --- | --- | --- | --- | --- | --- | --- | --- |
| **Experiment Analysis** | | | | | | | |
| Correlation matrix of Experiments | Heatmap of Experiments | Gene Network Significant Changed Genes | Gene Network High Fold Changed Genes |  |
| Correlation data | Heatmap data | Gene Network data | Gene Network High FC data |  |
|  |
| Contrasts Cohesion Genes overlap of Contrasts | Contrasts Cohesion Gene list | Contrasts Cohesion High Fold Change | Contrasts Cohesion Gene List |  |
| Matrix data |  | Matrix data |  |  |
|  |
| Clustering Median FOLD k-means | Clustering High FOLD k-means | Clustering Mean SIGNALS k-means | Dendrogram Median Fold k-means | Dendrogram High Fold k-means |
| ClusterIDs and data | ClusterIDs and data | ClusterIDs and data |  |  |

  

|  |  |  |  |  |  |  |  |  |  |  |  |  |  |  |  |  |  |  |  |  |  |  |  |  |  |  |  |  |
| --- | --- | --- | --- | --- | --- | --- | --- | --- | --- | --- | --- | --- | --- | --- | --- | --- | --- | --- | --- | --- | --- | --- | --- | --- | --- | --- | --- | --- |
||  |  |  |  |  |  |  |  |
| --- | --- | --- | --- | --- | --- | --- | --- |
| **Analysis of the Classes** | | | | | | | |
| k-means RATIO  Class TopHits | k-means SIGNAL  Class TopHits | Mean SIGNAL Plots Each Class | Correlation matrix All Classes | Correlation matrices Each Class |
| ClusterIDs and data | ClusterIDs and data | Mean SIGNAL data | Correlation data |  |
|  |
| Heatmap Class TopHits | Heatmap Class Signals | Heatmaps Each Class Ratio | Heatmaps Each Class Signals | MDS plot of k-means of ClassTophits |
| - Heatmap data  - ClusterIDs | - Heatmap Signal data  - ClusterIDs | Link to ClusterIDs | Link to ClusterIDs |  |

  

|  |  |  |  |  |  |  |  |  |  |  |  |  |  |  |  |  |  |  |  |  |  |  |  |  |  |  |  |  |  |  |  |  |  |  |  |  |  |  |  |  |  |  |  |  |  |  |  |  |  |  |  |  |  |  |  |  |  |  |  |  |  |  |  |  |  |  |  |  |  |  |  |  |  |  |  |  |  |
| --- | --- | --- | --- | --- | --- | --- | --- | --- | --- | --- | --- | --- | --- | --- | --- | --- | --- | --- | --- | --- | --- | --- | --- | --- | --- | --- | --- | --- | --- | --- | --- | --- | --- | --- | --- | --- | --- | --- | --- | --- | --- | --- | --- | --- | --- | --- | --- | --- | --- | --- | --- | --- | --- | --- | --- | --- | --- | --- | --- | --- | --- | --- | --- | --- | --- | --- | --- | --- | --- | --- | --- | --- | --- | --- | --- | --- | --- |
||  |  |
| --- | --- |
| **Data tables** | |
| Link to file | Description |
| Class TopHits ClusterIDs | Table of Class TopHits and there ClusterID |
| Class TopHits High Fold | Table of Class HighFold: Fold Change > 5, p-value < 0.01 |
| Class TopHits logFC | Table of Class and Log2 Fold Change values. TopHits= Fold Change > 5 and p-value<0.05 |
| Class TopHits mean signals | Table of Class TopHits Signals |
| Contrasts Overview | Overview of COntrasts and link to TopHits tables of each Contrast |
| Correlation Matrix Classes | Correlation between Class members over Experiments |
| Correlation Matrix Experiments | Correlation between Experiments |
| Differential Expression all Contrasts | DE table, all values |
| Differential Expression all Contrasts logFC | DE table TopHits= Fold Change > 2 and p-value<0.05 |
| Differential Expression all Contrasts logFC High Fold | DE table High Fold: Fold Change > 5, p-value < 0.01 |
| Filenames in JSON format | Summery JSON formmated file with filenames for downstream processing |
| Filenames of Class TopHits k-means | Overview of k-means clusters |
| Filenames of Correlation Matrix of Classes | Filenames |
| Filenames of Heatmaps Class TopHits | File with filenames of all heatmaps of each Class group |
| Filenames of MA Plots | Filenames |
| Filenames of Mean Signals Classes | Mean Signals of Genes |
| Filenames of Mean Signals Classes log2 | Filenames |
| Filenames of Mean signals Classes TopHits | Mean Signals of Genes |
| Filenames of Significant changed Genes | Complete table with Significant changed genes |
| Filenames of Volcano Plot data | Filenames |
| Filenames of k-means High Fold | Filenames |
| Filenames of k-means on Fold change | Filenames |
| Gene Network Edges | Gene network for Cytoscape of Changed genes |
| Gene Network Edges High Fold | Gene network for Cytoscape of HighFold Changed genes |
| Heatmap of Class Signals | Heatmap of Class Signals |
| Heatmap of Class Tophits | Heatmap of Class TopHits |
| Heatmap of Experiments | Heatmap of Experiments |
| Heatmap of High Fold Tophits | Heatmap of HighFold TopHits of all Contrasts |
| Heatmap of Tophits | Heatmap of TopHits of all Contrasts |
| Mean signals log2 | Log2 Signals normalized |
| Mean signals log2 Scaled | Log2 Signals Global scaled |
| Mean signals log2 Scaled per Class | Log2 Signals Scaled per Class |
| Mean signals log2 Scaled per Gene | Log2 Signals Scaled per Gene |
| Mean signals of replicates | Mean Signal of replicates |
| Normalised Data Table | Normalized data |
| k-means High Fold ClusterIDs | k-means Cluster IDs |
| k-means on Fold change ClusterIDs | Table with Cluster IDs |
