# Supplementary material for: T-REx: Transcriptome analysis webserver for RNA-seq Expression data
Source: BMC Genomics. 2015 Sep 3;16(1):663. doi: 10.1186/s12864-015-1834-4 (PMC4558784; doi:10.1186/s12864-015-1834-4)
Supplement: Additional file 3: — Figure S3; k-means clustering of differentially expressed genes in the mutants. (ZIP 31925 kb) [file 12864_2015_1834_MOESM3_ESM.zip › Brinsmade.ClassHeatmaps.clusters.list.html]

GeneIDs and their ClusterID can be found via the links below

---

Class ArgR  
Class CcpA  
Class CodY  
Class CodY\_CcpA  
Class Complex  
Class MalR
